# Conformational Analysis of Acyclic α‐Fluoro Sulfur Motifs

**DOI:** 10.1002/chem.202003361

**Published:** 2020-09-28

**Authors:** Nathalie Erdeljac, Christian Mück‐Lichtenfeld, Constantin G. Daniliuc, Ryan Gilmour

**Affiliations:** ^1^ Organisch-Chemisches Institut Westfälische Wilhelms-Universität Münster Corrensstraße 36 48149 Münster Germany

**Keywords:** conformational analysis, crystallography, fluorine, *gauche* effect, stereoelectronics

## Abstract

Bioactive small molecules containing α‐fluoro sulfur motifs [RS(O)_n_CH_2_F] are appearing with increasing frequency in the pharmaceutical and agrochemical sectors. Prominent examples include the anti‐asthma drug Flovent^®^ and the phenylpyrazole insecticide pyrafluprole. Given the popularity of these structural units in bioactive small molecule design, together with the varying oxidation states of sulfur, a conformational analysis of α‐fluoro sulfides, sulfoxides, and sulfones, would be instructive in order to delineate the non‐covalent interactions that manifest themselves in structure. A combined crystallographic and computational analysis demonstrates the importance of hyperconjugative donor‐acceptor interactions in achieving acyclic conformational control. The conformational disparity in the *syn*‐ and *anti*‐diastereoisomers of α‐fluorosulfoxides is particularly noteworthy.

## Graphical abstract

**Hyperconjugation**: Given the importance of α‐fluoro sulfur motifs in bioactive small molecules, together with the varying oxidation states of sulfur, a conformational analysis of α‐fluoro sulfides, sulfoxides and sulfones is disclosed in order to delineate the non‐covalent interactions that manifest themselves in structure. A combined crystallographic and computational analysis demonstrates the importance of hyperconjugative donor–acceptor interactions in achieving acyclic conformational control.

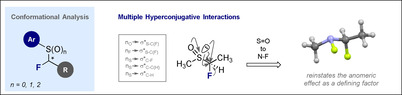

## Introduction

**Figure 1:**
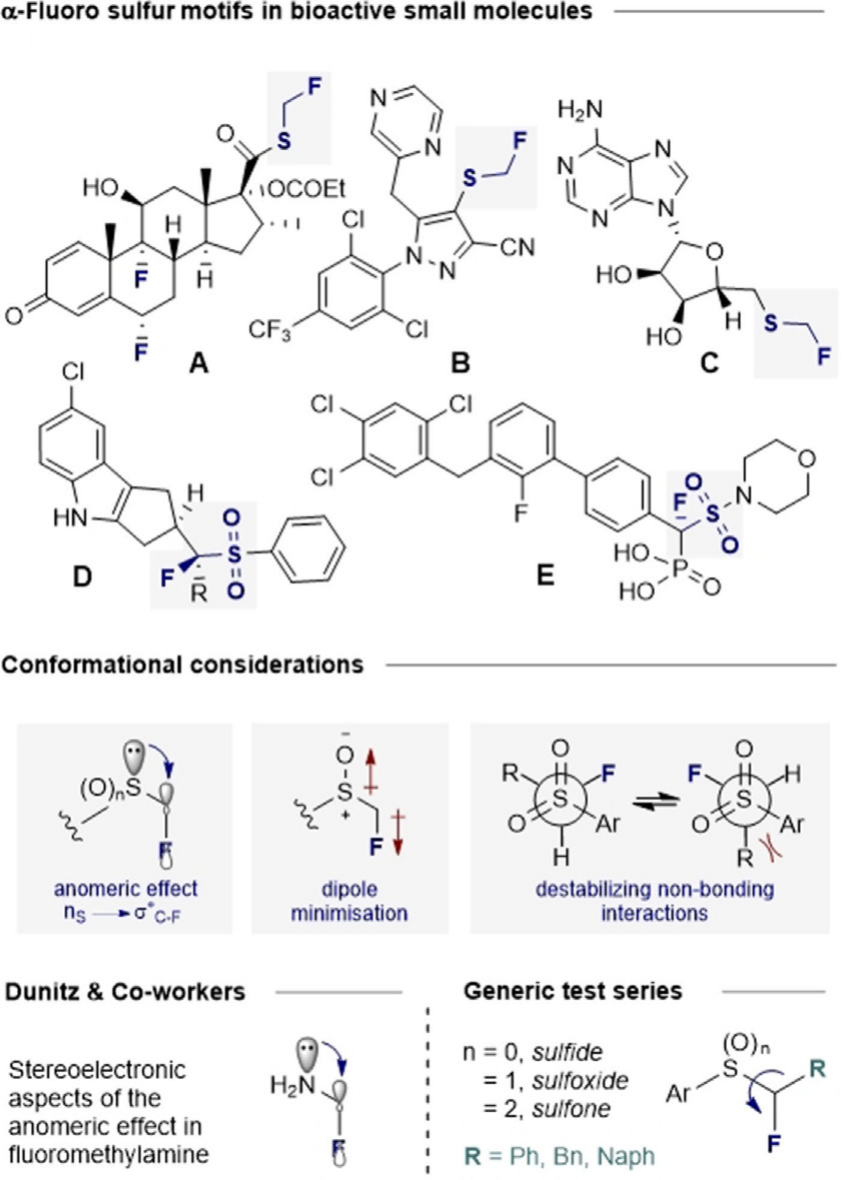
Top: Examples of bioactive molecules containing the α‐fluoro sulfur motif. (A) Anti‐inflammatory agent fluticasone propionate (Flovent^®^).^[11]^ (B) Insecticidal agent pyrafluprole.^[12]^ (C) 5'‐(monofluoromethylthio)adenosine (MFMTA).^[13]^ (D) LXR modulator.^[14]^ (E) Striatal‐enriched protein tyrosine phosphatase (STEP) inhibitor.^[15]^ Centre: Intramolecular interactions under consideration. Bottom: The anomeric effect in fluoromethylamine and the generic test series investigated.

Molecular editing with fluorine is an expansive strategy to modulate the physicochemical contours of structure‐function interplay.^[1]^ The strategic value of fluorination transcends classical boundaries and continues to underpin innovation in the fields of enantioselective catalysis,^[2]^ agrochemistry^[3]^ and medicinal chemistry (Figure 1, top).^[4]^ This translational impact is most apparent in the context of bioisosterism, where direct‐ (C−H) or deoxy‐fluorination enables the local electronic environment to be influenced with only nominal steric fluctuation.^[5]^ Although subtle, this process can elicit striking effects, including modulated lipophilicity, intrinsic potency, metabolic stability and pKa values.^[6]^ In addition, the ionic character of the C−F^(δ−)^ bond facilitates stabilising electrostatic interactions with proximal electropositive groups, as is best represented by the diaxial orientation of *cis*‐3,5‐difluoropiperidine.^[7]^ Moreover, the low‐lying antibonding orbital (σ_C‐F_*) readily engages in stabilising stereoelectronic interactions with a plenum of donors (e.g. π‐systems, C−H bonds or O, N lone pairs).[^1^, ^2a^, ^2c^] A prominent example of this phenomenon is the stereoelectronic analysis of the anomeric effect in fluoromethylamine reported by Dunitz and co‐workers.^[8]^ Conformations in which donor‐acceptor orbital overlap (e.g. σ, π or n_O/N_→σ_C‐F_*) is optimal frequently dominate, thus rendering judicious introduction of fluorine valuable in enabling conformational control (Figure 1, centre). A venerable example of this phenomenon is the fluorine *gauche* effect, in which stabilising donor (σ_C‐H_)→acceptor (σ_C‐F_*) interactions give rise to the *synclinal* conformation of 1,2‐difluoroethane.^[9]^ The generality of this effect is well established such that F‐C−C‐X motif (X=electron‐withdrawing group) prevails in the absence of overriding steric factors.[^1^, ^2^, ^9^] Most recently, and pertinent to this study, examples of sulfur (sulfoxides and sulfones) effects have been disclosed.^[10]^ To complement these investigations on the β‐fluoro‐sulfur fragment, it was envisaged that a conformational analysis of the α‐fluoro‐sulfur motif would be valuable given its prominence in bioactive small molecules (Figure 1).

Representative examples from the fields of agrochemistry and pharmaceutical and agrochemistry are illustrated in Figure 1 A–E.[^11^, ^12^, ^13^, ^14^, ^15^] However, unlike β‐fluoro sulfur motifs, interrogation of the α‐fluoro system must consider the related anomeric effect (Figure 1, centre) inherent to systems that permit hyperconjugative n_S_→σ^*^ interactions.[^16^, ^17^, ^18^]

A recent computational study has established that it is feasible for both the *gauche* and the anomeric effects to operate synergistically in cyclic α‐substituted sulfoxides.^[19]^ To complement this theoretical study, and expand upon our previous investigations pertaining to the β‐fluoro sulfur motif, a conformational analysis of acyclic F‐C_α_‐S(O)_*n*_ (*n=*0, 1, 2) units is disclosed.^[20]^ To that end, a series of fluorinated and non‐fluorinated compounds were prepared and their conformational behaviour interrogated by a combined crystallographic and computational analysis (Figure 1, bottom).

## Results and Discussion

### Synthesis of target compounds

**Scheme 1:**
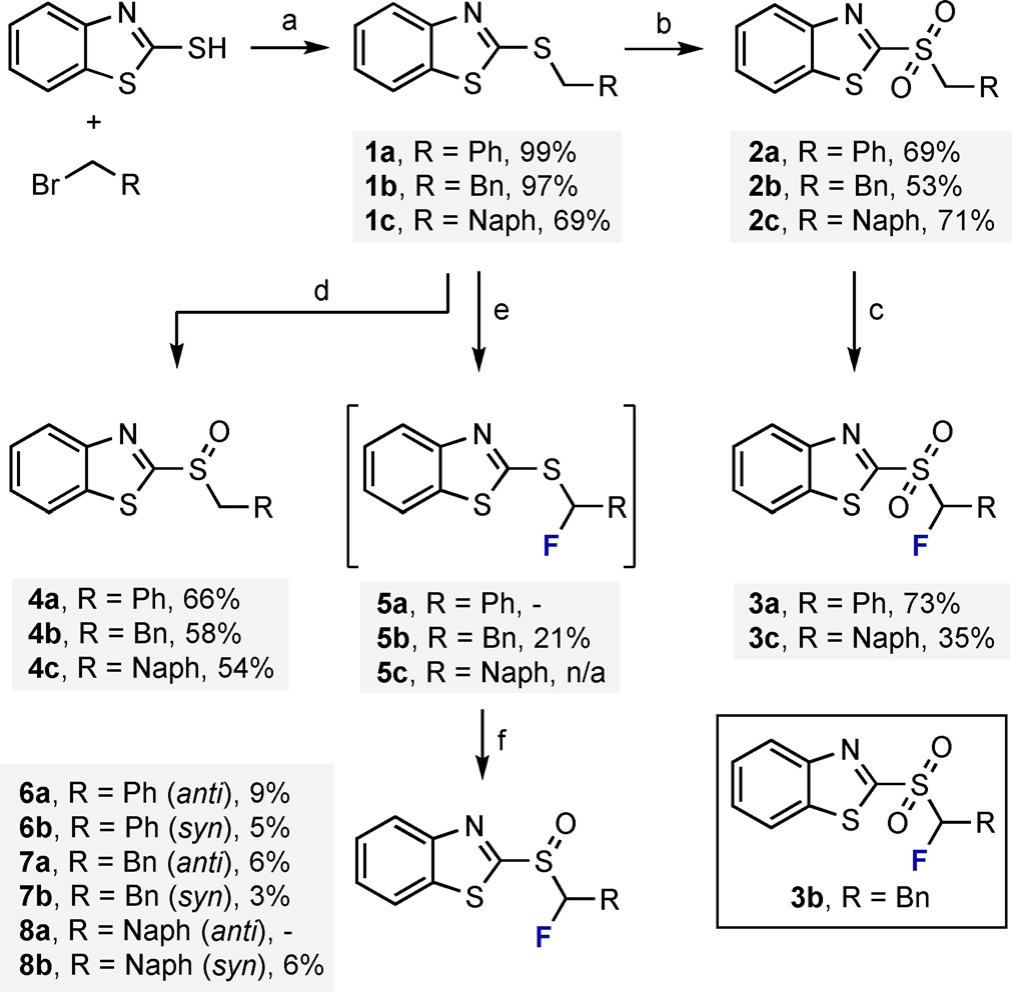
Synthesis of target compounds. (a) NaH, DMF, 0 °C to RT, o/n; (b) NaIO_4_, RuCl_3_⋅*x* H_2_O, MeCN/CHCl_3_/H_2_O, RT, o/n; (c) NFSI, ZnCl_2_, LiHMDS, THF, RT, 5 h; (d) *m*‐CPBA, CH_2_Cl_2_, 0 °C to RT, 24 h; (e) i) Selectfluor^®^, 10 min., ii) Et_3_N, 10 min, MeCN; (f) *m*‐CPBA, CH_2_Cl_2_, RT, o/n.

To facilitate direct comparison between the test sets (Figure 1, bottom), a scaffold based on the mercaptobenzothiazole core was conceived (Scheme 1). To that end, sulfides **1 a**–**c** were prepared by direct alkylation of 2‐mercaptobenzothiazole with the respective alkyl bromide. The resulting sulfides were then oxidised to the corresponding sulfones **2 a**–**c** using ruthenium(III) chloride and sodium periodate. Subsequently monofluorination by treatment with NFSI in the presence of ZnCl_2_ and LiHMDS^[21]^ furnished the desired α‐fluorinated sulfones **3 a** and **3 c**.

Similarly, the benzyl substituted analog **3 b** was prepared from 2‐mercaptobenzothiazole following a four‐step literature protocol^[22]^ (full details are provided in the ESI). For the purposes of comparison, the non‐fluorinated sulfoxides **4 a**–**c** were prepared by exposing **1 a**–**c** to standard *m*‐CPBA‐mediated oxidation conditions. Finally, the α‐fluorination of sulfides **1 a**–**c** was achieved via a fluoro‐Pummerer rearrangement sequence.

Initial *S*‐fluorination using Selectfluor^®^, α‐deprotonation, and subsequent attack of the liberated fluoride anion furnished the α‐fluorinated sulfides **5 a**–**c**. Regrettably, only α‐fluoro sulfide **5 b** was stable towards chromatographic purification and could be isolated and fully characterised. Derivatives **5 a/c** were hydrolytically unstable and thus were processed directly to the sulfoxide analogs with *m*‐CPBA. Whereas sulfoxides **6 a/b** (*anti/syn*) and **8 b** (*syn*) were isolated, the *anti* diastereoisomer **8 a** proved to be hydrolytically unstable towards column chromatography and recalcitrant towards recrystallisation.

### X‐ray crystallographic structure analysis

**Figure 2:**
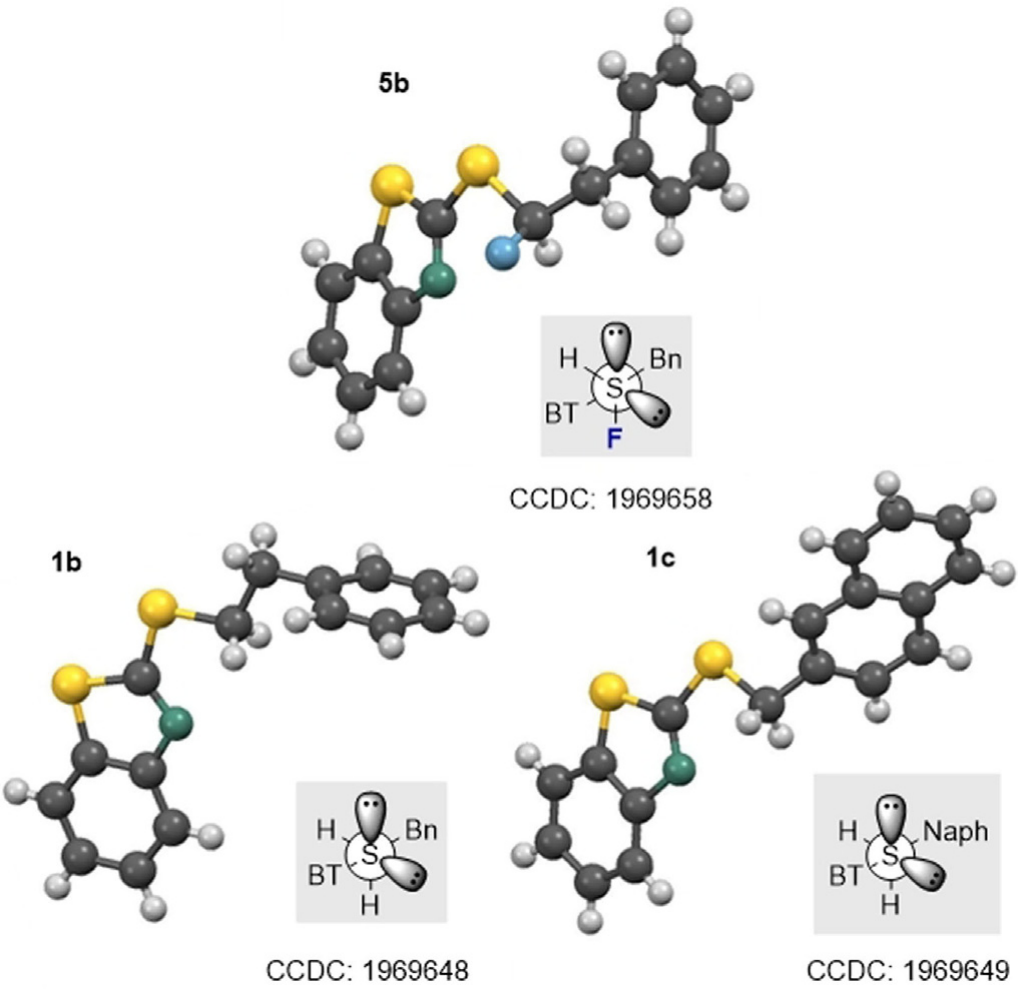
X‐ray crystallographic analyses of the α‐fluoro‐sulfide **5 b** (top, d_C‐F_=1.394(3) Å) and its non‐fluorinated analogs **1 b** (bottom left) and **1 c** (bottom right).

Single crystals suitable for X‐ray analysis were obtained for the α‐fluorinated sulfide **5 b**, sulfoxides **6 a/b**, **7 a/b** and **8 a**, and sulfones **3 a**–**c**. Analyses of the fluorinated sulfide **5 b** and the non‐fluorinated equivalents **1 b** and **1 c** (Figure 2) confirmed the expected distal alignment of the two largest substituents (Bn/Naph and BT), thereby minimising non‐bonding interactions. In the case of α‐fluorosulfide **5 b**, the C−F bond is orientated *anti* to one of the non‐bonding electron pairs in a conformation that is fully consistent with the anomeric effect [n_S_→σ_C‐F_*; d_C‐F_=1.394(3) Å].

**Figure 3:**
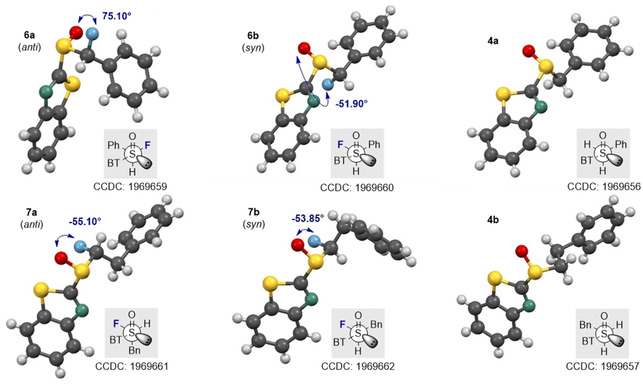
Crystallographic analyses of the α‐fluorinated sulfoxides **6 a**, **6 b**, **7 a**, **7 b** and their non‐fluorinated analogs **4 a** and **4 b**.

**Figure 4:**
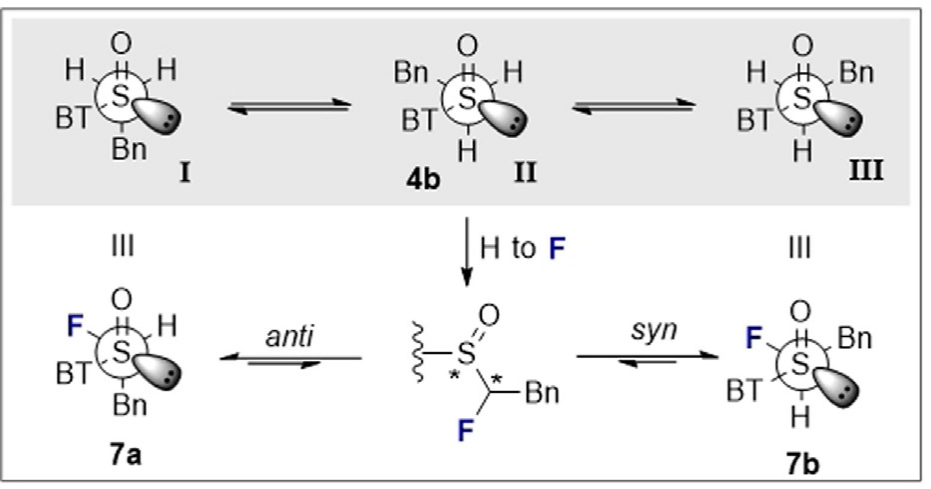
Observed conformations of the non‐fluorinated sulfoxide **4 b** (**II**) and its α‐fluorinated equivalents **7 a** and **7 b** in the solid state.

**Figure 5:**
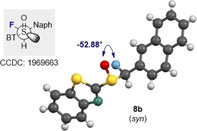
X‐ray crystal structure of the α‐fluorinated sulfoxide **8 b**.

In the case of the α‐fluorinated sulfoxides, conformations that manifest hyperconjugative effects are observed. In the X‐ray crystallographic structures of the fluorinated sulfoxides **6 a/b** and **7 a/b** (Figure 3), the carbon fluorine bonds are aligned *gauche* with respect to the dihedral angle φ_F‐C‐S‐O_. In all cases except for **6 a**, the carbon fluorine bonds are *antiperiplanar* to the non‐bonding electron lone pair on sulfur, in line with the anomeric effect. When comparing the X‐ray structure of the non‐fluorinated phenyl substituted derivative **4 a** with that of **6 b**, it appears as if H→F substitution in this *syn*‐diastereoisomer does not significantly impact the solid‐state conformation. The large aromatic substituents are distal in both structures as expected. However, in the *anti*‐diastereoisomer (**6 a** versus **6 b/4 a**), a difference in the solid‐state conformational preference is observed. In **6 a**, the benzothiazole and phenyl moieties are aligned in a counterintuitive *synclinal* orientation, which may be a consequence of packing effects in the solid state. In addition, the C−F bond is aligned between the non‐bonding electron pair and S=O bond of the sulfoxide. A longer C−F bond is observed in **6 a** than in **6 b** (1.391(4) Å and 1.373(6) Å, respectively. See Table 1). In the solid state, structure **6 a** also displays a weak C−H⋅⋅⋅F interaction with a hydrogen atom on an adjacent benzothiazole ring (C7‐H7⋅⋅⋅F1=2.590 Å. Please see the extended crystal packing in the ESI for full details). In contrast, the length of the carbon fluorine bonds in the diastereomeric scaffolds **7 a** and **7 b** (Bn versus Ph) are comparable in length (1.386(3) Å and 1.385(2) Å, respectively). This may be a consequence of stabilising hyperconjugative interactions of the type σ→σ_C‐F_* that are conspicuously absent in **6 b**. No evidence of π→σ_C‐F_* interactions was observed in this crystallographic analysis. A direct comparison of the benzyl substituted sulfoxides **4 b** (non‐fluorinated) with **7 a/b** (fluorinated) demonstrates that α‐fluorination has a notable impact on conformation. When considering the conformational equilibrium in Figure 4 (**I**, **II**, **III**), the non‐fluorinated system adopts conformation **II** in the solid state, whereas conformations **I** and **III** are observed for **7 a** and **7 b**, respectively. Crystallographic analysis of the fluorinated naphthalene substituted sulfoxide **8 b** is in line with the conformational analysis for **7 b** (Figure 5) in which the C−F bond is aligned *antiperiplanar* to the sulfur lone pair and *gauche* with respect to the S=O bond. This illustrates the utility of this motif in enabling acyclic conformational control.

**Table 1:** Selected crystallographic data for α‐fluorinated sulfide **5 b**, sulfoxides **6 a/b**, **7 a/b**, **8 a** and sulfones **3 a**–**c**.

Compound	φFCSO [°]	*d*C‐F [Å]	*d*S‐C(F) [Å]
**5 b**	–	1.394(3)	1.804(3)
**6 a**	−75.1(2)	1.391(4)	1.867(4)
**6 b**	−51.9(4)	1.373(6)	1.857(5)
**7 a**	−55.1(2)	1.386(3)	1.850(3)
**7 b**	53.8(1)	1.385(2)	1.840(2)
**8 b**	−52.9(2)	1.380(3)	1.869(2)
**3 a**	53.8(3)	1.367(4)	1.821(3)
**3 b**	59.1(2)	1.388(3)	1.812(2)
**3 c**	−58.5(4)	1.366(7)	1.832(6)

**Figure 6:**
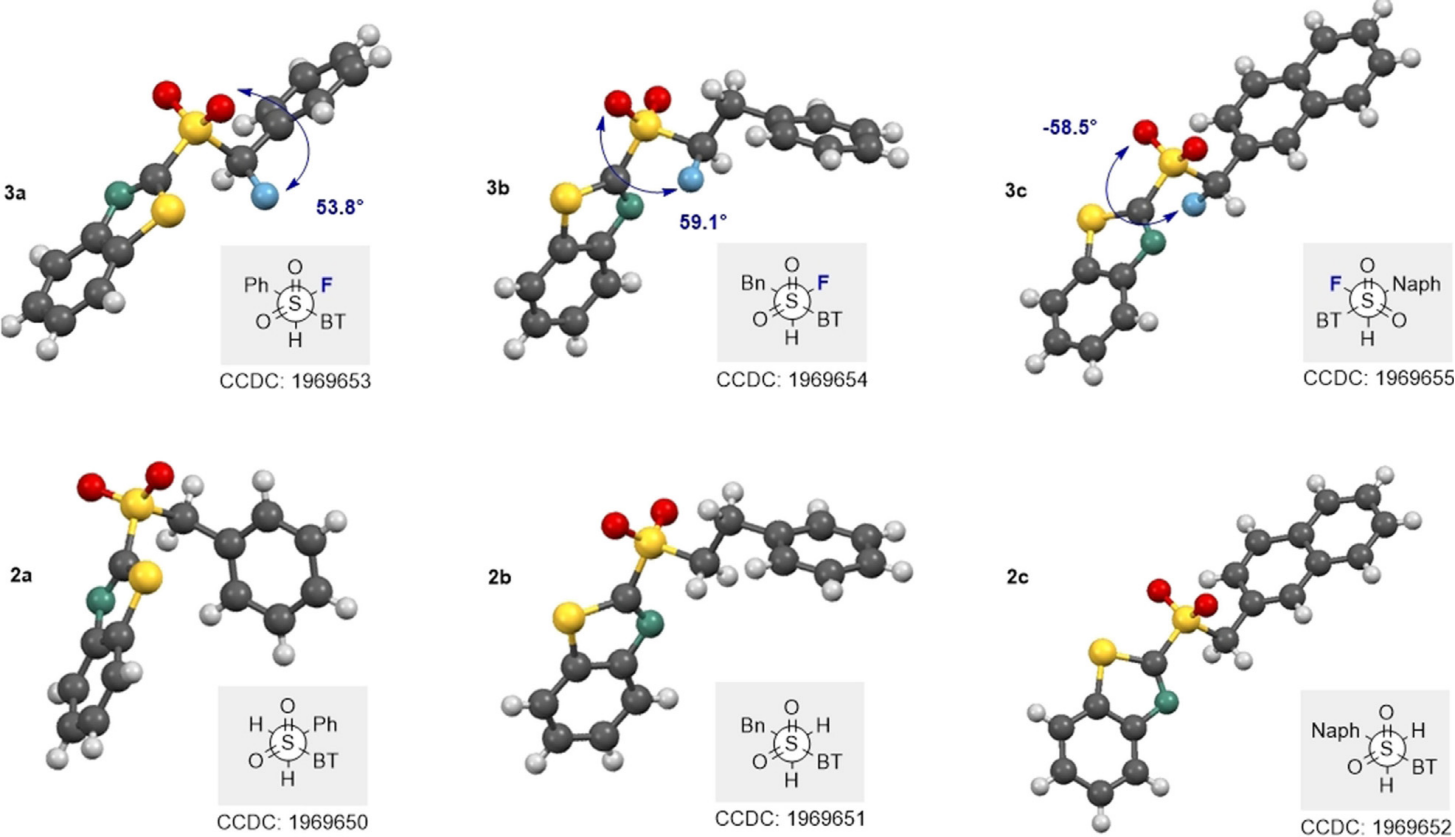
X‐ray crystallographic structures of α‐fluorinated sulfones **3 a**–**c** (top) and their non‐fluorinated equivalents **2 a**–**c** (bottom).

Solid state analyses of the fluorinated and non‐fluorinated sulfones (Figure 6) indicate a preference for an *antiperiplanar* alignment of the two aromatic substituents (Ph/Bn/Naph and BT), with the exception of the non‐fluorinated derivative **2 a**. Notably, the C−F distances in **3 a** and **3 c** are similar (1.367(4) Å and 1.366(7) Å, respectively), but slightly longer in **3 b** (1.388(3) Å). Furthermore, when comparing the mean lengths of the C−F bonds (Table 1) between the three sulfur oxidation states the following trend is observed: sulfide (1.390 Å) > sulfoxide (mean=1.383 Å) > sulfones (mean=1.374 Å).

The influence of fluorine insertion is also evident from inspection of the S(O)_*n*_−C(sp^3^) bond length in the fluorinated compounds, as compared to the non‐fluorinated analogs (Table 1). In general, *d*S‐C(F) > *d*S‐C(H) which may be of note considering the significance of fractional bonds in understanding the dependence of small changes in ground state structure on reactivity.^[23]^

### Computational study of conformers in solution

To complement the solid‐state analysis, and compensate for the absence of ^3^ *J*_HF_ coupling constants that would facilitate a detailed NMR conformer population analysis, a computational investigation was conducted. This would allow the dominant non‐covalent interactions that are operational in solution to be delineated and allow a direct comparison with the crystallographic analyses.

**Figure 7:**
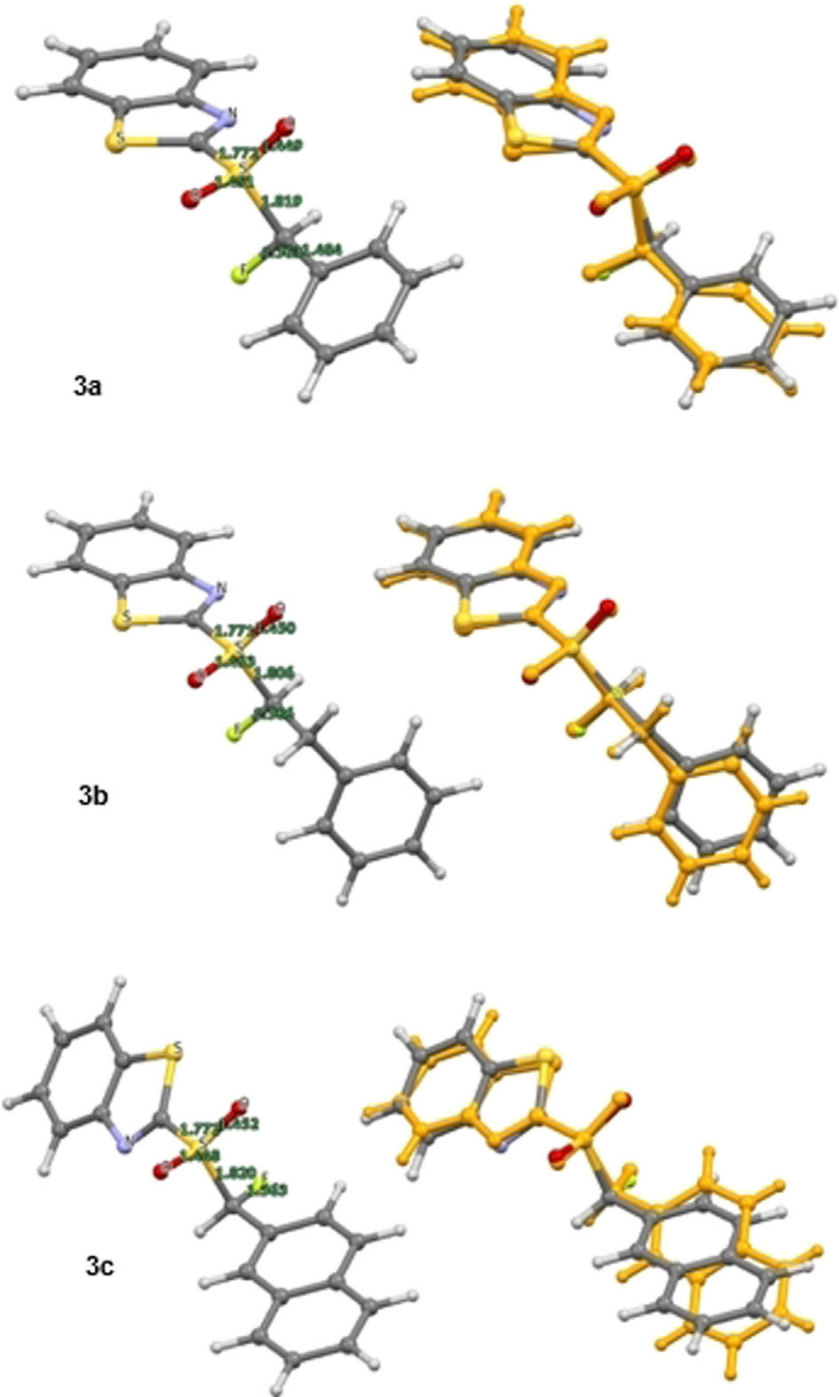
Molecular structure of sulfones **3 a**–**c**. DFT‐optimised conformations (left) and overlay of the conformer representing the solid‐state structure with the experimental structure (orange).

Initially, the sulfone series was evaluated at the DFT level of theory (Figure 7). In general, this analysis revealed several conformations within a very narrow free energy range (<1 kcal mol^−1^). For all three fluorinated sulfones (**3 a**–**c**), there was good agreement between the calculated structure and the crystallographic analysis. In all cases, preferred conformers were identified in which the substituent (Ph, Bn and Naph) was *anti* to the benzothiazole unit.

**Figure 8:**
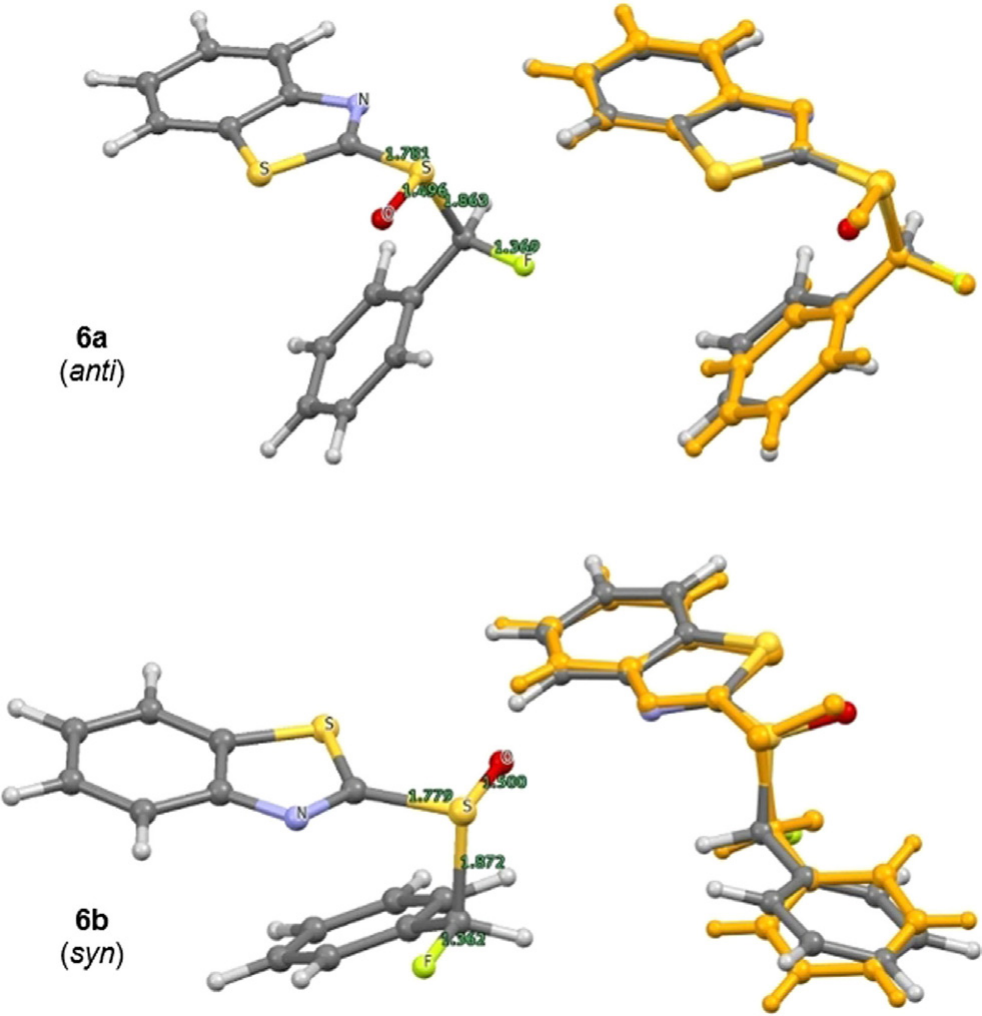
Sulfoxides **6 a/b**. DFT‐optimised conformations (left) and overlay of the conformer representing the solid‐state structure with the experimental structure (orange).

**Figure 9:**
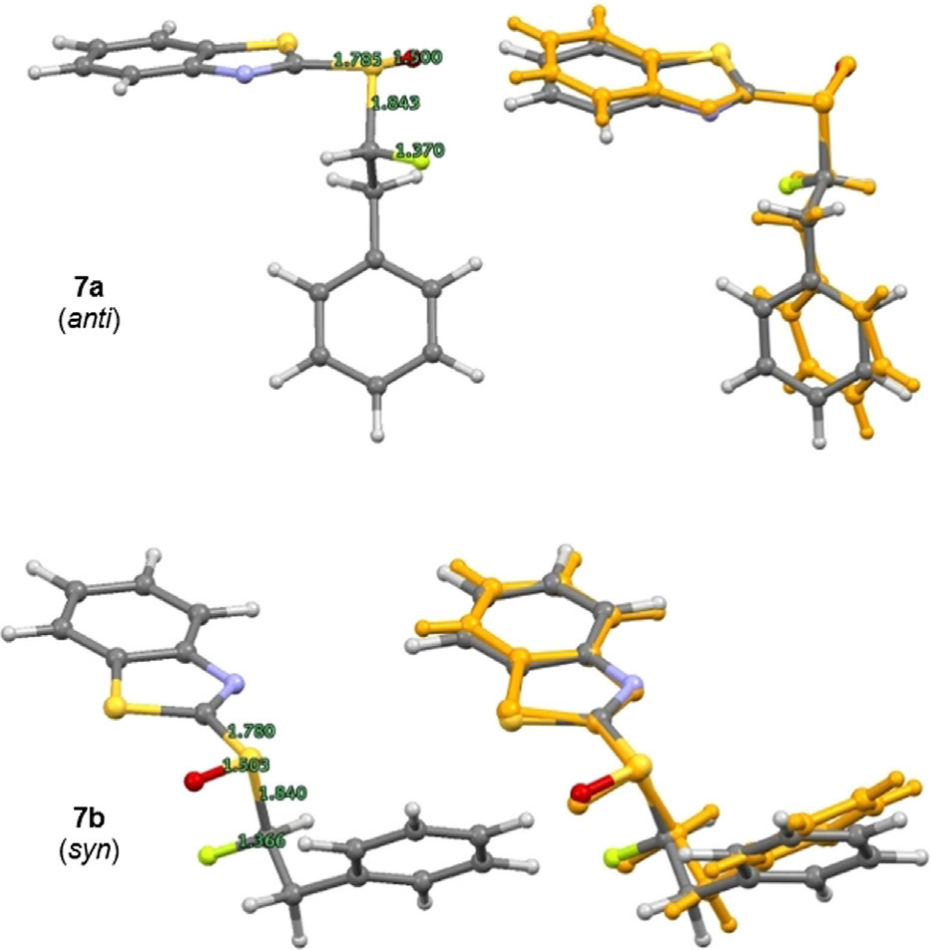
Structure of sulfoxides **7 a/b**. DFT‐optimised conformations (left) and overlay of the conformer representing the solid‐state structure with the experimental structure (orange).

**Figure 10:**
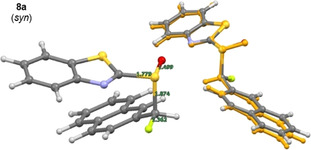
Molecular structure of **8 a**. DFT‐optimised conformation (left) and overlay of the conformer representing the solid‐state structure with the experimental structure (orange).

In the sulfoxide series, divergence between the computed solution conformation and the solid‐state structure for several of the diastereoisomers examined (**6 b**, **7 a** and **8 a**, see Table 2). Whereas the predicted conformation of the phenyl substituted sulfoxide **6 a** is in line with the crystal structure analysis, the computationally predicted structure of **6 b** adopts a conformation in which the C−F and S=O bonds are aligned *anti* to one another (Figure 8). This contrasts starkly with the crystallographic data, in which a conformation that allows for a hyperconjugative n_S_→σ_C‐F_* interaction manifests itself (Figure 3). In the crystal structures of **7 a** and **8 a**, a similar orientation of the carbon fluorine bond is observed. In both cases the predicted conformer is preferred by 1.6 kcal mol^−1^ (see Table 2). The calculated structure of **7 a** (Figure 9) has the C−F bond partitioned between the sulfur lone pair and the S=O bond, similar to the experimentally observed and computationally predicted structure of its phenyl substituted equivalent **6 a** (Figure 3). Whereas the predicted conformer of **8 a** (Figure 10) is analogous to that calculated for **6 b**, in which the two C−F and S=O dipoles are minimised by virtue of their *anti*‐alignment. Finally, the predicted structure of the *syn* substituted phenyl derivative **7 b** matches the conformation determined by X‐ray analysis (Figure 9).

**Table 2:** Selected crystallographic data for α‐fluorinated sulfoxides **6 a/b**, **7 a/b** and **8 a** and their predicted lowest energy conformers in CHCl_3_. Δ*G*=free energy difference of the solid‐state conformer (left) with respect to the predicted low energy structure (right).

Compound	Experimental (solid state)	Predicted (solution)	Δ*G* (kcal/mol)
**6 a** (*anti*)	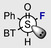	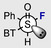	0
**6 b** (*syn*)	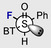	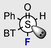	+0.7
**7 a** (*anti*)	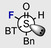	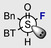	+1.6
**7 b** (*syn*)	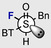	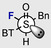	0
**8 a** (*syn*)	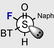	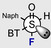	+1.6

**Figure 11:**
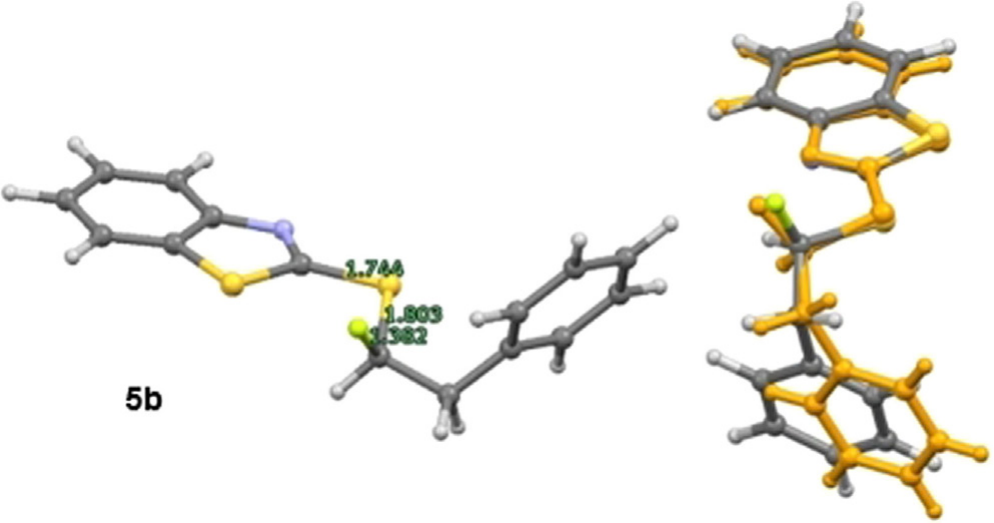
Structure of fluorinated sulfide **5 b**. DFT‐optimised conformation (left) and overlay of the conformer representing the solid‐state structure with the experimental structure (orange).

Theory predicts at least six conformations of fluorinated sulfide **5 b** with relative free energies <1 kcal mol^−1^. The X‐ray structure only correlates with one of these that is predicted to be +0.7 kcal mol^−1^ high in energy than the most stable conformer (see Figure 11).

**Figure 12:**
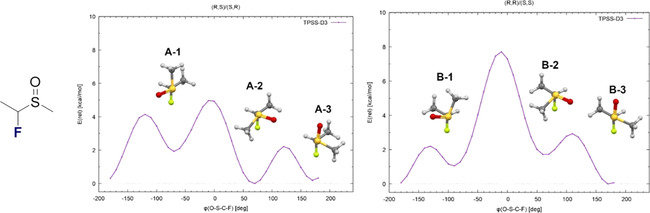
Energy profile of S‐C(F) bond rotation in the *syn* (A, left) and *anti* (B, right) diastereoisomers of methyl‐(1‐fluoroethyl)‐sulfoxide.

To further explore the acyclic conformational behaviour of α‐fluoro sulfoxides, a simplified model compound was conceived to establish bond rotational profiles of the two diastereiosiosmers (*syn* and *anti*) [diastereoisomers **A** (*syn*) and **B** (*anti*) of methyl‐(1‐fluoroethyl)sulfoxide, Figure 12]. The O‐S‐C‐F dihedral angle (φ) energy profile was calculated at the TPSS‐D3/def2‐TZVP level of theory, optimising all other structural parameters on each point of the profile. For each diastereoisomer, three energetic minima were distinguished that mitigate unfavourable interactions involving the polarised S=O and C−F bonds (Figure 12). The (*syn*) diastereoisomer **A** has two low energy conformers in which either destabilising non‐bonding interactions between the methyl substituents are avoided (**A‐2**, *φ*=69°) or in which the polar bonds adopt an *anti*‐orientation (**A‐3**, *φ*=172°). For the *anti*‐diastereoisomer **B**, both of these conditions are satisfied in conformer **B‐3** (*φ*=174°). Analysis of **A‐2** and **B‐2** reveals the presence of the suspected n_S_→σ_C‐F_* hyperconjugative interaction.^[24]^

Although intuitive and didactically helpful in predicting the conformation of α‐fluoro sulfoxides, this interaction, does not contribute significantly to the stabilisation of the molecule as revealed by the second order perturbation theory energies (E_2_^PT^). Although the hyperconjugative interaction n_S_→σ^*^_C‐F_ contributes >3 kcal mol^−1^ to the total stabilisation, other interactions are more dominant. Pertinent examples include the n_O_→σ^*^_S‐C(F)_ (E_2_^PT^≈11 kcal mol^−1^) and n_F_→σ^*^_S‐C(F)_ (E_2_^PT^≈4 kcal mol^−1^) interactions.

**Figure 13:**
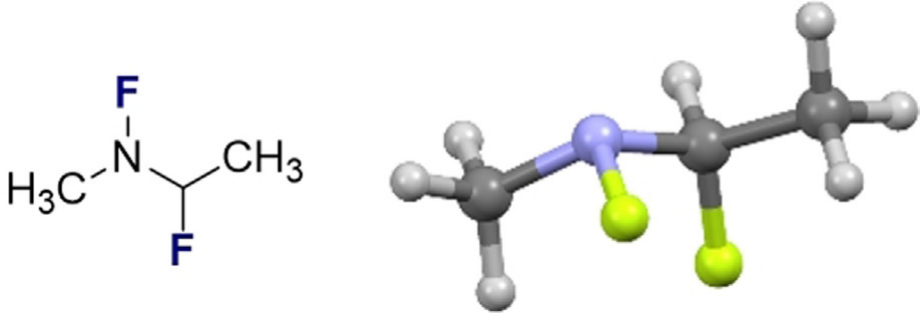
Model compound used for comparing the n_S_→σ^*^_C‐F_ hyperconjugation with an n_n_→σ^*^_C‐F_ interaction.

Replacement of the S=YO unit by *N*‐F as shown in Figure 13, reinstates the anomeric effect as a defining conformational factor in these closely related systems, due to the less diffuse character of nitrogen electron pairs. Herein, hyperconjugative stabilisation of E_2_^PT^=11.2 kcal mol^−1^ for the n_n_→σ^*^_C‐F_ interaction is calculated. This final comparison serves to underscore the modular nature of hyperconjugative interactions in enabling acyclic conformational control. This is fully consistent with the stereoelectronic analysis of the anomeric effect in fluoromethylamine delineated by Dunitz.^[8]^

## Conclusions

Motivated by the increasing popularity of the RS(O)_n_CHFR motif in bioactive small molecule discovery, a combined experimental and computational analysis of α‐fluoro‐sulfides, ‐sulfoxides and ‐sulfones is disclosed. Conformations that are intuitively preferred by stereoelectronic theory generally prevail as demonstrated through the inclusion of several X‐ray analyses. Given the prevalence of S=O and C−F based interactions that are independently prevalent in small molecule drug discovery,[^25^, ^26^] it is envisaged that this analysis of the RS(O)_n_CHFR motif will find application in focused molecular design.

## Experimental Section

General Information: All commercially available chemicals were purchased as reagent grade and were used directly as received. Solvents used for extractions and flash chromatography were of technical grade and distilled prior to use. Where indicated, dry solvents were dried through a Grubbs purification system. Pre‐coated silica gel 60 F254 aluminium plates from Merck were used for thin layer chromatography (TLC) and were visualised under UV‐light and/or stained with a solution of KMnO4. Fluka silica gel (230–400 mesh) was employed for purification with flash chromatography. NMR measurements were performed by the NMR service of the Organic Chemistry institute (WWU, Münster) on a Bruker AV300, AV400 or an Agilent DD2 600 at the indicated temperature. Additional 1D and 2D NMR experiments (e.g., 1H{19F}, 19F{1H}, DEPT, COSY, HMBC and HSQC) were used to assign the resonances of previously unreported compounds. Chemical shifts (δ, ppm) are referenced to esidual solvent signals and the coupling constants (*J*) are given in Hz. The resonance multiplicities are declared as: s (singlet), d (doublet), t (triplet), q (quartet) and m (multiplet). High resolution mass spectra (HR‐ESI) were recorded by the MS service of the Organic Chemistry department (WWU, Münster). Melting points were assessed in oPentane capillaries on a Büchi B‐540 apparatus. IR spectra were measured on a PerkinElmer 100 FT‐IR (ATR) spectrometer. The adsorption bands are reported in wavenumbers (cm^−1^) and intensities are abbreviated and described as: w (weak), m (medium) or s (strong).

**General Procedure 1**: To a flame dried Schlenk flask containing 2‐mercaptobenzothiazole (1.00 equiv.) and DMF (dried under 4 Å MS) was added NaH (60 % in mineral oil, 1.30 equiv.) at 0 °C. The mixture was stirred at 0 °C for 30 min where after the brominated electrophile (1.10 equiv.) was added. The reaction mixture was stirred under an argon atmosphere at room temperature overnight. The mixture was then diluted with water, extracted with EtOAc (*x* 3) and the combined organic layers were dried (Na_2_SO_4_) and concentrated in vacuo.

**General Procedure 2**: To a solution of the designated thioether (1.00 equiv.) in DCM was added *m*‐chloroperoxybenzoic acid (1.00 equiv.) at 0 °C. The resulting mixture was stirred at room temperature for 24–26 h where after the solvent was removed under reduced pressure to afford the crude product.

**General Procedure 3**: The designated thioether (1.00 equiv.) was dissolved in a mixture of CHCl_3_/ MeCN/ H_2_O (1:1:2.6) and NaIO_4_ (4.00 equiv.) and ruthenium (III) chloride hydrate (2 mg) was added. The mixture was stirred at room temperature overnight and then filtered over Celite^®^. The organic solvents were removed in vacuo and the residue was extracted with DCM (*x* 3). The combined organic layers were washed (brine), dried (MgSO_4_) and concentrated under reduced pressure to obtain the crude product.

**General Procedure 4**: A flame dried Schlenk flask was charged with Selectfluor^®^ (1.25 equiv.) and ACN. The designated thioether (1.00 equiv. in ACN) was then added dropwise to the suspension and the mixture was stirred under argon at room temperature for 10 min where after Et_3_N (1.25 equiv.) was added. The reaction mixture was stirred for another 10 min at room temperature before it was poured into water, extracted with DCM (*x* 3), dried (Na_2_SO_4_) and concentrated in vacuo. The residue was dissolved in DCM and *m*‐chloroperoxybenzoic acid (1.00 equiv.) was added at 0 °C. The reaction mixture was then stirred at room temperature for 17–45 h where after a saturated aq. solution of Na_2_SO_3_ was added and the mixture was stirred at room temperature for another 2 h. The organic layer was then washed with saturated aq. NaHCO_3_, dried (Na_2_SO_4_), filtered and concentrated under reduced pressure.

**General Procedure 5**: Based on a modified literature procedure.^[21]^ ZnCl_2_ (3.50 equiv.) was flame dried in a Schlenk flask and dry THF was added. LiHMDS (2.20 equiv.) was then added and the mixture was stirred at room temperature for 5 min where after the designated sulfone (1.00 equiv.) was added. After stirring for 30 min NFSI (2.00 equiv.) was added and the resulting reaction mixture was then stirred at room temperature for 6–7 h. The reaction was set‐up and stirred under an argon atmosphere. A solution of aq. HCl (1 m) was then added and the mixture was extracted with EtOAc (*x* 3). The combined organic layers were dried (MgSO4) and concentrated in vacuo to obtain the crude mixture.

**2‐(Benzylthio)benzo[d]thiazole (1 a)**: The product was synthesised from benzyl bromide (0.77 mL, 6.50 mmol, 1.10 equiv.) according to General Procedure 1. The product was purified via flash chromatography (CyH:EtOAc/ 50:1) and was afforded as a light yellow solid (1.522 g, 99 %). M.p.: 35 °C; *R*_f_ 0.33 (CyH:EtOAc/ 20:1); HRMS *m*/*z* (ESI): Calcd. for C_14_H_11_NS_2_Na^+^ 280.0225, found 280.0229; ^1^H NMR (600 MHz, Methylene chloride‐*d_2_*, 298 K) *δ* 7.89 (ddd, ^3^ *J*HH=8.2 Hz, ^4^ *J*HH=1.2 Hz, ^5^ *J*HH=0.6 Hz, 1 H, HC4), 7.79 (ddd, ^3^ *J*HH=8.0 Hz, ^4^ *J*HH=1.2 Hz, ^5^ *J*HH=0.6 Hz, 1 H, HC7), 7.50–7.47 (m, 2 H, HC10), 7.46–7.43 (m, 1 H, HC5), 7.36–7.28 (m, 4 H, HC6,11,12), 4.62 (s, 2 H, H_2_C8); ^13^C{^1^H} NMR (151 MHz, Methylene chloride‐*d_2_*, 298 K) *δ* 166.9 (C1), 153.8 (C3), 137.2 (C9), 136.0 (C2), 129.7 (C10), 129.2 (C11), 128.2 (C12), 126.6 (C5), 124.8 (C6), 122.0 (C4), 121.6 (C7), 38.1 (C8); IR (cm^−1^): 3059 (w), 3030 (w), 1495 (m), 1454 (s), 1424 (s), 1310 (m), 1275 (m), 1239 (m), 1192 (m), 1124 (m), 1071 (m), 1019 (m), 996 (s), 934 (m), 916 (m), 860 (m), 814 (m), 768 (m), 748 (s), 725 (s), 709 (s), 691 (s), 675 (s).

**2‐(Phenethylthio)benzo[d]thiazole (1 b)**: The product was synthesised from (2‐bromoethyl)benzene (450 μL, 3.29 mmol, 1.10 equiv.) according to General Procedure 1. The product was purified by flash chromatography (CyH:EtOAc/ 30:1) and was obtained as a colourless oil (789 mg, 97 %). *R*_f_ 0.21 (CyH:EtOAc/ 30:1); HRMS *m*/*z* (ESI): Calcd. for C_15_H_13_NS_2_Na^+^ 294.0387, found 294.0384; ^1^H NMR (600 MHz, Methylene chloride‐*d_2_*, 298 K) *δ* 7.88 (ddt, ^3^ *J*HH=8.1 Hz, ^4^ *J*HH=1.2 Hz, ^5^ *J*HH=0.6 Hz, 1 H, HC4), 7.80 (ddt, ^3^ *J*HH=8.0 Hz, ^4^ *J*HH=1.2 Hz, ^5^ *J*HH=0.6 Hz, 1 H, HC7), 7.44 (m, 1 H, HC5), 7.36–7.29 (m, 5 H, HC6,11,12), 7.26 (m, 1 H, HC13), 3.61 (m, 2 H, H_2_C8), 3.15 (m, 2 H, H_2_C9); ^13^C{^1^H} NMR (151 MHz, Methylene chloride‐*d_2_*, 298 K) *δ* 167.2 (C1), 154.0 (C3), 140.4 (C10), 135.9 (C2), 129.2 (C11), 129.1 (C12), 127.2 (C13), 126.6 (C5), 124.7 (C6), 122.0 (C4), 121.6 (C7), 36.1 (C9), 35.3 (C8); IR (cm^−1^): 3060 (w), 3026 (w), 2923 (w), 2853 (w), 1603 (w), 1560 (w), 1496 (w), 1455 (m), 1425 (s), 1309 (m), 1274 (w), 1238 (m), 1177 (w), 1157 (w), 1126 (w), 1075 (w), 1018 (m), 992 (s), 934 (w), 880 (w), 851 (w), 826 (w), 752 (s), 724 (m), 712 (m), 695 (s), 668 (m).

**2‐((Naphthalen‐2‐ylmethyl)thio)benzo[d]thiazole (1 c)**: The compound was prepared from 2‐(bromomethyl)naphthalene (3.62 g, 16.4 mmol, 1.10 equiv.) according to General Procedure 1. The crude mixture was adsorbed on silica and purified by flash chromatography (CyH:EtOAc/ 60:1), which afforded the product as a white solid (3.16 g, 69 %). M.p.: 78 °C; *R*_f_ 0.30 (CyH:EtOAc/ 19:1); HRMS *m*/*z* (ESI): Calcd. for C_18_H_13_NS_2_Na^+^ 330.0387, found 330.0386; ^1^H NMR (500 MHz, Methylene chloride‐*d_2_*, 298 K) *δ* 7.94 (dh, ^4^ *J*HH=2.0 Hz, ^5^ *J*HH=0.7 Hz, 1 H, HC14), 7.90 (ddd, ^3^ *J*HH=8.2 Hz, ^4^ *J*HH=1.2 Hz, ^5^ *J*HH=0.6 Hz, 1 H, HC4), 7.85–7.81 (m, 3 H, HC11,15,18), 7.78 (ddd, ^3^ *J*HH=8.0 Hz, ^4^ *J*HH=1.3 Hz, ^5^ *J*HH=0.6 Hz, 1 H, HC7), 7.59 (dd, ^3^ *J*HH=8.5 Hz, ^4^ *J*HH=1.8 Hz, 1 H, HC10), 7.48 (m, 2 H, HC16,17), 7.44 (ddd, ^3^ *J*HH=8.2, 7.3 Hz, ^4^ *J*HH=1.3 Hz, 1 H, HC5), 7.31 (ddd, ^3^ *J*HH=8.0, 7.3 Hz, ^4^ *J*HH=1.2 Hz, 1 H, HC6), 4.78 (s, 2 H, H_2_C8); ^13^C{^1^H} NMR (126 MHz, Methylene chloride‐*d_2_*, 298 K) *δ* 166.8 (C1), 153.8 (C3), 136.0 (C2), 134.6 (C9), 133.9 (C13), 133.4 (C12), 129.0 (C11), 128.5 (C14), 128.3 (C18), 128.2 (C15), 127.5 (C10), [126.9, 126.7](C16,17), 126.6 (C5), 124.9 (C6), 122.0 (C4), 121.7 (C7), 38.4 (C8); IR (cm^−1^): 3051 (w), 2926 (w), 1597 (w), 1508 (w), 1454 (m), 1424 (s), 1361 (w), 1310 (m), 1275 (w), 1240 (w), 1203 (w), 1122 (m), 1074 (m), 1017 (m), 1000 (m), 963 (m), 950 (m), 935 (m), 898 (m), 874 (w), 854 (m), 817 (m), 772 (w), 748 (s), 725 (s), 703 (m), 668 (m).

**2‐(Benzylsulfonyl)benzo[d]thiazole (2 a)**: The product was synthesised from **1 a** (200 mg, 0.78 mmol, 1.00 equiv.) according to General Procedure 3. The product was purified by recrystallisation of the crude mixture (DCM:CyH) and was afforded as a white solid (156 mg, 69 %). M.p.: 108 °C; *R*_f_ 0.22 (CyH:EtOAc/ 4:1); HRMS *m*/*z* (ESI): Calcd. for C_14_H_11_NO_2_S_2_Na^+^ 312.0129, found 312.0123; ^1^H NMR (400 MHz, Methylene chloride‐*d_2_*, 298 K) *δ* 10.20 (ddd, ^3^ *J*HH=8.3 Hz, ^4^ *J*HH=1.2 Hz, ^5^ *J*HH=0.7 Hz, 1 H, HC4), 9.93 (ddd, ^3^ *J*HH=8.0 Hz, ^4^ *J*HH=1.2 Hz, ^5^ *J*HH=0.7 Hz, 1 H, HC7), 9.62 (ddd, ^3^ *J*HH=8.3, 7.2 Hz, ^4^ *J*HH=1.3 Hz, 1 H, HC5), 9.55 (ddd, ^3^ *J*HH=8.3, 7.2 Hz, ^4^ *J*HH=1.3 Hz, 1 H, HC6), 9.32–9.19 (m, 5 H, HC10,11,12), 6.69 (s, 2 H, H_2_C8); ^13^C{^1^H} NMR (101 MHz, Methylene chloride‐*d_2_*, 298 K) *δ* 165.8 (C1), 153.3 (C3), 137.6 (C2), 131.7 (C10), 129.7 (C12), 129.4 (C11), 128.6 (C6), 128.2 (C5), 127.2 (C9), 125.9 (C4), 122.9 (C7), 61.5 (C8); IR (cm^−1^): 2978 (w), 2920 (w), 1554 (w), 1496 (w), 1478 (w), 1456 (w), 1421 (w), 1405 (w), 1333 (m), 1315 (w), 1276 (w), 1252 (w), 1198 (w), 1150 (m), 1128 (m), 1081 (w), 1073 (w), 1024 (w), 940 (w), 927 (w), 882 (w), 851 (w), 779 (m), 760 (m), 730 (m), 697 (m), 687 (m).

**2‐(Phenethylsulfonyl)benzo[d]thiazole (2 b)**: The compound was synthesised from **1 b** (200 mg, 0.74 mmol, 1.00 equiv.) according to General Procedure 3. The product was purified by flash chromatography (DCM) and was afforded as a white solid (118 mg, 53 %). M.p.: 84 °C; *R*_f_ 0.63 (DCM); HRMS *m*/*z* (ESI): Calcd. for C_15_H_13_NO_2_S_2_Na^+^ 326.0280, found 326.0288; ^1^H NMR (600 MHz, Methylene chloride‐*d_2_*, 298 K) *δ* 8.22 (ddd, ^3^ *J*HH=8.3 Hz, ^4^ *J*HH=1.3 Hz, ^5^ *J*HH=0.7 Hz, 1 H, HC4), 8.05 (ddd, ^3^ *J*HH=8.1 Hz, ^4^ *J*HH=1.3 Hz, ^5^ *J*HH=0.7 Hz, 1 H, HC7), 7.67 (ddd, ^3^ *J*HH=8.3, 7.2 Hz, ^4^ *J*HH=1.3 Hz, 1 H, HC5), 7.62 (ddd, ^3^ *J*HH=8.3, 7.2 Hz, ^4^ *J*HH=1.3 Hz, 1 H, HC6), 7.28–7.23 (m, 2 H, HC12), 7.20–7.17 (m, 3 H, HC11,13), 3.80 (m, 2 H, H_2_C8), 3.19 (m, 2 H, H_2_C9); ^13^C{^1^H} NMR (151 MHz, Methylene chloride‐*d_2_*, 298 K) *δ* 166.3 (C1), 153.4 (C3), 137.7 (C10), 137.4 (C2), 129.3 (C12), 129.0 (C11), 128.6 (C6), 128.2 (C5), 127.5 (C13), 125.9 (C4), 123.0 (C7), 56.4 (C8), 29.1 (C9); IR (cm^−1^): 2930 (w), 1601 (w), 1552 (w), 1496 (w), 1469 (m), 1455 (m), 1422 (w), 1403 (w), 1327 (m), 1312 (m), 1260 (w), 1236 (w), 1203 (w), 1165 (w), 1146 (s), 1127 (m), 1084 (m), 1072 (w), 1025 (m), 1005 (m), 965 (w), 907 (w), 852 (m), 773 (m), 762 (s), 741 (m), 728 (m), 696 (s).

**2‐((Naphthalen‐2‐ylmethyl)sulfonyl)benzo[d]thiazole (2 c)**: The compound was prepared from **1 c** (140 mg, 0.46 mmol, 1.00 equiv.) following General Procedure 3. The crude mixture was purified by flash chromatography (CyH:EtOAc/ 12:1) which afforded the product as a light yellow solid (110 mg, 71 %). M.p.: 132 °C; *R*_f_ 0.28 (CyH:EtOAc/ 6:1); HRMS *m*/*z* (ESI): Calcd. for C_18_H_13_NO_2_S_2_Na^+^ 362.0285, found 362.0280; ^1^H NMR (400 MHz, Methylene chloride‐*d_2_*, 298 K) *δ* 8.28 (ddd, ^3^ *J*HH=8.3 Hz, ^4^ *J*HH=1.2 Hz, ^5^ *J*HH=0.7 Hz, 1 H, HC4), 7.95 (ddd, ^3^ *J*HH=8.1 Hz, ^4^ *J*HH=1.3 Hz, ^5^ *J*HH=0.7 Hz, 1 H, HC7), 7.82 (m, 1 H, HC15), 7.78 (d, ^3^ *J*HH=8.4 Hz, 1 H, HC11), 7.77 (s, 1 H, HC14), 7.73 (m, 1 H, HC18), 7.68 (ddd, ^3^ *J*HH=8.3, 7.2 Hz, ^4^ *J*HH=1.3 Hz, 1 H, HC5), 7.59 (ddd, ^3^ *J*HH=8.3, 7.2 Hz, ^4^ *J*HH=1.2 Hz, 1 H, HC6), 7.49 (m, 2 H, HC16,17), 7.36 (dd, ^3^ *J*HH=8.4 HZ, ^4^ *J*HH=1.9 Hz, 1 H, HC10), 4.92 (s, 2 H, H_2_C8); ^13^C{^1^H} NMR (101 MHz, Methylene chloride‐*d_2_*, 298 K) *δ* 165.9 (C1), 153.3 (C3), 137.6 (C2), 133.8 (C12), 133.7 (C13), 131.7 (C14), 129.1 (C11), 128.6 (C6), 128.5 (C18), 128.4 (C10), 128.3 (C5), 128.2 (C15), 127.5 (C16), 127.2 (C17), 125.9 (C4), 124.6 (C9), 123.0 (C7), 61.7 (C8); IR (cm^−1^): 2925 (w), 1598 (w), 1507 (w), 1473 (m), 1457 (w), 1425 (w), 1406 (w), 1364 (w), 1314 (s), 1276 (m), 1254 (w), 1233 (m), 1210 (w), 1162 (w), 1140 (s), 1124 (m), 1086 (w), 1023 (m), 969 (w), 957 (w), 949 (w), 922 (w), 902 (m), 872 (w), 864 (w), 851 (m), 828 (m), 764 (s), 753 (s), 725 (s), 707 (m), 683 (w).

**2‐((Fluoro(phenyl)methyl)sulfonyl)benzo[d]thiazole (3 a)**: The product was synthesised from **2 a** (243 mg, 0.84 mmol, 1.00 equiv.) following General Procedure 5 (reaction time: 7 h). The crude product was adsorbed to silica and purified by flash chromatography (CyH:EtOAc/ 10:1), which afforded the product as a white solid (188 mg, 73 %). M.p.: 141 °C; *R*_f_ 0.30 (CyH:EtOAc/ 8:1); HRMS *m*/*z* (ESI): Calcd. for C_14_H_10_FNO_2_S_2_Na^+^ 330.0035, found 330.0034; ^1^H NMR (600 MHz, Methylene chloride‐*d_2_*, 298 K) *δ* 8.29 (ddd, ^3^ *J*HH=8.3 Hz, ^4^ *J*HH=1.2 Hz, ^5^ *J*HH=0.7 Hz, 1 H, HC4), 8.08 (ddd, ^3^ *J*HH=8.1 Hz, ^4^ *J*HH=1.4 Hz, ^5^ *J*HH=0.8 Hz, 1 H, HC7), 7.70 (ddd, ^3^ *J*HH=8.3, 7.2 Hz, ^4^ *J*HH=1.3 Hz, 1 H, HC5), 7.66 (ddd, ^3^ *J*HH=8.3, 7.2 Hz, ^4^ *J*HH=1.3 Hz, 1 H, HC6), 7.61 (m, 2 H, HC10), 7.57 (m, 1 H, HC12), 7.51 (m, 2 H, HC11), 6.65 (d, ^2^ *J*FH=45.7 Hz, 1 H, HC8); ^13^C{^1^H} NMR (151 MHz, Methylene chloride‐*d_2_*, 298 K) *δ* 162.6 (C1), 152.9 (C3), 137.5 (C2), 131.6 (d, ^5^ *J*FC=1.5 Hz, C12), 128.8 (C11), 128.4 (C6), 128.3 (d, ^3^ *J*FC=6.3 Hz, C10), 127.9 (C5), 126.5 (d, ^2^ *J*FC=19.4 Hz, C9), 125.6 (C4), 122.4 (C7), 102.0 (d, ^1^ *J*FC=222.5 Hz, C8); ^19^F NMR (564 MHz, Methylene chloride‐*d_2_*, 298 K) δ−173.1 (d, ^2^ *J*FH=45.7 Hz, 1F, FC8); IR (cm^−1^): 2968 (w), 1494 (w), 1460 (m), 1413 (w), 1347 (s), 1316 (m), 1294 (w), 1236 (w), 1197 (w), 1156 (m), 1083 (m), 1040 (m), 1021 (m), 984 (w), 854 (w), 791 (w), 760 (s), 734 (m), 709 (w), 696 (s).

**2‐((1‐Fluoro‐2‐phenylethyl)sulfonyl)benzo[d]thiazole (3 b)**: Synthesised according to following literature procedure.^22 1^H NMR (600 MHz, Methylene chloride‐*d_2_*, 298 K) *δ* 8.26 (ddd, ^3^ *J*HH=8.2 Hz, ^4^ *J*HH=1.5 Hz, ^5^ *J*HH=0.7 Hz, 1 H, HC4), 8.08 (ddd, ^3^ *J*HH=7.9 Hz, ^4^ *J*HH=1.5 Hz, ^5^ *J*HH=0.7 Hz, 1 H, HC7), 7.67 (m, 2 H, HC5,6), 7.35 (m, 2 H, HC12), 7.33–7.29 (m, 3 H, HC11,13), 5.83 (ddd, ^2^ *J*FH=48.2 Hz, ^3^ *J*HH=10.3, 2.5 Hz, 1 H, HC8), [3.63 (ddd, ^3^ *J*FH=38.9 Hz, ^2^ *J*HH=15.0 Hz, ^3^ *J*HH=2.5 Hz, 1 H), 3.39 (ddd, ^3^ *J*FH=16.3 Hz, ^2^ *J*HH=15.0 Hz, ^3^ *J*HH=10.4 Hz, 1 H)](H_2_C9); ^13^C{^1^H} NMR (151 MHz, Methylene chloride‐*d_2_*, 298 K) *δ* 162.8 (C1), 153.5 (C3), 138.1 (C2), 133.7 (C10), 130.1 (C11), 129.5 (C12), 129.1 (C6), 128.5 (C5), 128.3 (C13), 126.2 (C4), 123.0 (C7), 102.9 (d, ^1^ *J*FC=223.7 Hz, C8), 33.9 (d, ^2^ *J*FC=19.4 Hz, C9); ^19^F NMR (564 MHz, Methylene chloride‐*d_2_*, 298 K) δ−177.7 (ddd, ^2^ *J*FH=48.2 Hz, ^3^ *J*FH=39.0, 16.4 Hz, 1F, FC8).

**2‐((Fluoro(naphthalen‐2‐yl)methyl)sulfonyl)benzo[d]thiazole (3 c)**: The compound was synthesised from **2 c** (80 mg, 0.24 mmol, 1.00 equiv.) according to General Procedure 5 (reaction time: 6 h). The product was purified by flash chromatography (CyH:EtOAc/ 14:1) and was obtained as a light yellow solid (29 mg, 35 %). M.p.: 166 °C; *R*_f_ 0.28 (CyH:EtOAc/ 5:1); HRMS *m*/*z* (ESI): Calcd. for C_18_H_12_NO_2_S_2_FNa^+^ 380.0186, found 380.0196; ^1^H NMR (600 MHz, Methylene chloride‐*d_2_*, 298 K) *δ* 8.31 (ddd, ^3^ *J*HH=8.2 Hz, ^4^ *J*HH=1.2 Hz, ^5^ *J*HH=0.7 Hz, 1 H, HC4), 8.13 (s, 1 H, HC14), 8.08 (ddd, ^3^ *J*HH=8.1 Hz, ^4^ *J*HH=1.2 Hz, ^5^ *J*HH=0.7 Hz, 1 H, HC7), 7.98 (d, ^3^ *J*HH=8.5 Hz, 1 H, HC11), 7.93 (m, 2 H, HC15,18), 7.71 (ddd, ^3^ *J*HH=8.3, 7.2 Hz, ^4^ *J*HH=1.3 Hz, 1 H, HC5), 7.66 (m, 2 H, HC6,10), 7.62 (ddd, ^3^ *J*HH=8.4, 6.9 Hz, ^4^ *J*HH=1.5 Hz, 1 H, HC16), 7.59 (ddd, ^3^ *J*HH=7.9, 6.8 Hz, ^4^ *J*HH=1.4 Hz, 1 H, HC17), 6.81 (d, ^2^ *J*FH=45.6 Hz, 1 H, HC8); ^13^C{^1^H} NMR (151 MHz, Methylene chloride‐*d_2_*, 298 K) *δ* 163.3 (C1), 153.5 (C3), 138.2 (C2), 135.2 (d, ^5^ *J*FC=1.1 Hz, C12), 133.2 (C13), 129.8 (d, ^3^ *J*FC=7.1 Hz, C14), 129.3 (C11), 129.1 (C18), 129.0 (C6), 128.6 (C16), 128.5 (C5), 128.5 (C15), 127.6 (C17), 126.2 (C4), 124.6 (d, ^3^ *J*FC=5.5 Hz, C10), 124.4 (d, ^2^ *J*FC=19.4 Hz, C9), 123.0 (C7), 102.9 (d, ^1^ *J*FC=222.8 Hz, C8); ^19^F NMR (564 MHz, Methylene chloride‐*d_2_*, 298 K) δ−172.3 (d, ^2^ *J*FH=45.6 Hz, 1F, FC8); IR (cm^−1^): 2923 (w), 2852 (w), 1600 (w), 1550 (w), 1507 (w), 1466 (m), 1420 (w), 1367 (w), 1345 (s), 1317 (m), 1276 (m), 1260 (w), 1240 (w), 1222 (m), 1212 (w), 1151 (s), 1123 (m), 1084 (m), 1043 (m), 1020 (m), 968 (m), 954 (m), 910 (m), 885 (w), 867 (m), 852 (m), 830 (m), 776 (m), 764 (s), 741 (m), 732 (s), 694 (m), 654 (s).

**2‐(Benzylsulfinyl)benzo[d]thiazole (4 a)**: The compound was synthesised from **1 a** (1.00 g, 3.89 mmol, 1.00 equiv.) following General Procedure 2 (reaction time: 26 h). The product was purified by flash chromatography (DCM) and was afforded as a white solid (701 mg, 66 %). M.p.: 121 °C; *R*_f_ 0.09 (DCM); HRMS *m*/*z* (ESI): Calcd. for C_14_H_11_NOS_2_Na^+^ 296.0174, found 296.0181; ^1^H NMR (600 MHz, Methylene chloride‐*d_2_*, 298 K) *δ* 8.10 (ddd, ^3^ *J*HH=8.3 Hz, ^4^ *J*HH=1.2 Hz, ^5^ *J*HH=0.7 Hz, 1 H, HC4), 7.97 (ddd, ^3^ *J*HH=8.1 Hz, ^4^ *J*HH=1.2 Hz, ^5^ *J*HH=0.7 Hz, 1 H, HC7), 7.59 (ddd, ^3^ *J*HH=8.3, 7.2 Hz, ^4^ *J*HH=1.2 Hz, 1 H, HC5), 7.50 (ddd, ^3^ *J*HH=8.3, 7.2 Hz, ^4^ *J*HH=1.2 Hz, 1 H, HC6), 7.34–7.26 (m, 3 H, HC11,12), 7.19–7.16 (m, 2 H, HC10), [4.52 (d, ^2^ *J*HH=13.2 Hz, 1 H), 4.34 (d, ^2^ *J*HH=13.1 Hz, 1 H)](H_2_C8); ^13^C{^1^H} NMR (151 MHz, Methylene chloride‐*d_2_*, 298 K) *δ* 177.8 (C1), 154.4 (C3), 136.6 (C2), 131.1 (C10), 129.3 (C9), 129.2 (C12), 129.1 (C11), 127.4 (C5), 126.7 (C6), 124.4 (C4), 122.8 (C7), 63.3 (C8); IR (cm^−1^): 2960 (w), 2907 (w), 1493 (w), 1467 (w), 1455 (w), 1417 (w), 1314 (w), 1275 (w), 1233 (w), 1160 (w), 1134 (w), 1124 (w), 1086 (w), 1074 (w), 1048 (m), 1032 (w), 1004 (w), 1016 (w), 977 (w), 945 (w), 920 (w), 886 (w), 856 (w), 847 (w), 811 (w), 766 (w), 756 (m), 732 (m), 697 (m), 684 (m).

**2‐(Phenethylsulfinyl)benzo[d]thiazole (4 b)**: The compound was synthesised from **1 b** (300 mg, 1.11 mmol, 1.00 equiv.) following General Procedure 2 (reaction time: 24 h). The product was purified via flash chromatography (DCM) and was obtained as a white solid (185 mg, 58 %). M.p.: 84 °C; *R*_f_ 0.10 (DCM); HRMS *m*/*z* (ESI): Calcd. for C_15_H_13_NS_2_ONa^+^ 310.0331, found 310.0338; ^1^H NMR (600 MHz, Methylene chloride‐*d_2_*, 298 K) *δ* 8.07 (ddd, ^3^ *J*HH=8.2 Hz, ^4^ *J*HH=1.2 Hz, ^5^ *J*HH=0.7 Hz, 2 H, HC4), 8.04 (ddd, ^3^ *J*HH=8.1 Hz, ^4^ *J*HH=1.3 Hz, ^5^ *J*HH=0.7 Hz, 1 H, HC7), 7.58 (ddd, ^3^ *J*HH=8.3, 7.2 Hz, ^4^ *J*HH=1.3 Hz, 1 H, HC5), 7.51 (ddd, ^3^ *J*HH=8.1, 7.2 Hz, ^4^ *J*HH=1.2 Hz, 1 H, HC6), 7.29 (m, 2 H, HC11), 7.25–7.20 (m, 3 H, HC12,13), [3.52 (ddd, ^2^ *J*HH=13.3 Hz, ^3^ *J*HH=10.2, 6.3 Hz, 1 H), 3.45 (ddd, ^2^ *J*HH=13.3 Hz, ^3^ *J*HH=10.0, 5.5 Hz, 1 H)](H_2_C8), [3.25 (ddd, ^2^ *J*HH=14.1 Hz, ^3^ *J*HH=10.0, 6.3 Hz, 1 H), 2.98 (ddd, ^2^ *J*HH=14.1 Hz, ^3^ *J*HH=10.2, 5.5 Hz, 1 H)]( H_2_C9); ^13^C{^1^H} NMR (151 MHz, Methylene chloride‐*d_2_*, 298 K) *δ* 178.2 (C1), 154.7 (C3), 139.0 (C10), 136.7 (C2), 129.3 (C12), 129.2 (C11), 127.5 (C5), 127.3 (C13), 126.7 (C6), 124.4 (C4), 122.9 (C7), 58.0 (C8), 28.1 (C9); IR (cm^−1^): 2920 (w), 1601 (w), 1557 (w), 1497 (m), 1471 (m), 1455 (m), 1428 (m), 1401 (m), 1312 (m), 1279 (w), 1259 (w), 1233 (m), 1219 (m), 1202 (w), 1180 (w), 1157 (w), 1129 (w), 1078 (m), 1047 (s), 1020 (m), 999 (m), 969 (w), 946 (m), 919 (w), 863 (w), 843 (m), 832 (w), 759 (s), 747 (s), 731 (s), 700 (s), 683 (m), 666 (m).

**2‐((Naphthalen‐2‐ylmethyl)sulfinyl)benzo[d]thiazole (4 c)**: The product was synthesised from **1 c** (140 mg, 0.46 mmol, 1.00 equiv.) following General Procedure 2 (reaction time: 24 h). The desired product was purified by flash chromatography (DCM) and was obtained as a white solid (81 mg, 54 %). M.p.: 152 °C; *R*_f_ 0.23 (DCM); HRMS *m*/*z* (ESI): Calcd. for C_18_H_13_NOS_2_Na^+^ 346.0331, found 346.0328; ^1^H NMR (400 MHz, Methylene chloride‐*d_2_*, 298 K) *δ* 8.12 (d, ^3^ *J*HH=8.3 Hz, 1 H, HC4), 7.94 (d, ^3^ *J*HH=8.1 Hz, 1 H, HC7), 7.85–7.79 (m, 1 H, HC15), 7.74 (d, ^3^ *J*HH=8.4 Hz, 2 H, HC11,18), 7.69 (s, 1 H, HC14), 7.60 (ddd, ^3^ *J*HH=8.5, 7.2 Hz, ^4^ *J*HH=1.3 Hz, 1 H, HC5), 7.55–7.44 (m, 3 H, HC6,16,17), 7.24 (dd, ^3^ *J*HH=8.4 Hz, ^4^ *J*HH=1.8 Hz, 1 H, HC10), [4.69 (d, ^2^ *J*HH=13.1 Hz, 1 H), 4.51 (d, ^2^ *J*HH=13.0 Hz, 1 H)](H_2_C8); ^13^C{^1^H} NMR (101 MHz, Methylene chloride‐*d_2_*, 298 K) *δ* 177.8 (C1), 154.5 (C3), 136.6 (C2), [133.7, 133.6](C12,13), 130.7 (C14), 128.8 (C11), 128.4 (C18), 128.2 (C10,15), 127.4 (C5), 127.1 (C16), 127.0 (C17), 126.8 (C9), 126.7 (C6), 124.4 (C4), 122.8 (C7), 63.6 (C8); IR (cm^−1^): 2919 (w), 1696 (w), 1596 (w), 1575 (w), 1556 (w), 1508 (w), 1473 (m), 1455 (w), 1425 (m), 1367 (w), 1313 (m), 1274 (w), 1261 (m), 1240 (w), 1206 (w), 1145 (w), 1120 (m), 1085 (m), 1075 (m), 1048 (s), 1015 (m), 999 (m), 969 (w), 955 (m), 943 (m), 902 (m), 874 (m), 846 (w), 823 (m), 776 (w), 756 (m), 743 (s), 728 (s), 676 (m), 667 (m).

**2‐((1‐Fluoro‐2‐phenylethyl)thio)benzo[d]thiazole (5 b)**: A flame dried Schlenk flask was charged with Selectfluor^®^ (160 mg, 0.46 mmol, 1.25 equiv.) and ACN (2 mL). Thioether **1 b** (100 mg, 0.36 mmol, 1.00 equiv.) in ACN (2 mL) was then added dropwise to the suspension and the mixture was stirred under argon at room temperature for 15 min where after Et_3_N (62 μL, 0.46 mmol, 1.25 equiv.) was added. The reaction mixture was stirred for another 15 min at room temperature before it was poured into water, extracted with DCM (*x* 3), dried (Na_2_SO_4_) and concentrated in vacuo. The product was purified through flash chromatography (CyH:EtOAc/ 15:1→5:1) followed by preparative TLC (CyH:EtOAc/ 10:1×1 and 5:1×2) and was obtained as a colourless oil (19 mg, 21 %). *R*_f_ 0.48 (CyH:EtOAc/ 5:1); HRMS *m*/*z* (ESI): Calcd. for C_15_H_12_NS_2_FNa 312.0287, found 312.0284; ^1^H NMR (600 MHz, Methylene chloride‐*d_2_*, 298 K) *δ* 7.93 (ddd, ^3^ *J*HH=8.2 Hz, ^4^ *J*HH=1.2 Hz, ^5^ *J*HH=0.6 Hz, 1 H, HC4), 7.83 (ddd, ^3^ *J*HH=8.0 Hz, ^4^ *J*HH=1.3 Hz, ^5^ *J*HH=0.6 Hz, 1 H, HC7), 7.47 (ddd, ^3^ *J*HH=8.3, 7.2 Hz, ^4^ *J*HH=1.2 Hz, 1 H, HC5), 7.39–7.30 (m, 6 H, HC6,11,12,13), 6.82 (dt, ^2^ *J*FH=53.1 Hz, ^3^ *J*HH=6.1 Hz, 1 H, HC8), 3.42 (dd, ^3^ *J*FH=19.4 Hz, ^3^ *J*HH=6.0 Hz, 2 H, H_2_C9); ^13^C{^1^H} NMR (151 MHz, Methylene chloride‐*d_2_*, 298 K) *δ* 162.7 (d, ^3^ *J*FC=2.8 Hz, C1), 153.6 (C3), 136.4 (C2), 135.4 (d, ^3^ *J*FC=3.2 Hz, C10), 130.23 (C11), 129.21 (C12), 128.08 (C13), 126.90 (C5), 125.52 (C6), 122.72 (C4), 121.75 (C7), 99.91 (d, ^1^ *J*FC=225.3 Hz, C8), 41.79 (d, ^2^ *J*FC=22.1 Hz, C9); ^19^F NMR (564 MHz, Methylene chloride‐*d_2_*, 298 K) δ−148.0 (dt, ^2^ *J*FH=53.1 Hz, ^3^ *J*FH=19.5 Hz, 1F, FC8); IR (cm^−1^): 1495 (w), 1455 (m), 1425 (m), 1310 (m), 1275 (w), 1237 (w), 1203 (w), 1183 (w), 1158 (w), 1126 (w), 1078 (w), 1053 (w), 1018 (w), 980 (s), 849 (m), 822 (m), 753 (s), 725 (s), 696 (s), 667 (m).

**2‐((Fluoro(phenyl)methyl)sulfinyl)benzo[d]thiazole (6 a/b)**: The compounds were synthesised from **1 a** (700 mg, 2.72 mmol, 1.00 equiv.) according to General Procedure 4 (reaction time: 17 h). The crude product was adsorbed on silica and purified via flash chromatography (*n*‐Pentane:Et_2_O/ 15:1) which afforded the products as white solids (**6 a**: 78 mg, 9 % and **6 b**: 41 mg, 5 %). **6 a** (*anti*): M.p.: 136 °C; *R*_f_ 0.27 (*n*‐Pentane:Et_2_O/ 4:1); HRMS *m*/*z* (ESI): Calcd. for C_14_H_10_FNOS_2_Na^+^ 314.0086, found 314.0092; ^1^H NMR (600 MHz, Methylene chloride‐*d_2_*, 298 K) *δ* 8.14 (ddd, ^3^ *J*HH=8.3 Hz, ^4^ *J*HH=1.2 Hz, ^5^ *J*HH=0.7 Hz, 1 H, HC4), 7.95 (ddd, ^3^ *J*HH=8.2 Hz, ^4^ *J*HH=1.2 Hz, ^5^ *J*HH=0.7 Hz, 1 H, HC7), 7.61 (ddd, ^3^ *J*HH=8.3, 7.2 Hz, ^4^ *J*HH=1.2 Hz, 1 H, HC5), 7.51 (ddd, ^3^ *J*HH=8.2, 7.1 Hz, ^4^ *J*HH=1.2 Hz, 1 H, HC6), 7.42 (tq, ^3^ *J*HH=7.3 Hz, ^4^ *J*HH=1.2 Hz, 1 H, HC12), 7.30 (tt, ^3^ *J*HH=7.5 Hz, ^4^ *J*HH=1.0 Hz, 2 H, HC11), 7.15–7.08 (m, 2 H, HC10), 6.54 (d, ^2^ *J*FH=45.6 Hz, 1 H, HC8); ^1^H NMR (600 MHz, Methylene Chloride‐*d*_2_, 193 K) *δ* 8.12 (d, ^3^ *J*HH=8.2 Hz, 1 H, HC4), 7.93 (d, ^3^ *J*HH=8.1 Hz, 1 H, HC7), 7.61 (ddd, ^3^ *J*HH=8.3, 7.1 Hz, ^4^ *J*HH=1.3 Hz, 1 H, HC5), 7.50 (td, ^3^ *J*HH=7.6, 6.8 Hz, ^4^ *J*HH=1.3 Hz, 1 H, HC6), 7.39 (t, ^3^ *J*HH=7.4 Hz, 1 H, HC12), 7.25 (t, ^3^ *J*HH=7.6 Hz, 2 H, HC11), 6.95 (d, ^3^ *J*HH=7.5 Hz, 2 H, HC10), 6.62 (d, ^2^ *J*FH=44.5 Hz, 1 H, HC8); ^13^C{^1^H} NMR (151 MHz, Methylene chloride‐*d_2_*, 298 K) *δ* 174.3 (d, ^3^ *J*FC=10.3 Hz, C1), 154.4 (C3), 136.5 (C2), 131.3 (d, ^5^ *J*FC=1.6 Hz, C12), 128.8 (d, ^2^ *J*FC=19.6 Hz, C9), 128.8 (C11), 128.1 (d, ^3^ *J*FC=6.2 Hz, C10), 127.6 (C5), 127.0 (C6), 124.7 (C4), 122.8 (C7), 109.7 (d, ^1^ *J*FC=218.7 Hz, C8); ^13^C{^1^H} NMR (151 MHz, Methylene Chloride‐*d*_2_, 193 K) *δ* 173.0 (d, ^3^ *J*FC=11.7 Hz, C1), 153.4 (C3), 135.1 (C2), 130.6 (C12), 128.0 (C11), 127.1 (d, ^3^ *J*FC=5.9 Hz, C10), 126.9 (C5), 126.9 (d, ^2^ *J*FC=19.7 Hz, C9), 126.2 (C6), 123.7 (C4), 122.3 (C7), 109.0 (d, ^1^ *J*FC=215.6 Hz, C8); ^19^F NMR (564 MHz, Methylene chloride‐*d_2_*, 298 K) δ−171.5 (d, ^2^ *J*FH=45.6 Hz, 1F, FC8); ^19^F NMR (564 MHz, Methylene Chloride‐*d*_2_, 193 K) δ−171.6 (d, ^2^ *J*FH=44.5 Hz, 1F, FC8); IR (cm^−1^):3031 (w), 2959 (w), 2924 (w), 1556 (w), 1495 (w), 1470 (m), 1456 (m), 1429 (m), 1353 (w), 1331 (w), 1312 (m), 1292 (w), 1261 (w), 1228 (m), 1191 (w), 1144 (m), 1126 (w), 1090 (m), 1070 (s), 1048 (m), 1031 (m), 1012 (m), 997 (s), 957 (m), 918 (m), 875 (w), 842 (m), 762 (s), 733 (m), 693 (s), 671 (s). **6 b** (*syn*): M.p.: 125 °C; *R*_f_ 0.19 (*n*‐Pentane:Et_2_O/ 4:1); HRMS *m*/*z* (ESI): Calcd. for C_14_H_10_FNOS_2_Na^+^ 314.0086, found 314.0091; ^1^H NMR (600 MHz, Methylene chloride‐*d_2_*, 298 K) *δ* 8.07 (ddd, ^3^ *J*HH=8.2 Hz, ^4^ *J*HH=1.2 Hz, ^5^ *J*HH=0.7 Hz, 1 H, HC4), 8.01 (ddd, ^3^ *J*HH=8.0 Hz, ^4^ *J*HH=1.2 Hz, ^5^ *J*HH=0.7 Hz, 1 H, HC7), 7.59 (ddd, ^3^ *J*HH=8.3, 7.2 Hz, ^4^ *J*HH=1.3 Hz, 1 H, HC5), 7.53 (ddd, ^3^ *J*HH=8.1, 7.2 Hz, ^4^ *J*HH=1.2 Hz, 1 H, HC6), 7.47 (m, 1 H, HC12), 7.39 (m, 4 H, HC10,11), 6.46 (d, ^2^ *J*FH=46.1 Hz, 1 H, HC8); ^1^H NMR (600 MHz, Methylene chloride‐*d_2_*, 193 K) *δ* 8.06 (d, ^3^ *J*HH=8.1 Hz, 1 H, HC4), 8.04 (d, ^3^ *J*HH=8.2 Hz, HC7), 7.59 (t, ^3^ *J*HH=7.6 Hz, 1 H, HC5), 7.53 (t, ^3^ *J*HH=7.5 Hz, 1 H, HC6), 7.48 (t, ^3^ *J*HH=7.3 Hz, 1 H, HC12), 7.43 (t, ^3^ *J*HH=7.4 Hz, 2 H, HC11), 7.38 (d, ^3^ *J*HH=7.6 Hz, 2 H, HC10), 6.56 (d, ^2^ *J*HH=45.1 Hz, 1 H, HC8); ^13^C{^1^H} NMR (151 MHz, Methylene chloride‐*d_2_*, 298 K) *δ* 173.3 (d, ^3^ *J*FC=5.8 Hz, C1), 154.3 (C3), 136.7 (C2), 131.4 (d, ^5^ *J*FC=1.5 Hz, C12), 130.4 (d, ^2^ *J*FC=19.6 Hz, C9), 129.4 (C11), 127.6 (C5), 127.3 (d, ^3^ *J*FC=6.4 Hz, C10), 127.1 (C6), 124.7 (C4), 122.9 (C7), 108.1 (d, ^1^ *J*FC=231.7 Hz, C8); ^13^C{^1^H} NMR (151 MHz, Methylene chloride‐*d_2_*, 193 K) *δ* 172.4 (d, ^3^ *J*FC=5.5 Hz, C1), 153.2 (C3), 135.4 (C2), 130.7 (C12), 129.1 (d, ^2^ *J*FC=19.9 Hz, C9), 128.7 (C11), 126.9 (C5), 126.3 (C6), 126.2 (d, ^3^ *J*FC=6.5 Hz, C10), 123.6 (C6), 122.3 (C4), 106.1 (d, ^1^ *J*FC=233.8 Hz, C8); ^19^F NMR (564 MHz, Methylene chloride‐*d_2_*, 298 K) δ−179.0 (d, ^2^ *J*FH=46.1 Hz, 1F, FC8); ^19^F NMR (564 MHz, Methylene chloride‐*d_2_*, 193 K) δ−182.7 (d, ^2^ *J*FH=42.9 Hz, 1F, FC8); IR (cm^−1^): 3068 (w), 2947 (w), 2922 (w), 2851 (w), 1585 (w), 1556 (w), 1495 (w), 1476 (w), 1463 (m), 1456 (m), 1421 (m), 1352 (w), 1315 (m), 1275 (w), 1225 (m), 1188 (w), 1162 (w), 1124 (w), 1089 (m), 1069 (m), 1038 (m), 1017 (m), 945 (m), 930 (w), 861 (w), 846 (w), 836 (w), 775 (m), 756 (s), 732 (m), 697 (s), 685 (m), 663 (s).

**2‐((1‐Fluoro‐2‐phenylethyl)sulfinyl)benzo[d]thiazole (7 a/b)**: The products were synthesised from **1 b** (700 mg, 2.58 mmol, 1.00 equiv.) according to General Procedure 4 (reaction time: 45 h). The crude was adsorbed on silica and purified by flash chromatography (CyH:EtOAc/ 100:1→50:1) which afforded the products as white solids (**7 a**: 46 mg, 6 % and **7 b**: 26 mg, 3 %). **7 a** (*anti*): M.p.: 114 °C; *R*_f_ 0.20 (CyH:EtOAc/ 6:1); HRMS *m*/*z* (ESI): Calcd. for C_15_H_12_NOS_2_FNa^+^ 328.0237, found 328.0243; ^1^H NMR (600 MHz, Methylene chloride‐*d_2_*, 298 K) *δ* 8.12 (ddd, ^3^ *J*HH=8.3 Hz, ^4^ *J*HH=1.2 Hz, ^5^ *J*HH=0.7 Hz, 1 H, HC4), 8.05 (ddd, ^3^ *J*HH=8.1 Hz, ^4^ *J*HH=1.2 Hz, ^5^ *J*HH=0.7 Hz, 1 H, HC7), 7.61 (ddd, ^3^ *J*HH=8.3, 7.2 Hz, ^4^ *J*HH=1.3 Hz, 1 H, HC5), 7.54 (ddd, ^3^ *J*HH=8.1, 7.2 Hz, ^4^ *J*HH=1.2 Hz, 1 H, HC6), 7.28–7.24 (m, 2 H, HC12), 7.23–7.18 (m, 3 H, HC11,13), 5.83 (ddd, ^2^ *J*FH=48.2 Hz, ^3^ *J*HH=8.9, 3.6 Hz, 1 H, HC8), [3.42 (ddd, ^3^ *J*FH=16.9 Hz, ^2^ *J*HH=15.1 Hz, ^3^ *J*HH=8.9 Hz, 1 H), 3.18 (ddd, ^3^ *J*FH=34.1 Hz, ^2^ *J*HH=15.2 Hz, ^3^ *J*HH=3.6 Hz, 1 H)](H_2_C9); ^1^H NMR (600 MHz, Methylene chloride‐*d_2_*, 193 K) *δ* 8.11 (d, ^3^ *J*HH=8.2 Hz, 1 H, HC4), 8.08 (d, ^3^ *J*HH=8.1 Hz, 1 H, HC7), 7.61 (t, ^3^ *J*HH=7.7 Hz, 1 H, HC5), 7.55 (t, ^3^ *J*HH=7.7 Hz, 1 H, HC6), 7.27 (m, 2 H, HC12), 7.25–7.20 (m, 1 H, HC13), 7.16 (d, ^3^ *J*HH=7.4 Hz, 2 H, HC11), 5.80 (dd, ^2^ *J*FH=48.1 Hz, ^3^ *J*HH=9.0 Hz, 1 H, HC8), 3.38 (td, ^2^ *J*HH=14.6 Hz, ^3^ *J*HH=9.5 Hz, 1 H, HC9), 2.89 (dd, ^3^ *J*FH=39.0 Hz, ^2^ *J*HH=14.8 Hz, 1 H, H_2_C9); ^13^C{^1^H} NMR (151 MHz, Methylene chloride‐*d_2_*, 298 K) *δ* 173.8 (d, ^3^ *J*FC=10.8 Hz, C1), 154.6 (C3), 136.7 (C2), 134.3 (d, ^3^ *J*FC=2.5 Hz, C10), 130.2 (C11), 129.2 (C12), 127.9 (C13), 127.7 (C5), 127.1 (C6), 124.8 (C4), 122.9 (C7), 109.6 (d, ^1^ *J*FC=222.5 Hz, C8), 33.8 (d, ^2^ *J*FC=19.7 Hz, C9); ^13^C{^1^H} NMR (151 MHz, Methylene chloride‐*d_2_*, 193 K) *δ* 172.5 (d, ^3^ *J*FC=11.7 Hz, C1), 153.6 (C3), 135.5 (C2), 133.1 (C10), 129.5 (C11), 128.5 (C12), 127.2 (C13), 127.0 (C5), 126.3 (C6), 123.8 (C4), 122.3 (C7), 108.3 (d, ^1^ *J*FC=220.8 Hz, C8), 31.8 (d, ^2^ *J*FC=20.0 Hz, C9); ^19^F NMR (564 MHz, Methylene chloride‐*d_2_*, 298 K) δ−177.3 (ddd, ^2^ *J*FH=48.1 Hz, ^3^ *J*FH=34.1, 16.9 Hz, 1F, FC8); ^19^F NMR (564 MHz, Methylene chloride‐*d_2_*, 193 K) δ−179.7 (m, 1F, FC8); IR (cm^−1^): 1602 (w), 1557 (w), 1495 (w), 1470 (m), 1454 (m), 1425 (m), 1342 (w), 1315 (m), 1302 (w), 1232 (m), 1188 (w), 1151 (w), 1124 (w), 1082 (m), 1071 (m), 1051 (s), 996 (m), 945 (m), 918 (w), 854 (m), 760 (s), 729 (s), 717 (m), 695 (s), 681 (m). **7 b** (*syn*): M.p.: 128 °C; *R*_f_ 0.16 (CyH:EtOAc/ 6:1); HRMS *m*/*z* (ESI): Calcd. for C_15_H_12_NOS_2_FNa^+^ 328.0237, found 328.0242; ^1^H NMR (600 MHz, Methylene chloride‐*d_2_*, 298 K) *δ* 8.07 (ddd, ^3^ *J*HH=8.3 Hz, ^4^ *J*HH=1.2 Hz, ^3^ *J*HH=0.7 Hz, 1 H, HC4), 8.05 (ddd, ^3^ *J*HH=8.0 Hz, ^4^ *J*HH=1.3 Hz, ^5^ *J*HH=0.7 Hz, 1 H, HC7), 7.59 (ddd, ^3^ *J*HH=8.3, 7.2 Hz, ^4^ *J*HH=1.3 Hz, 1 H, HC5), 7.53 (ddd, ^3^ *J*HH=8.1, 7.2 Hz, ^4^ *J*HH=1.2 Hz, 1 H, HC6), 7.40–7.30 (m, 5 H, HC11,12,13), 5.69 (ddd, ^2^ *J*FH=47.5 Hz, ^3^ *J*HH=8.1, 5.9 Hz, 1 H, HC8), [3.53 (ddd, ^3^ *J*FH=26.8 Hz, ^2^ *J*HH=14.6 Hz, ^3^ *J*HH=5.9 Hz, 1 H), 3.45 (ddd, ^2^ *J*HH=14.6 Hz, ^2^ *J*FH=14.2 Hz, ^3^ *J*HH=8.1 Hz, H_2_C9); ^1^H NMR (600 MHz, Methylene chloride‐*d_2_*, 193 K) *δ* 8.05 (d, ^3^ *J*HH=8.2 Hz, 1 H, HC4), 8.00 (d, ^3^ *J*HH=8.1 Hz, 1 H, HC7), 7.56 (t, ^3^ *J*HH=7.5 Hz, 1 H, HC5), 7.50 (t, ^3^ *J*HH=7.6 Hz, 1 H, HC6), 7.39–7.27 (m, 5 H, HC11,12,13), 5.72 (dt, ^2^ *J*FH=46.8 Hz, ^3^ *J*HH=7.6 Hz, 1 H, HC8), 3.54–3.43 (m, 2 H, H_2_C9); ^13^C{^1^H} NMR (151 MHz, Methylene chloride‐*d_2_*, 298 K) *δ* 174.1 (d, ^3^ *J*FC=6.5 Hz, C1), 154.5 (C3), 136.7 (C2), 133.9 (d, ^3^ *J*FC=5.3 Hz, C10), 130.3 (C11), 129.5 (C12), 128.3 (C13), 127.6 (C5), 127.0 (C6), 124.5 (C4), 122.9 (C7), 107.6 (d, ^1^ *J*FC=238.8 Hz, C8), 36.2 (d, ^2^ *J*FC=20.5 Hz, C9); ^13^C{^1^H} NMR (151 MHz, Methylene chloride‐*d_2_*, 193 K) 173.1 (d, ^3^ *J*FC=6.4 Hz, C1), 153.4 (C3), 135.4 (C2), 132.6 (d, ^3^ *J*FC=6.3 Hz, C10), 129.6 (C11), 128.8 (C12), 127.6 (C13), 126.9 (C5), 126.1 (C6), 123.3 (C4), 122.3 (C7), 106.5 (d, ^1^ *J*FC=240.3 Hz, C8), 35.2 (d, ^2^ *J*FC=20.5 Hz, C9); ^19^F NMR (564 MHz, Methylene chloride‐*d_2_*, 298 K) δ−181.7 (ddd, ^2^ *J*FH=47.4 Hz, ^3^ *J*FH=26.8, 14.2 Hz, 1F, FC8); ^19^F NMR (564 MHz, Methylene chloride‐*d_2_*, 193 K) δ−183.4 (m, 1F, FC8); IR (cm^−1^): 3062 (w), 2942 (w), 1557 (w), 1497 (w), 1474 (m), 1456 (m), 1429 (m), 1346 (w), 1314 (m), 1261 (m), 1237 (w), 1201 (w), 1187 (w), 1163 (w), 1132 (w), 1086 (m), 1064 (s), 1049 (s), 1019 (s), 1003 (m), 945 (m), 930 (m), 907 (m), 864 (m), 847 (w), 819 (m), 750 (s), 730 (s), 700 (s), 687 (s).

**2‐((Fluoro(naphthalen‐2‐yl)methyl)sulfinyl)benzo[d]thiazole (8 b)**: The compound was prepared from **1 c** (700 mg, 2.28 mmol, 1.00 equiv.) following General Procedure 4 (reaction time: 40 h). The crude mixture was adsorbed on silica and purified via flash chromatography (*n*‐Pentane:Et_2_O/ 15:1→10:1) which afforded the product as a light yellow solid (48 mg, 6 %). M.p.: 135 °C; *R*_f_ 0.22 (CyH:EtOAc/ 6:1); HRMS *m*/*z* (ESI): Calcd. for C_18_H_12_NOS_2_FNa^+^ 364.0240, found 364.0240; ^1^H NMR (600 MHz, Methylene chloride‐*d_2_*, 298 K) *δ* 8.04 (ddd, ^3^ *J*HH=8.2 Hz, ^4^ *J*HH=1.2 Hz, ^5^ *J*HH=0.6 Hz, 1 H, HC4), 7.99 (ddd, ^3^ *J*HH=8.1 Hz, ^4^ *J*HH=1.2 Hz, ^5^ *J*HH=0.6 Hz, 1 H, HC7), 7.90 (s, 1 H, HC14), 7.88 (m, 2 H, HC11,15), 7.83 (m, 1 H, HC18), 7.60–7.50 (m, 4 H, HC5,6,16,17), 7.43 (dd, ^3^ *J*HH=8.5 Hz, ^4^ *J*HH=1.8 Hz, 1 H, HC10), 6.63 (d, ^2^ *J*FH=46.0 Hz, 1 H, HC8); ^13^C{^1^H} NMR (151 MHz, Methylene chloride‐*d_2_*, 298 K) *δ* 173.4 (d, ^3^ *J*FC=5.9 Hz, C1), 154.3 (C3), 136.7 (C2), 134.8 (C12), 133.3 (C13), 129.3 (C11), 128.9 (C18), 128.4 (C15), 128.1 (C16), 127.8 (d, ^3^ *J*FC=7.2 Hz, C14), 127.6 (C5), 127.5 (C11), 127.1 (C6), 124.7 (C4), 123.5 (d, ^3^ *J*FC=5.8 Hz, C10), 122.8 (C7), 108.4 (d, ^1^ *J*FC=232.2 Hz, C8); ^19^F NMR (564 MHz, Methylene chloride‐*d_2_*, 298 K) δ−178.6 (d, ^2^ *J*FH=46.1 Hz, 1F, FC8); IR (cm^−1^): 3063 (w), 2920 (w), 2851 (w), 1680 (w), 1600 (w), 1558 (w), 1508 (w), 1474 (m), 1457 (m), 1427 (m), 1363 (m), 1316 (m), 1271 (m), 1238 (m), 1186 (m), 1152 (m), 1124 (m), 1090 (m), 1041 (m), 1019 (m), 1002 (m), 909 (m), 868 (m), 826 (m), 757 (s), 726 (s), 689 (m), 677 (m).

## Conflict of interest

The authors declare no conflict of interest.

## Supporting information

As a service to our authors and readers, this journal provides supporting information supplied by the authors. Such materials are peer reviewed and may be re‐organized for online delivery, but are not copy‐edited or typeset. Technical support issues arising from supporting information (other than missing files) should be addressed to the authors.

Supplementary
