# Supplementary material for: Conformational Analysis of Acyclic α‐Fluoro Sulfur Motifs
Source: Chemistry. 2020 Sep 28;26(60):13704–15. doi: 10.1002/chem.202003361 (PMC7702044; doi:10.1002/chem.202003361)
Supplement: Supplementary file 1 — Supplementary [file CHEM-26-13704-s001.pdf]

Chemistry–A European Journal

Supporting Information

Conformational Analysis of Acyclic α-Fluoro Sulfur Motifs

Nathalie Erdeljac, Christian Mück-Lichtenfeld, Constantin G. Daniliuc, and Ryan Gilmour*[a]

Supporting Information

Conformational Analysis of Acyclic α-Fluoro Sulfur Motifs

Nathalie Erdeljac,[a] Christian Mück-Lichtenfeld,[a] Constantin Daniliuc,[a] and Ryan Gilmour*[a]

a. Organisch-Chemisches Institut
Westfälische Wilhelms-Universität Münster
Correnstraße 40, 48149 (Germany)
Email: ryan.gilmour@uni-muenster.de
Homepage: http://www.uni-muenster.de/Chemie.oc/gilmour/index.html

Corresponding author: ryan.gilmour@uni-muenster.de

NMR Spectra……………………………………………………………………………S2

X-Ray Crystallographic Data…………………………………………………………S24

Computational Study of Conformers in Solution…………………………………………S37

References……………………………………………………………………………S59

NMR Spectra

2-(Benzylthio)benzo[d]thiazole (1a)

^{1}H NMR (600 MHz, Methylene chloride-d_2, 298 K)

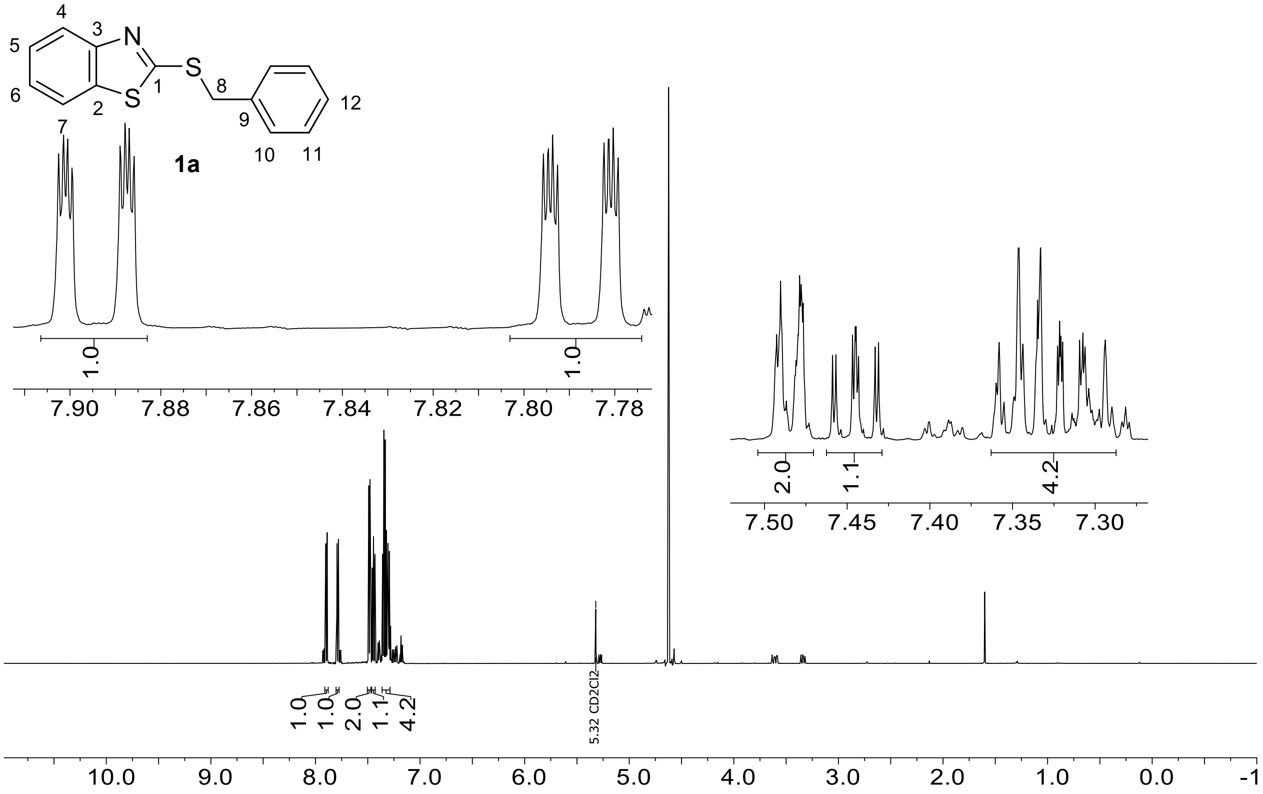

^{13}C{^{1}H} NMR (151 MHz, Methylene chloride-d_2, 298 K)

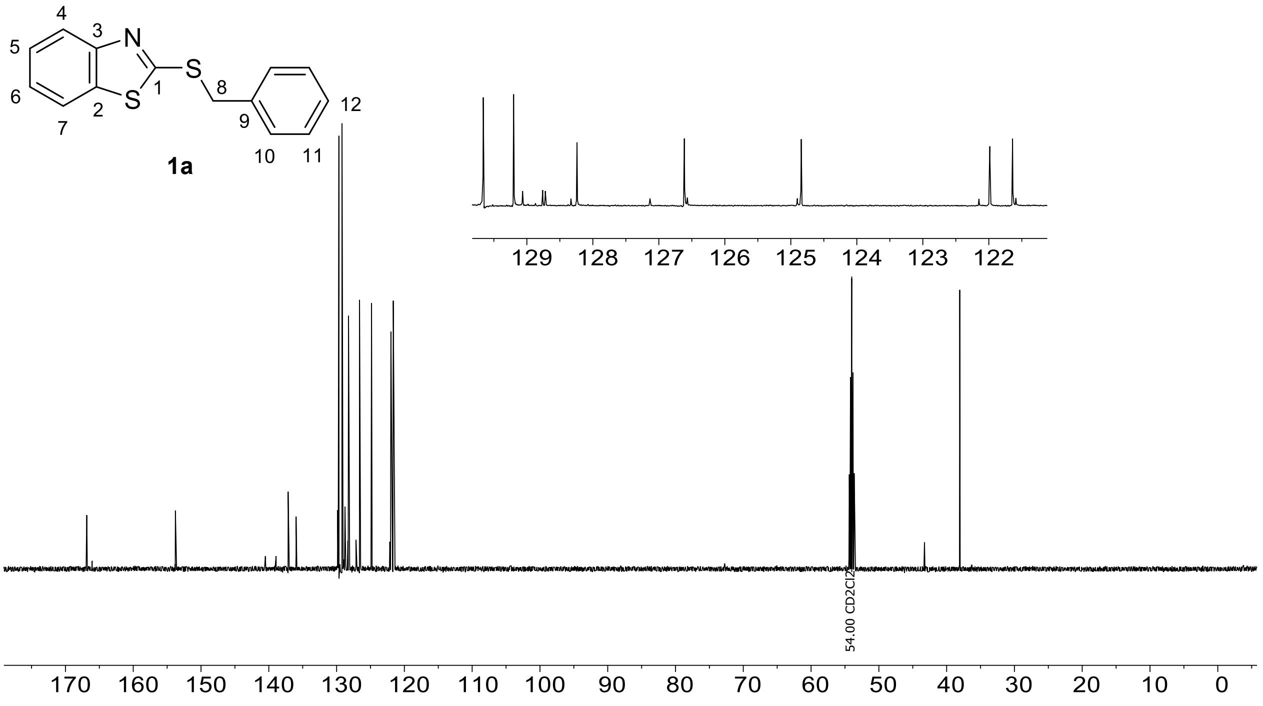

2-(Phenethylthio)benzo[d]thiazole (1b)

^{1}H NMR (600 MHz, Methylene chloride-d_2, 298 K)

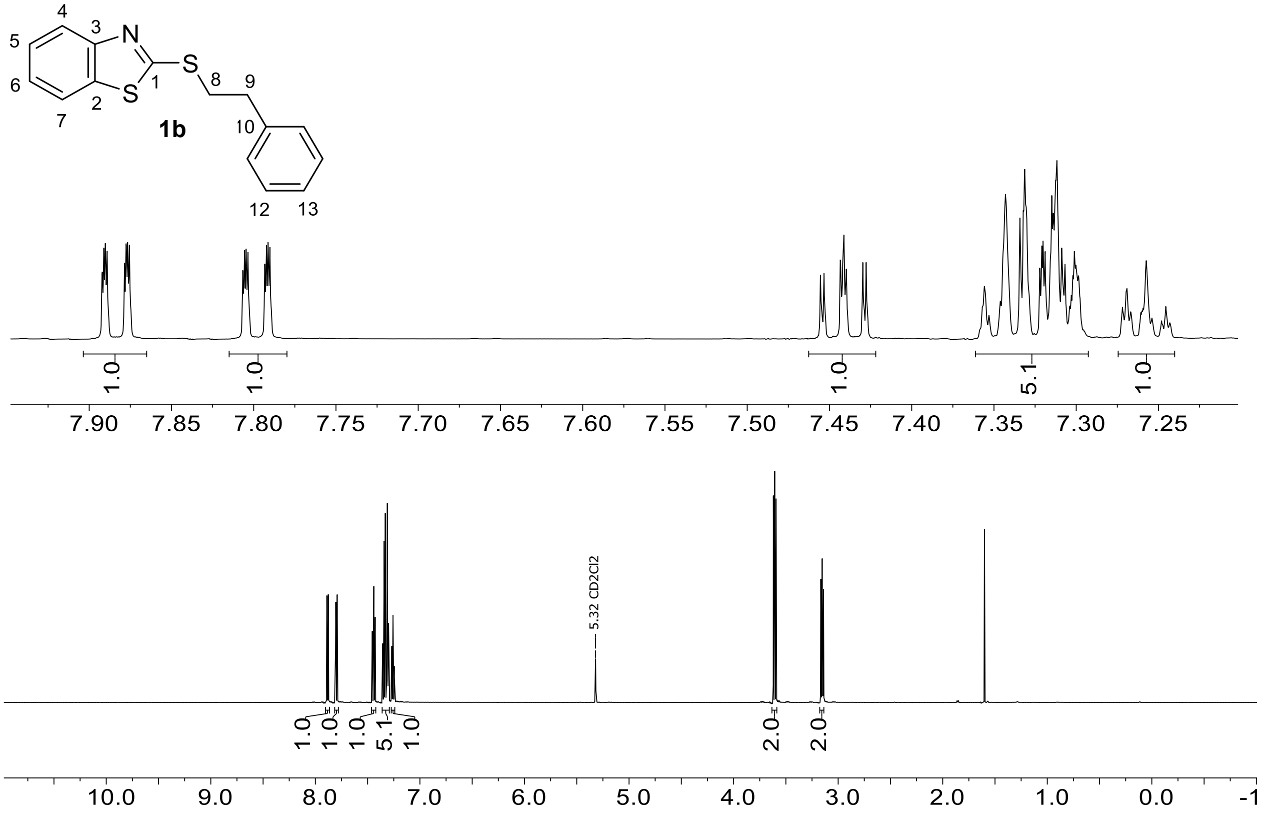

^{13}C{^{1}H} NMR (151 MHz, Methylene chloride-d_2, 298 K)

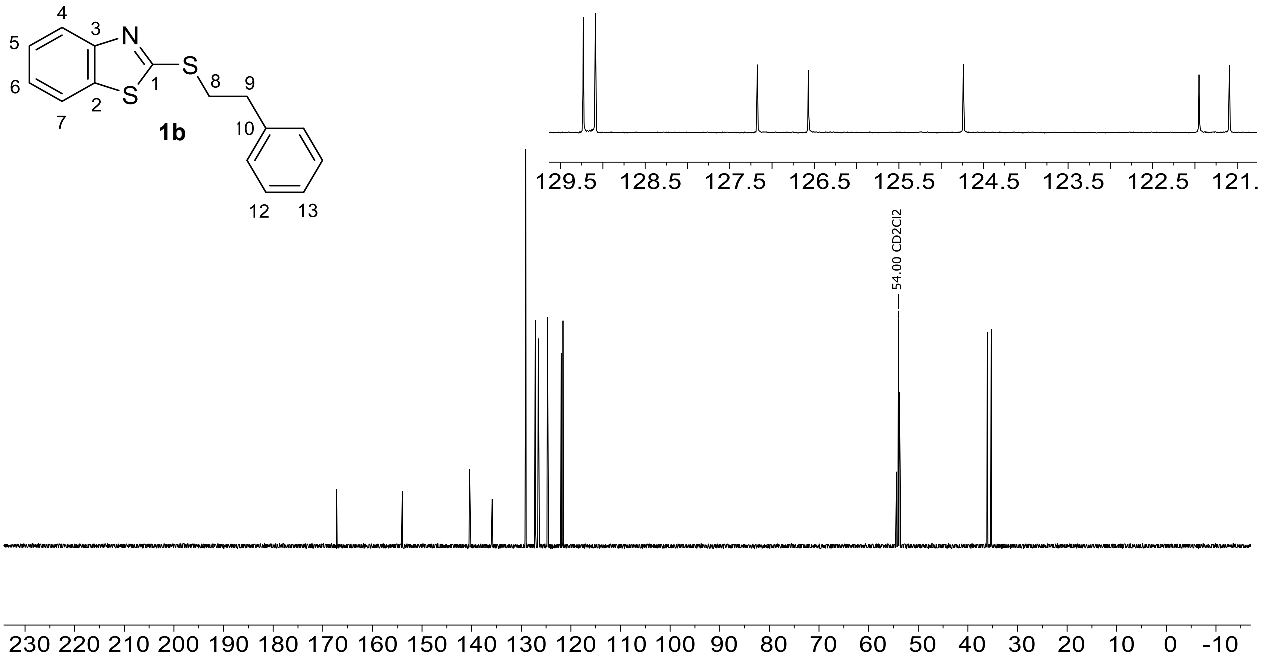

2-((Naphthalen-2-ylmethyl)thio)benzo[d]thiazole (1c)

^{1}H NMR (500 MHz, Methylene chloride-d_2, 298 K)

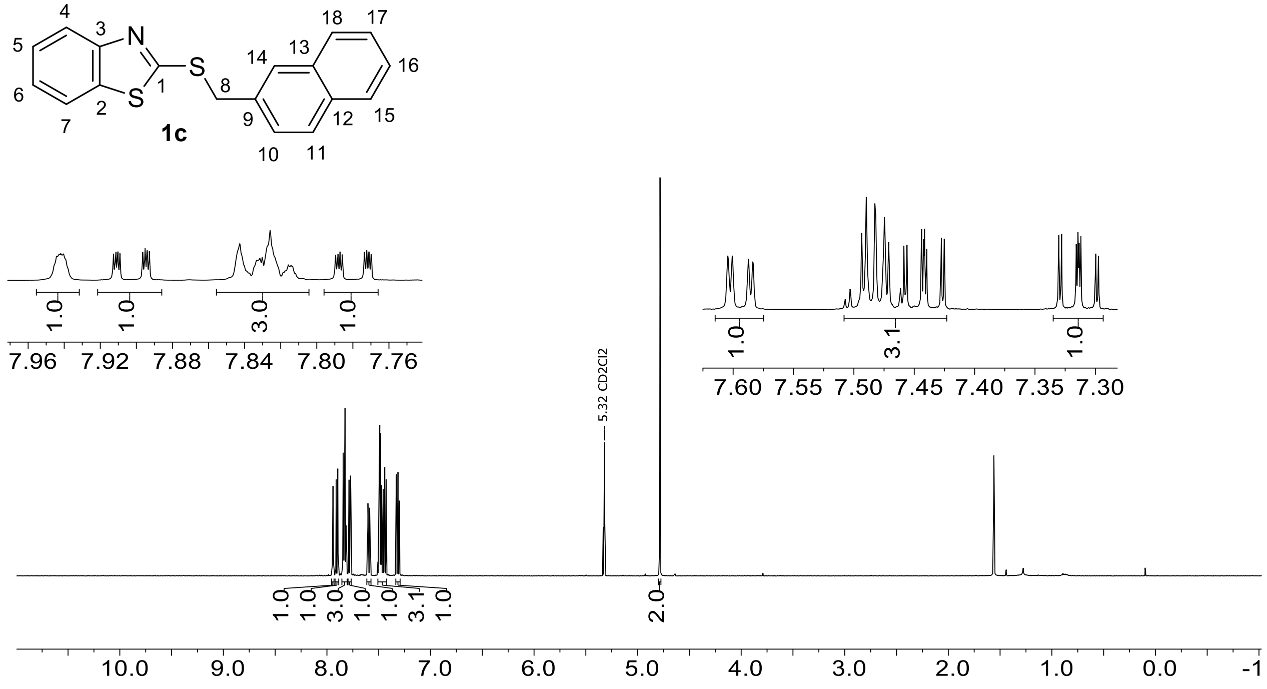

^{13}C{^{1}H} NMR (126 MHz, Methylene chloride-d_2, 298 K)

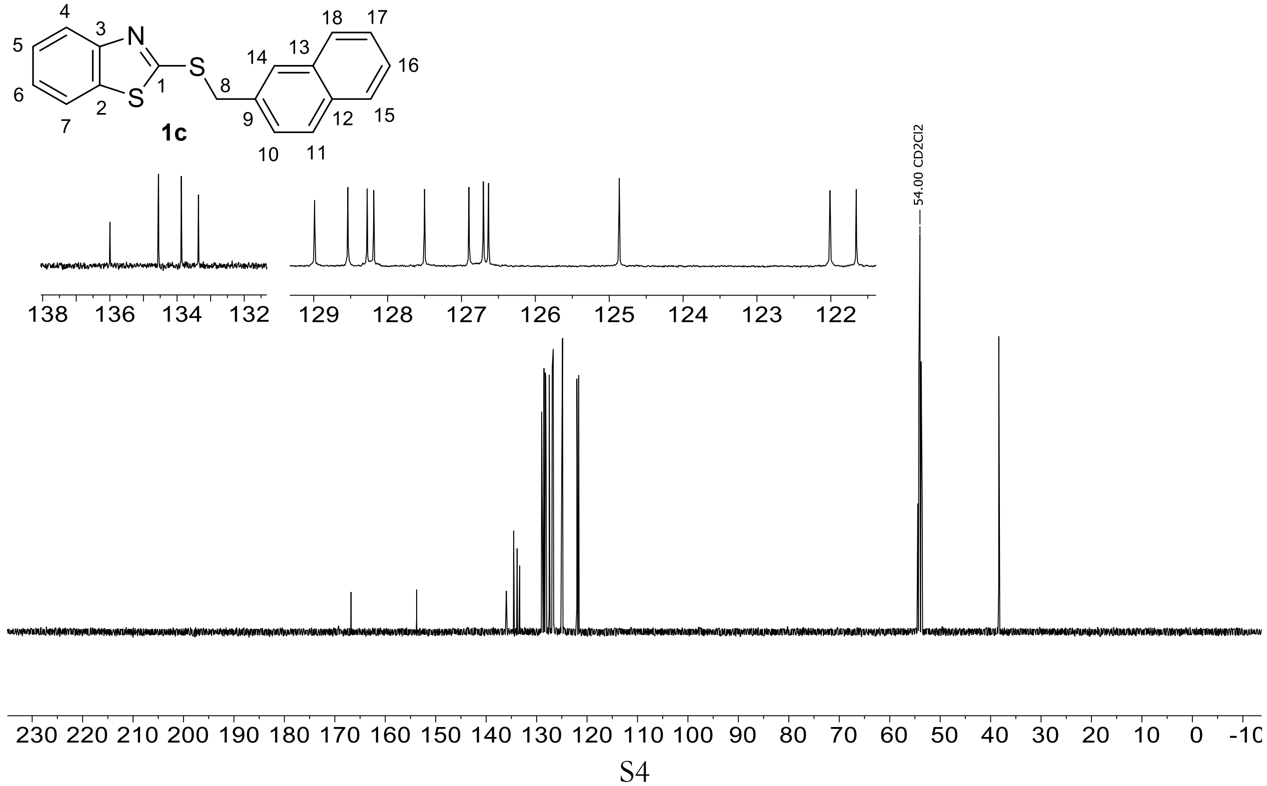

2-(Benzylsulfinyl)benzo[d]thiazole (4a)

^{1}H NMR (600 MHz, Methylene chloride-d_2, 298 K)

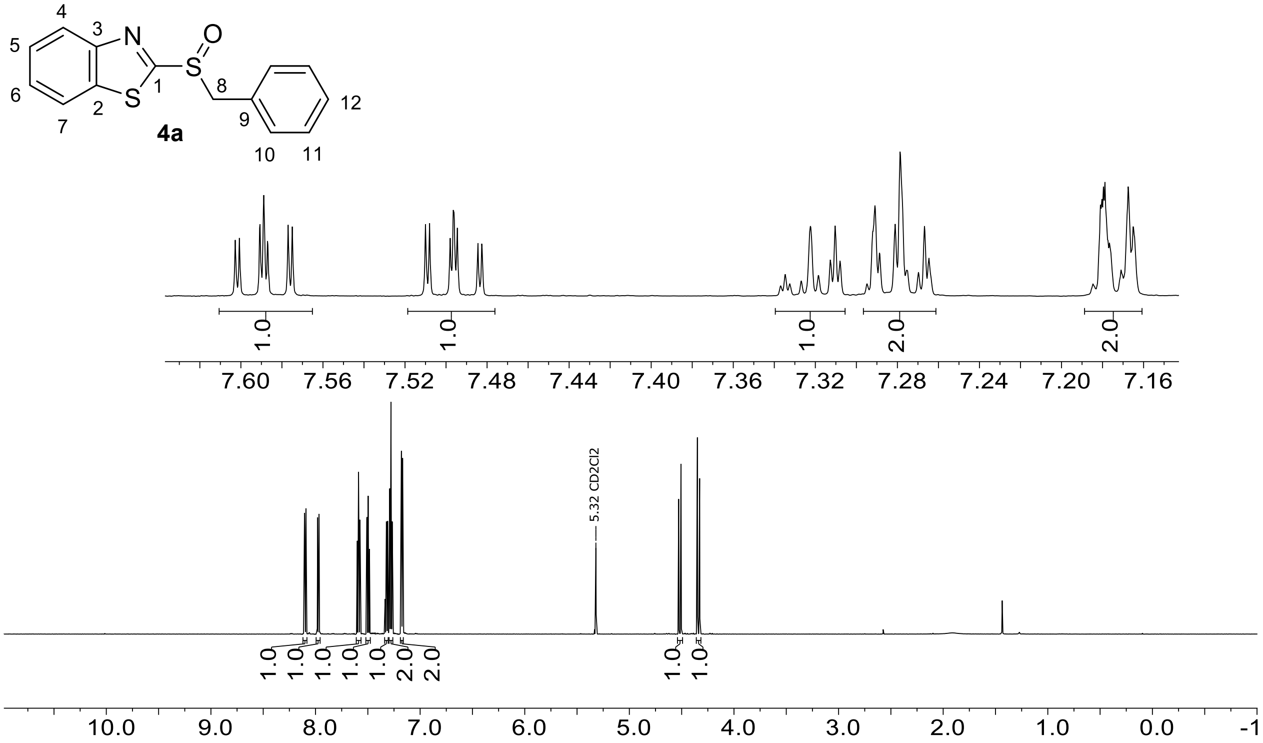

^{13}C{^{1}H} NMR (151 MHz, Methylene chloride-d_2, 298 K)

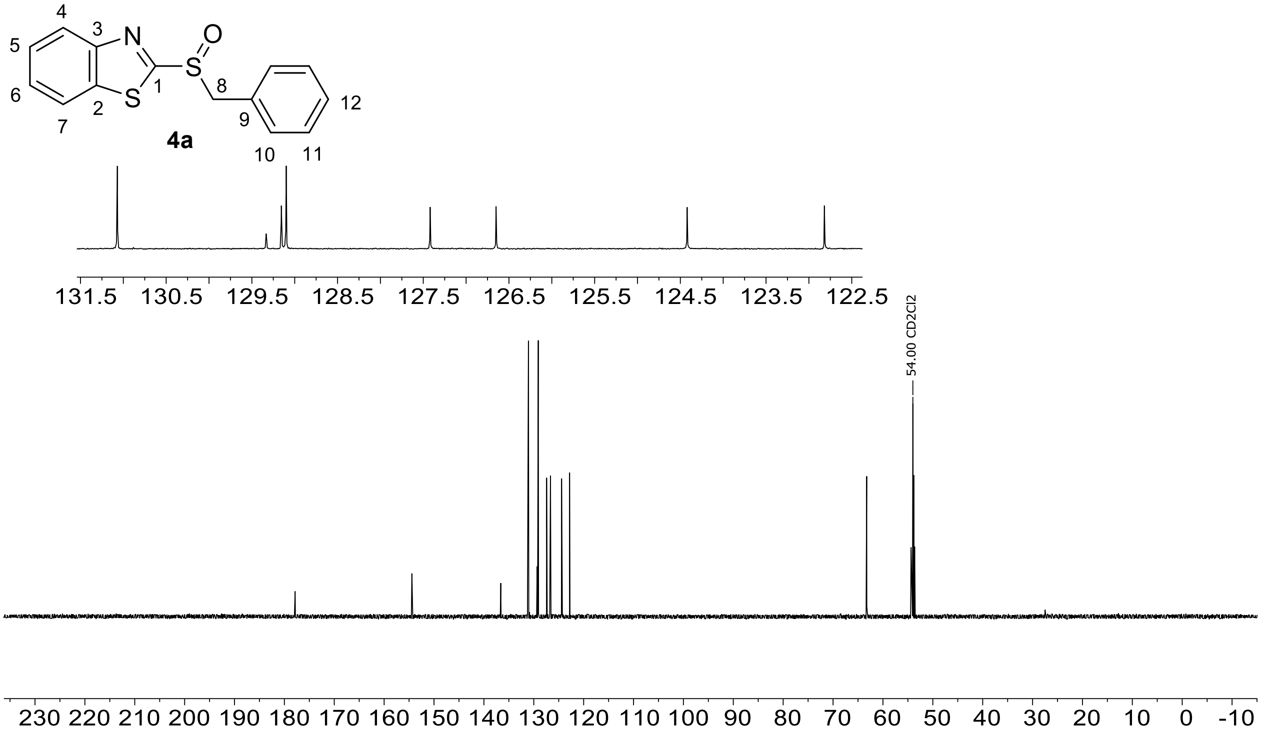

2-(Phenethylsulfinyl)benzo[d]thiazole (4b)

^{1}H NMR (600 MHz, Methylene chloride-d_2, 298 K)

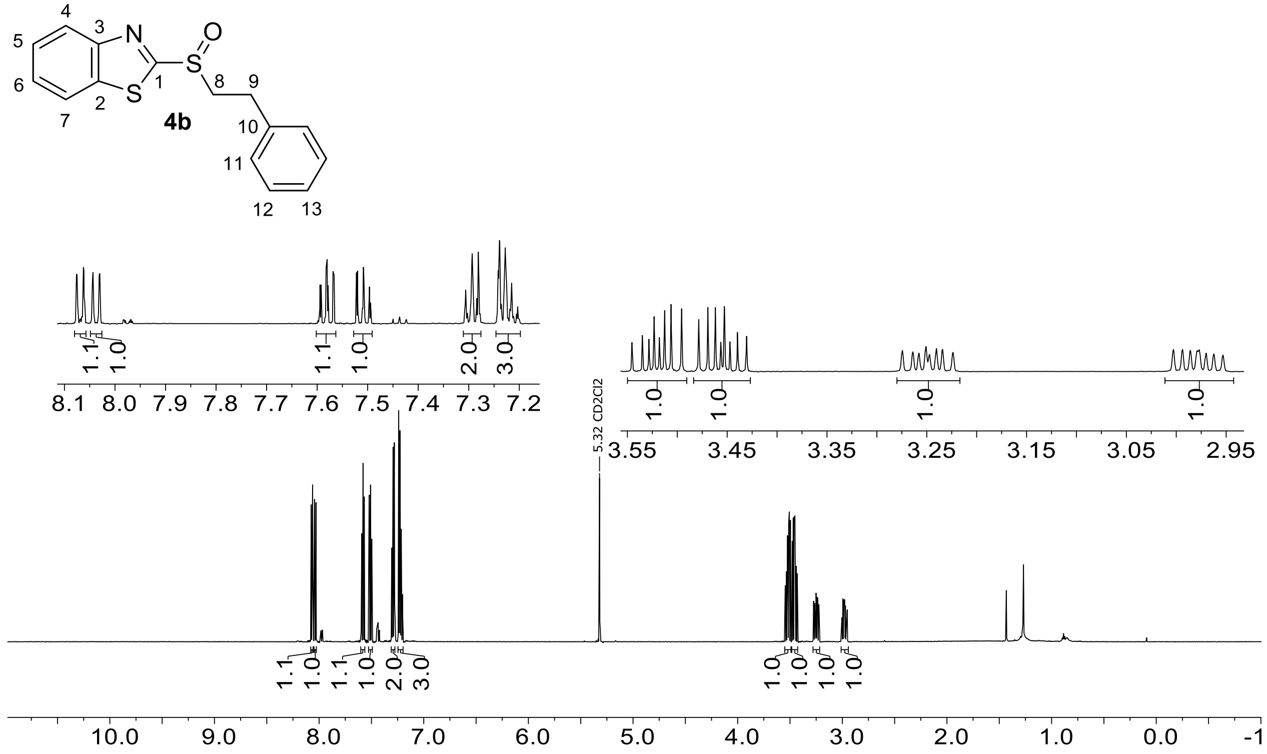

^{13}C{^{1}H} NMR (151 MHz, Methylene chloride-d_2, 298 K)

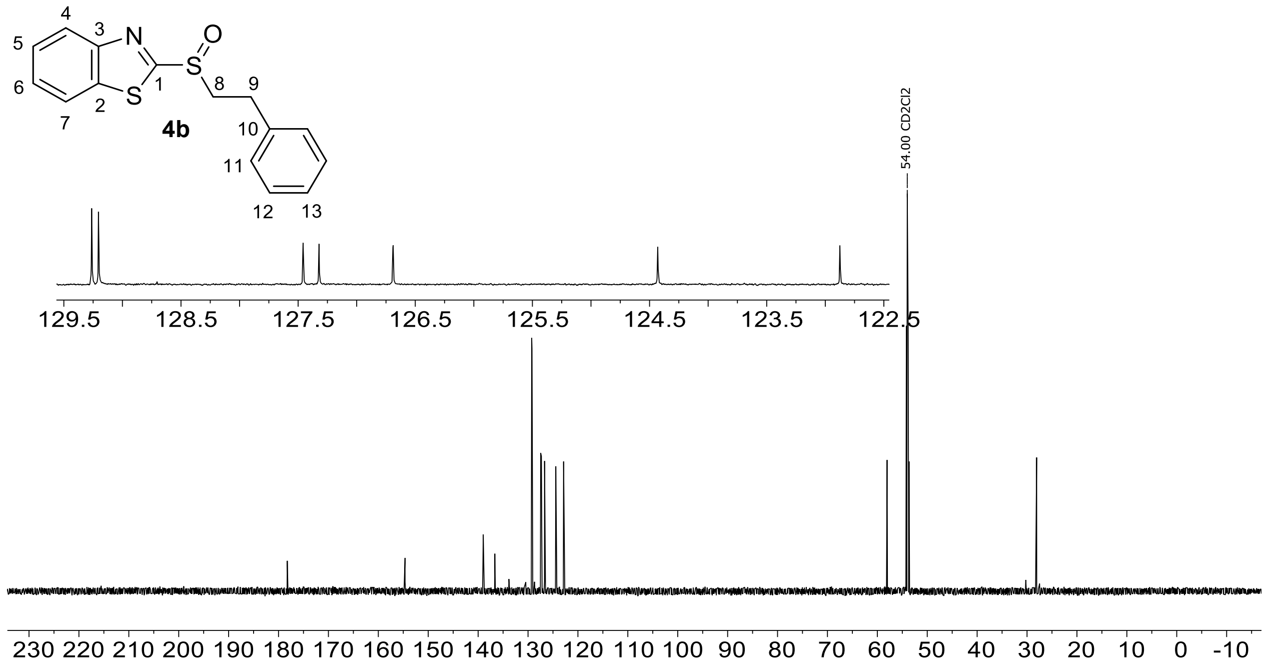

2-((Naphthalen-2-ylmethyl)sulfinyl)benzo[d]thiazole (4c)

^{1}H NMR (400 MHz, Methylene chloride-d_2, 298 K)

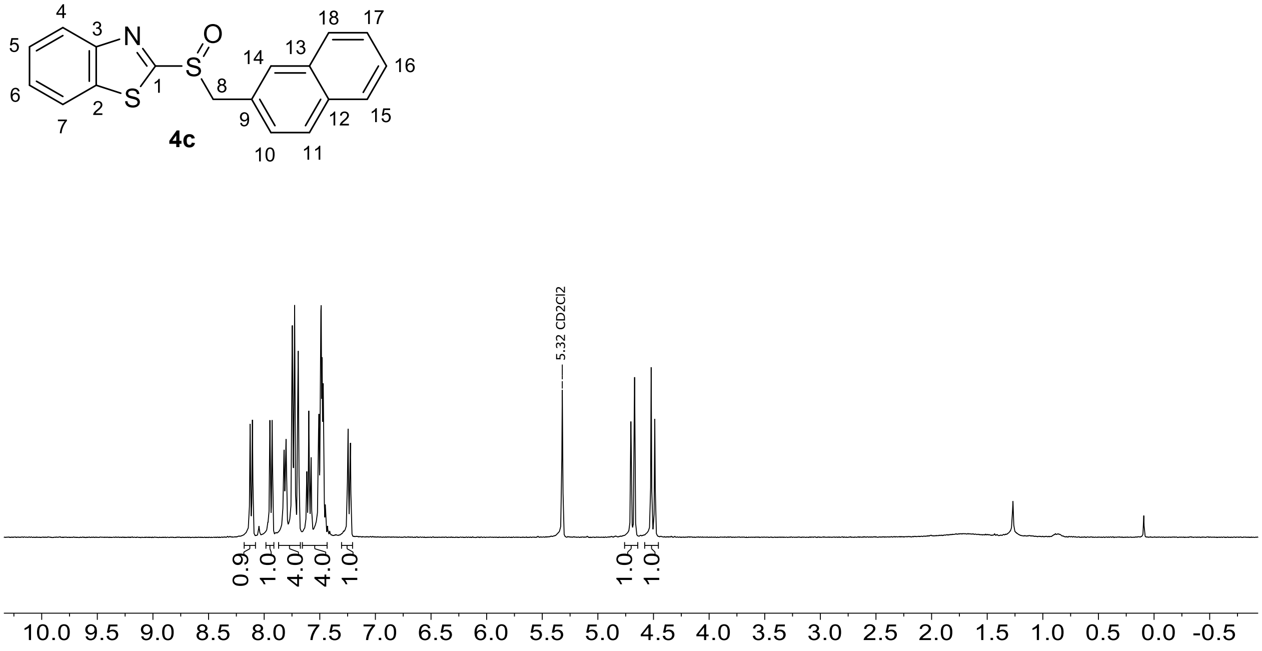

^{13}C{^{1}H} NMR (101 MHz, Methylene chloride-d_2, 298 K)

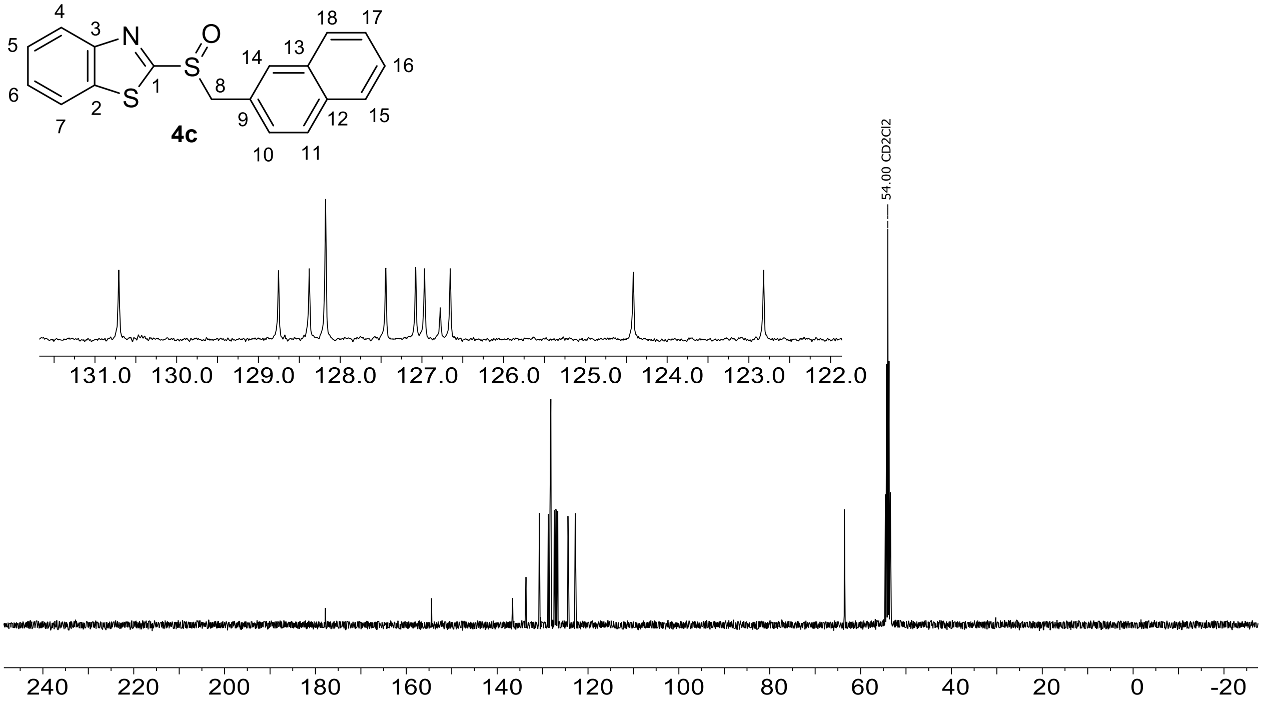

2-(Benzylsulfonyl)benzo[d]thiazole (2a)

^{1}H NMR (400 MHz, Methylene chloride-d_2, 298 K)

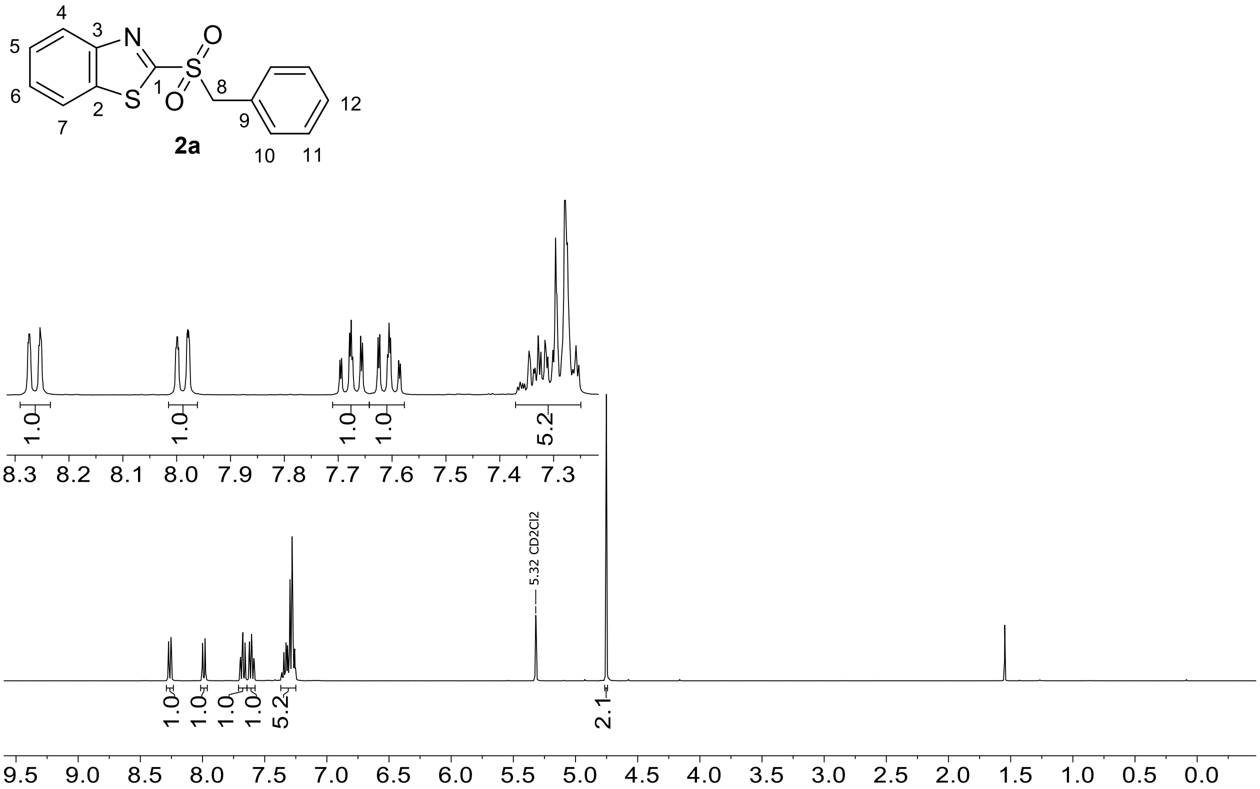

^{13}C{^{1}H} NMR (101 MHz, Methylene chloride-d_2, 298 K)

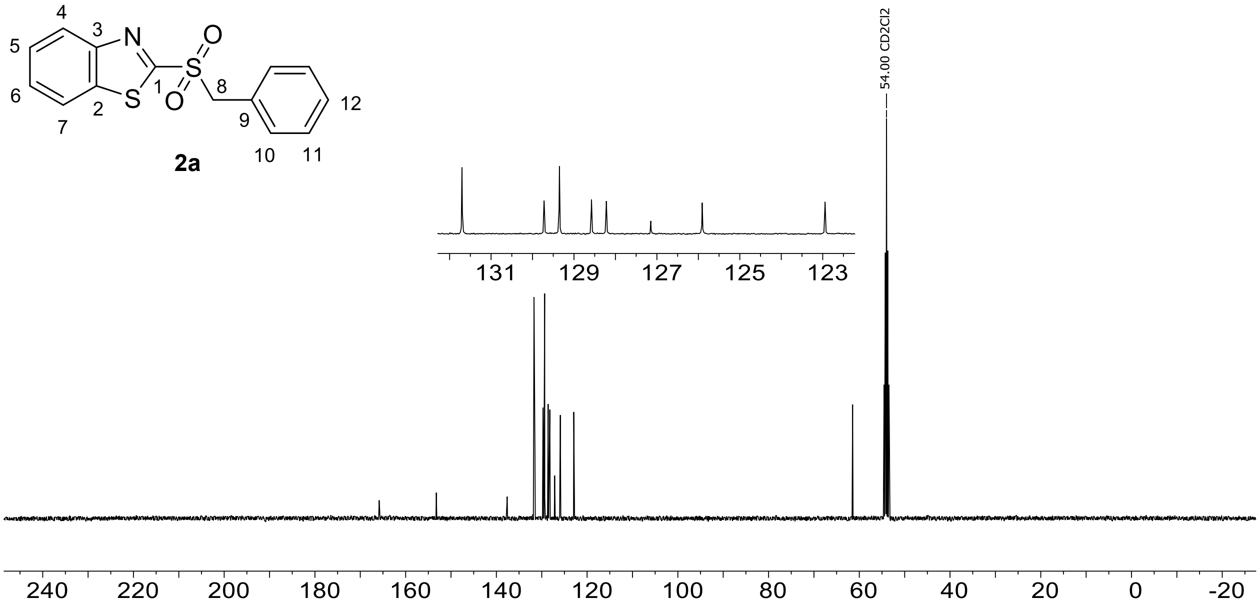

2-(Phenethylsulfonyl)benzo[d]thiazole (2b)

^{1}H NMR (600 MHz, Methylene chloride-d_2, 298 K)

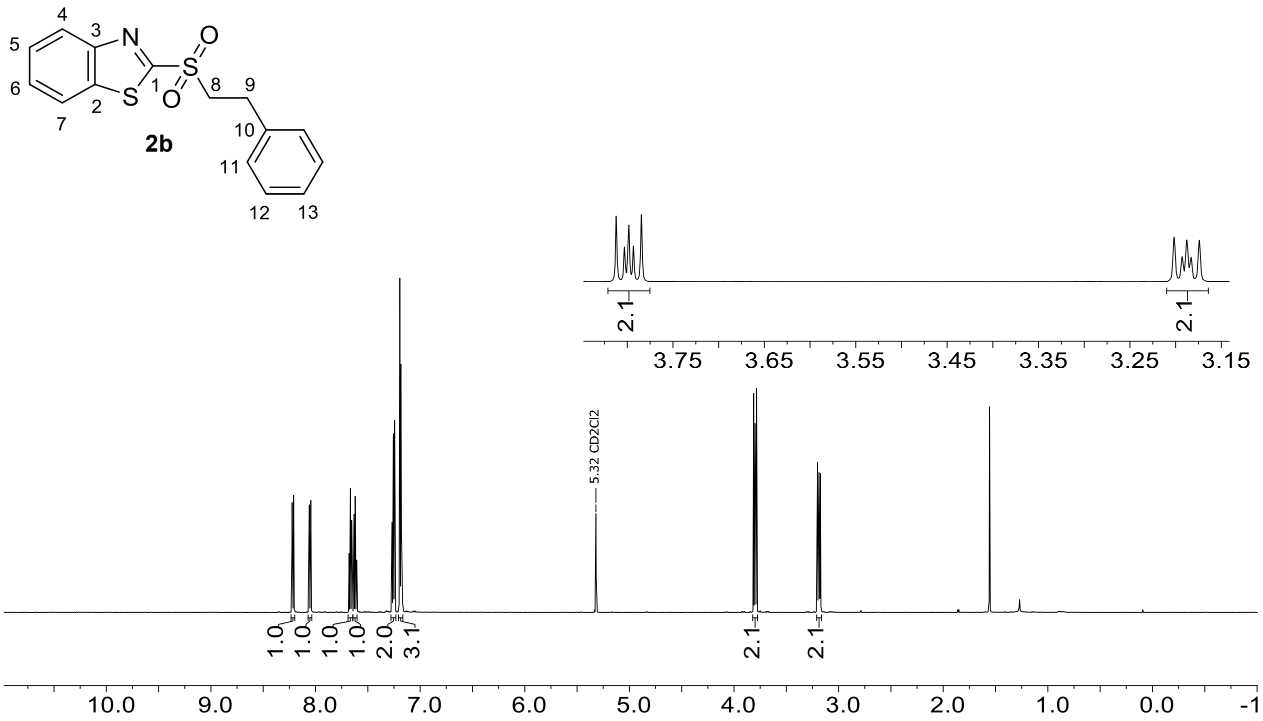

^{13}C{^{1}H} NMR (151 MHz, Methylene chloride-d_2, 298 K)

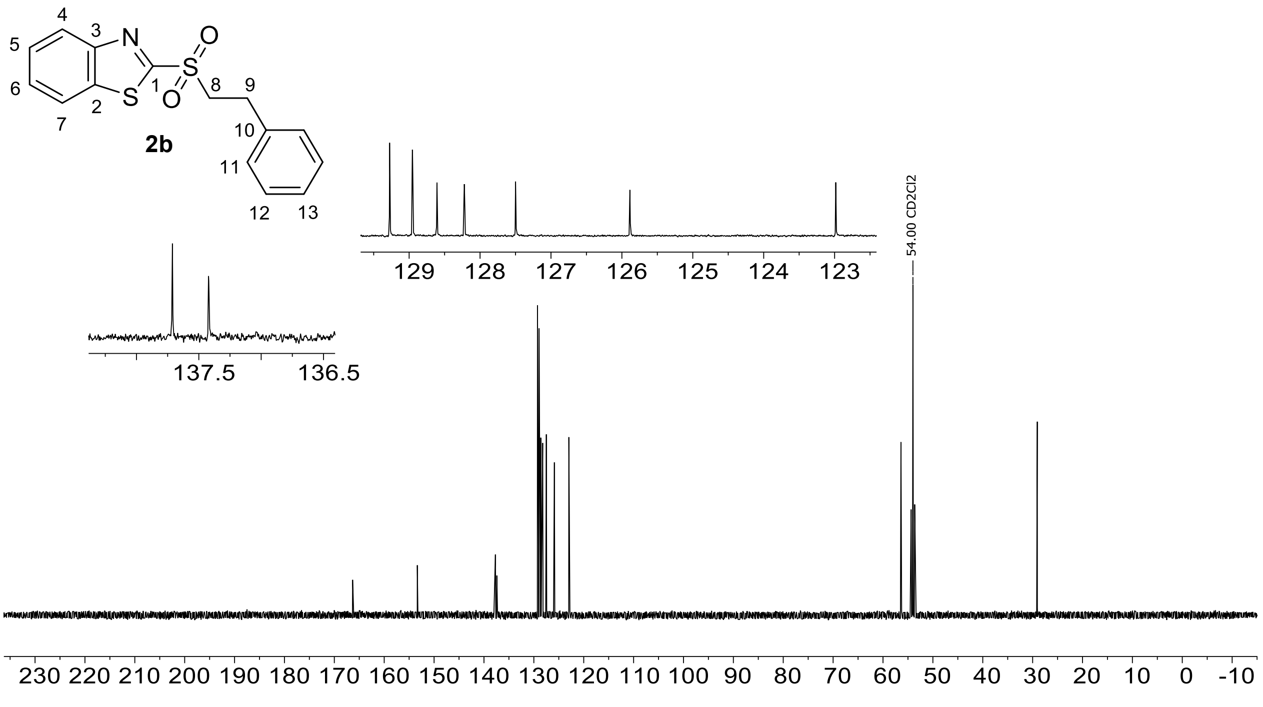

2-((Naphthalen-2-ylmethyl)sulfonyl)benzo[d]thiazole (2c)

^{1}H NMR (400 MHz, Methylene chloride-d_2, 298 K)

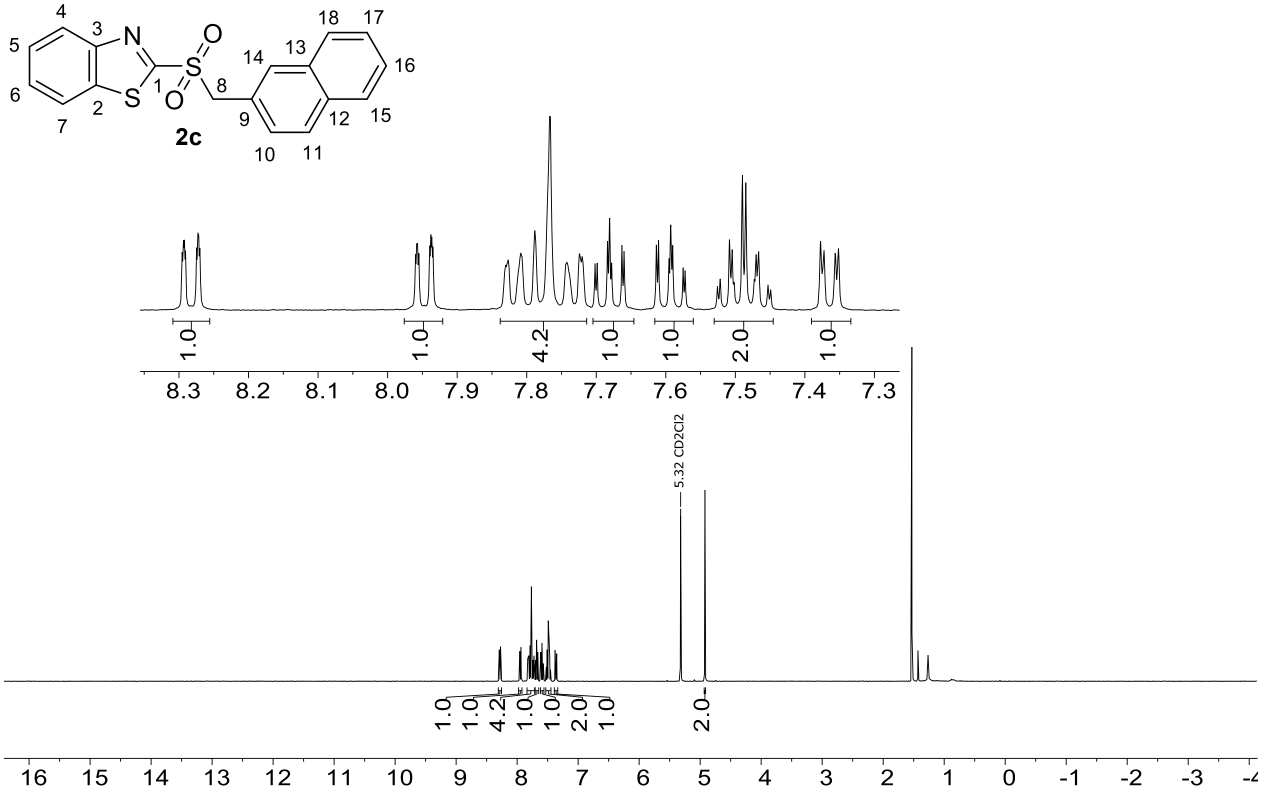

$^{13}C\{^{1}H\}$ NMR (101 MHz, Methylene chloride-d_2, 298 K)

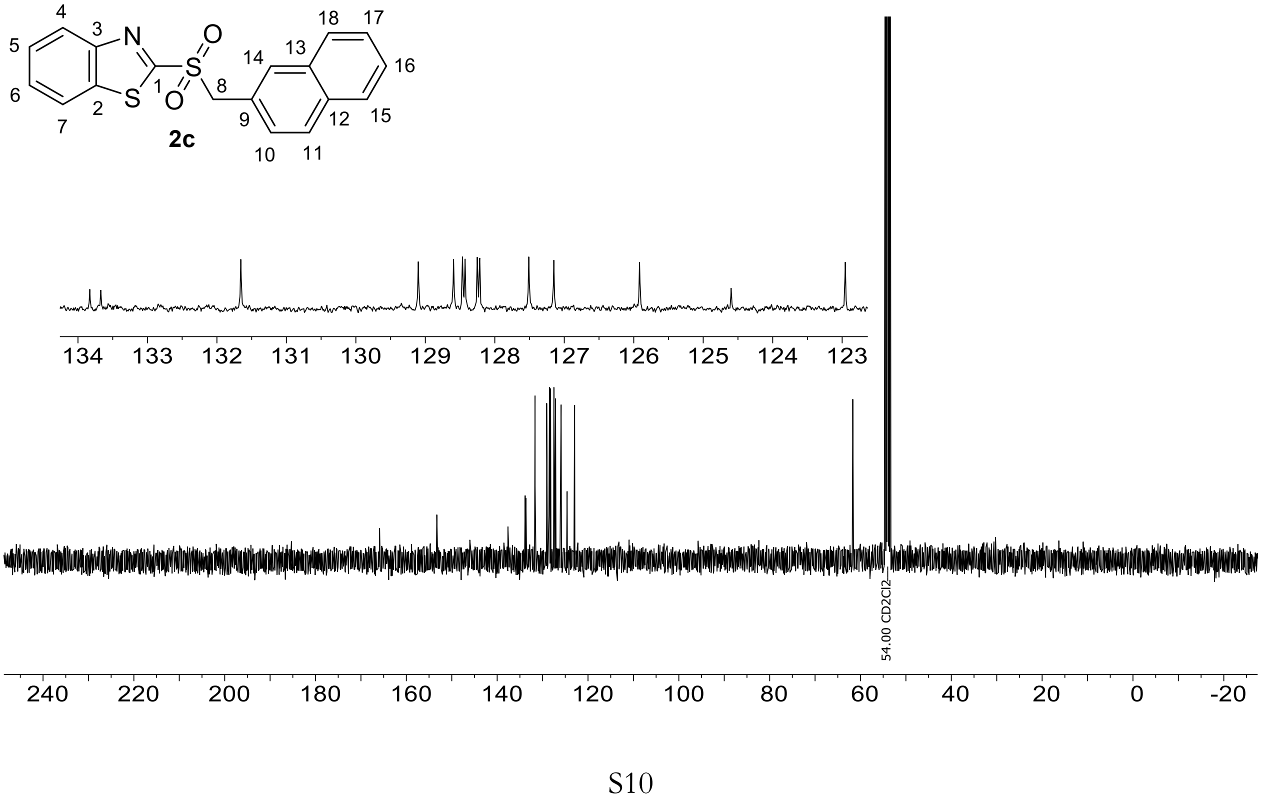

2-((1-Fluoro-2-phenylethyl)thio)benzo[d]thiazole (5b)

^{1}H NMR (600 MHz, Methylene chloride-d_2, 298 K)

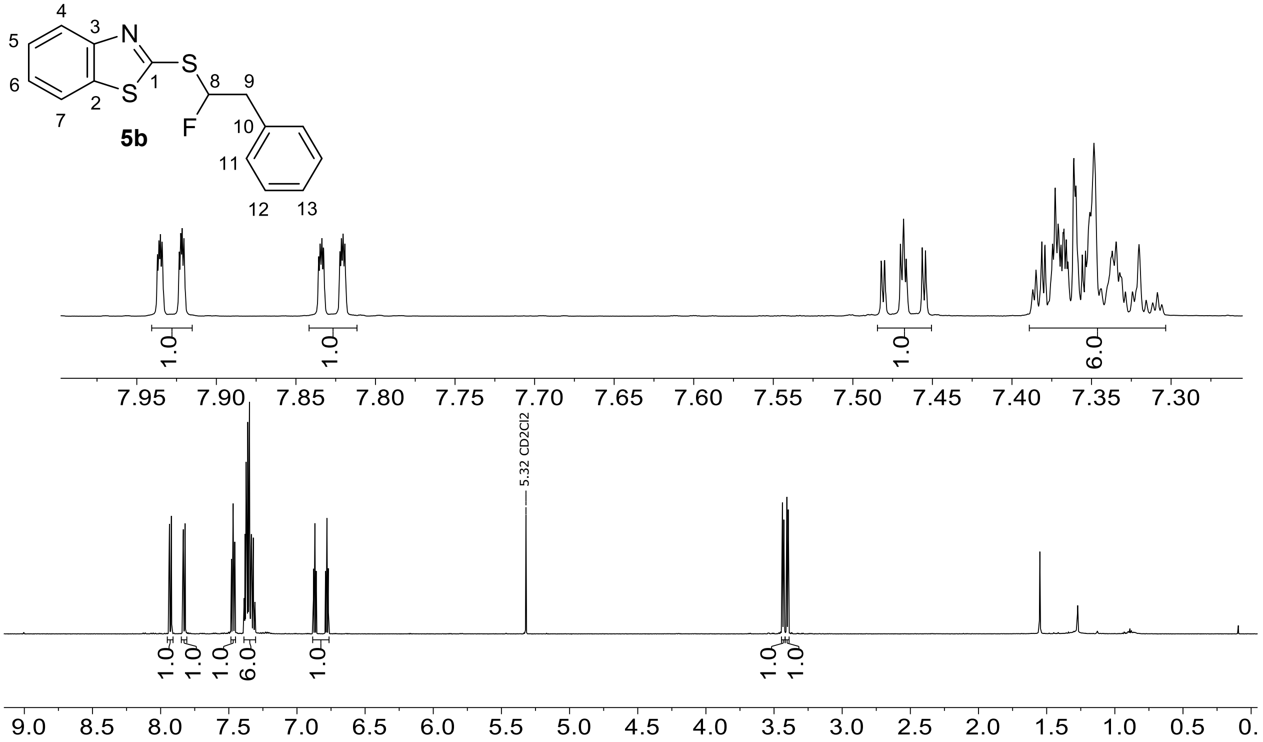

^{13}C{^{1}H} NMR (151 MHz, Methylene chloride-d_2, 298 K)

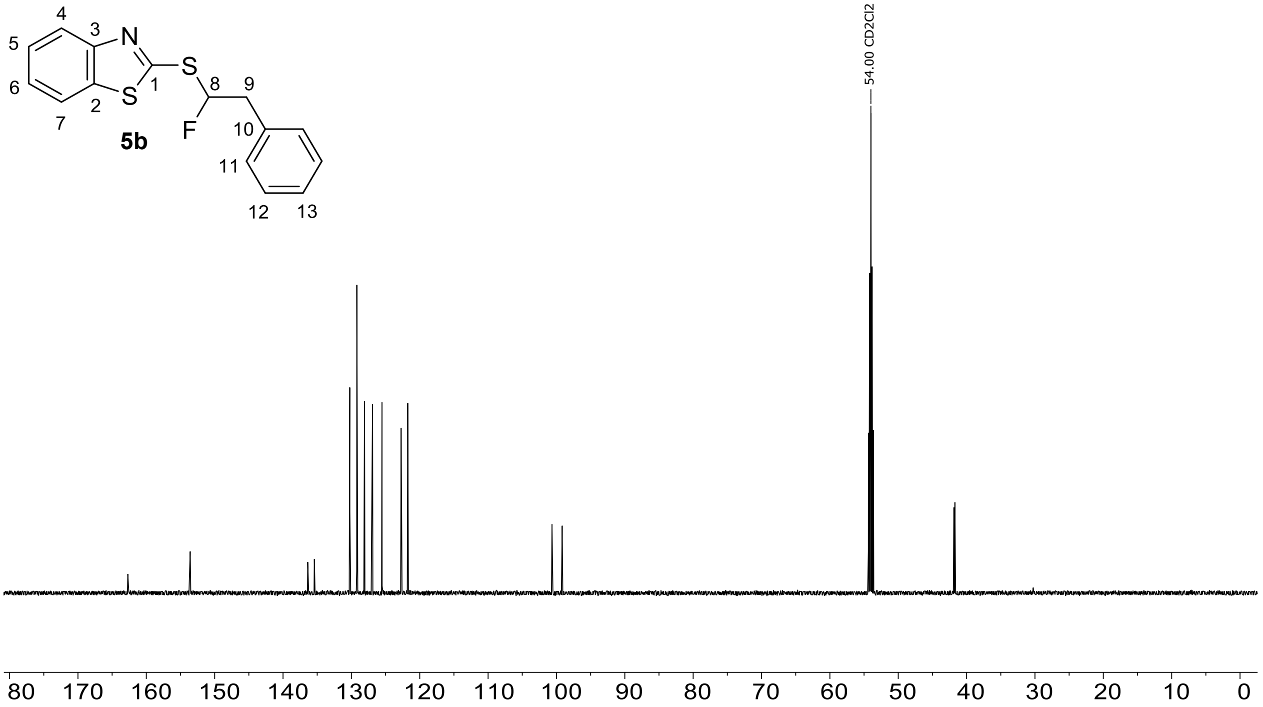

^{19}F NMR (564 MHz, Methylene chloride-d_2, 298 K)

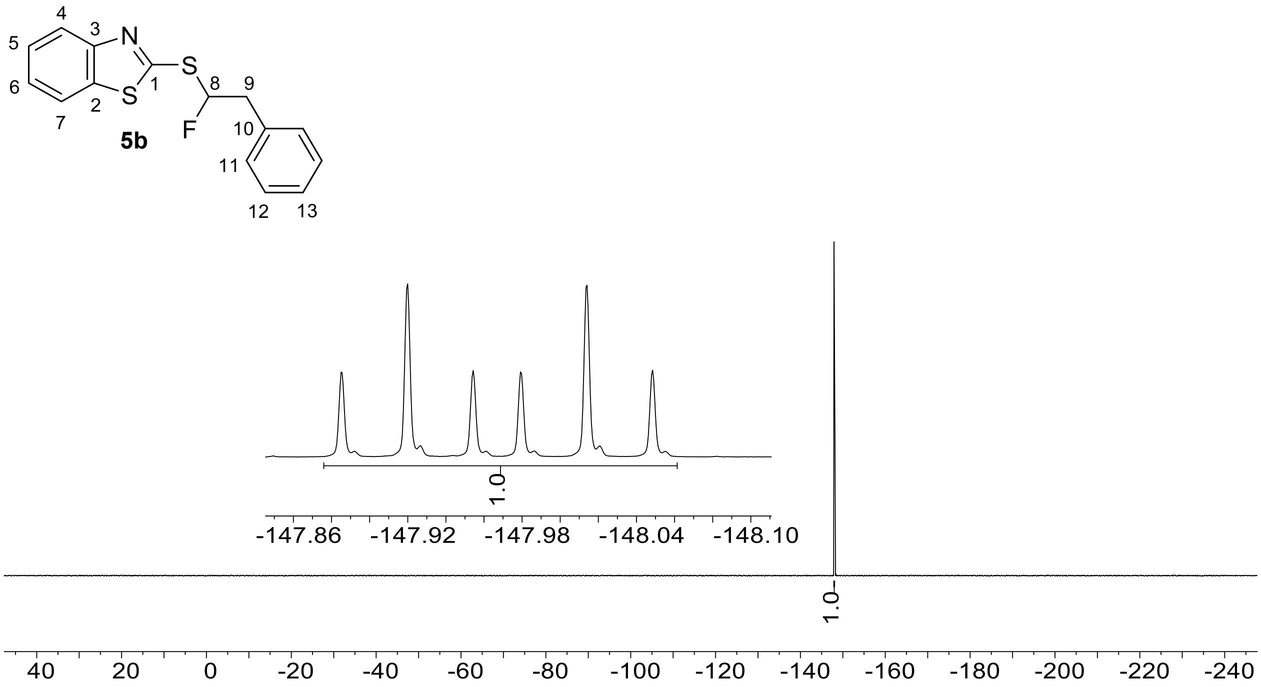

2-((Fluoro(phenyl)methyl)sulfinyl)benzo[d]thiazole (6a)

^{1}H NMR (600 MHz, Methylene chloride-d_2, 298 K)

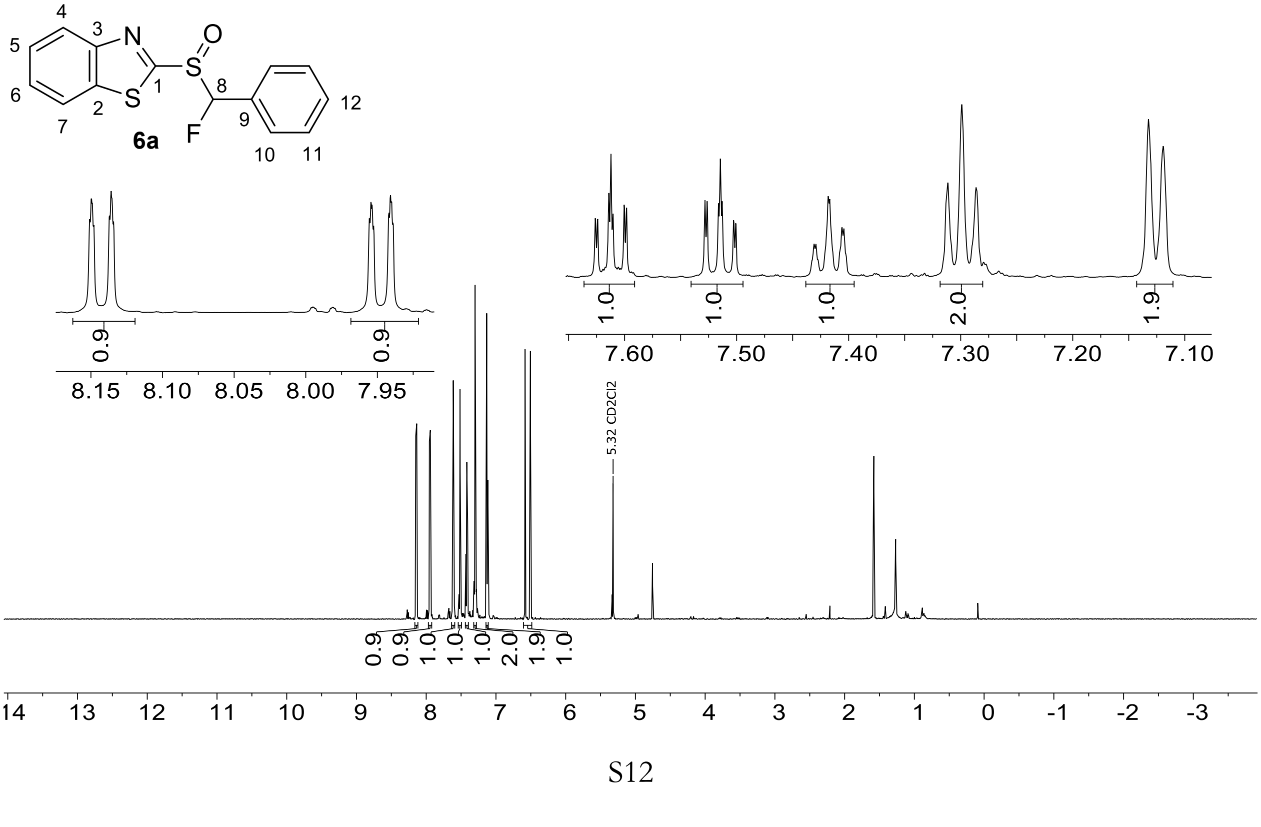

$^{13}C\{^1H\}$ NMR (151 MHz, Methylene chloride-d_2, 298 K)

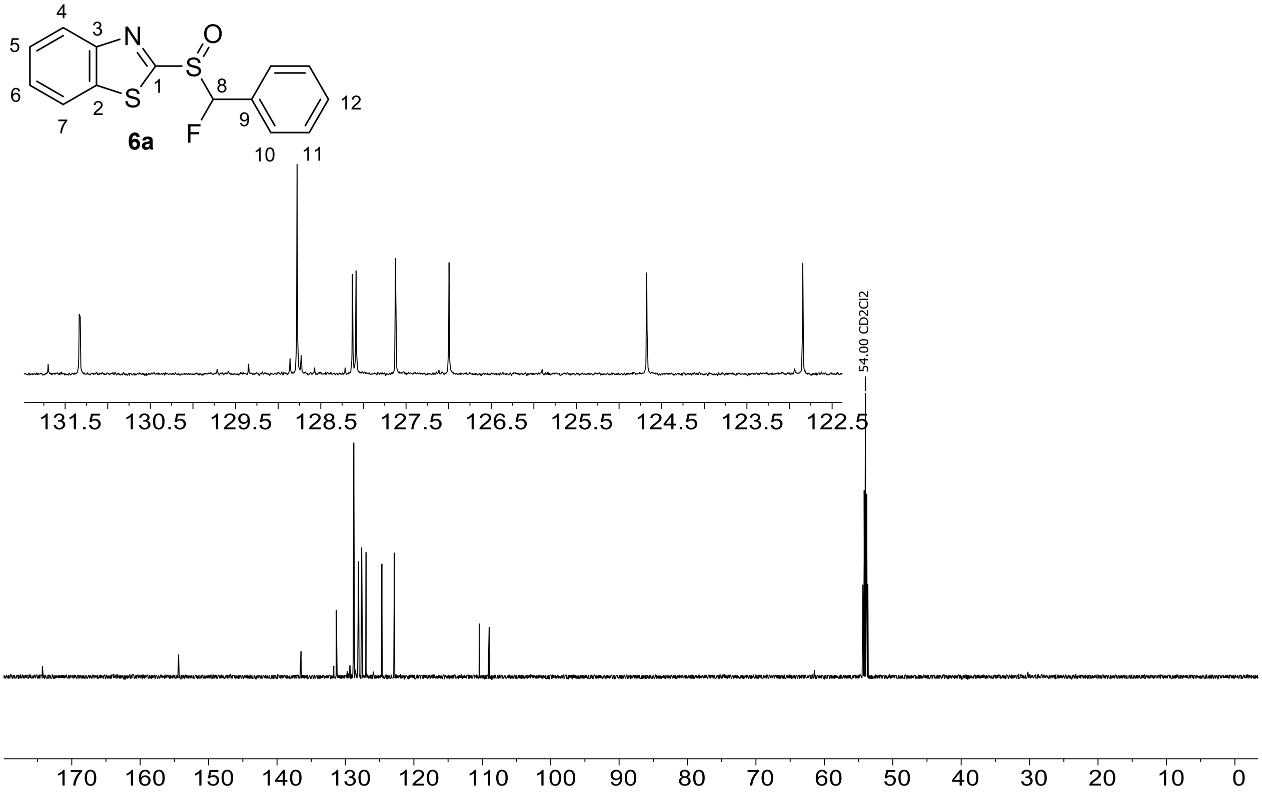

^{19}F NMR (564 MHz, Methylene chloride-d_2, 298 K)

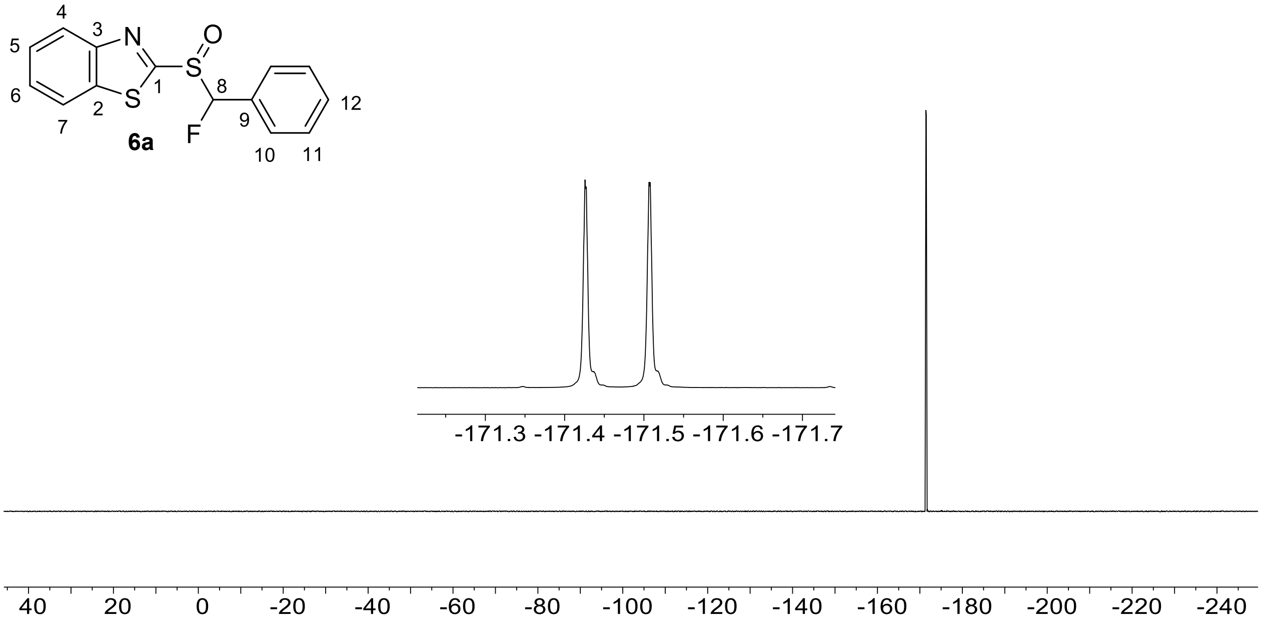

2-((Fluoro(phenyl)methyl)sulfinyl)benzo[d]thiazole (6b)

^{1}H NMR (600 MHz, Methylene chloride-d_2, 298 K)

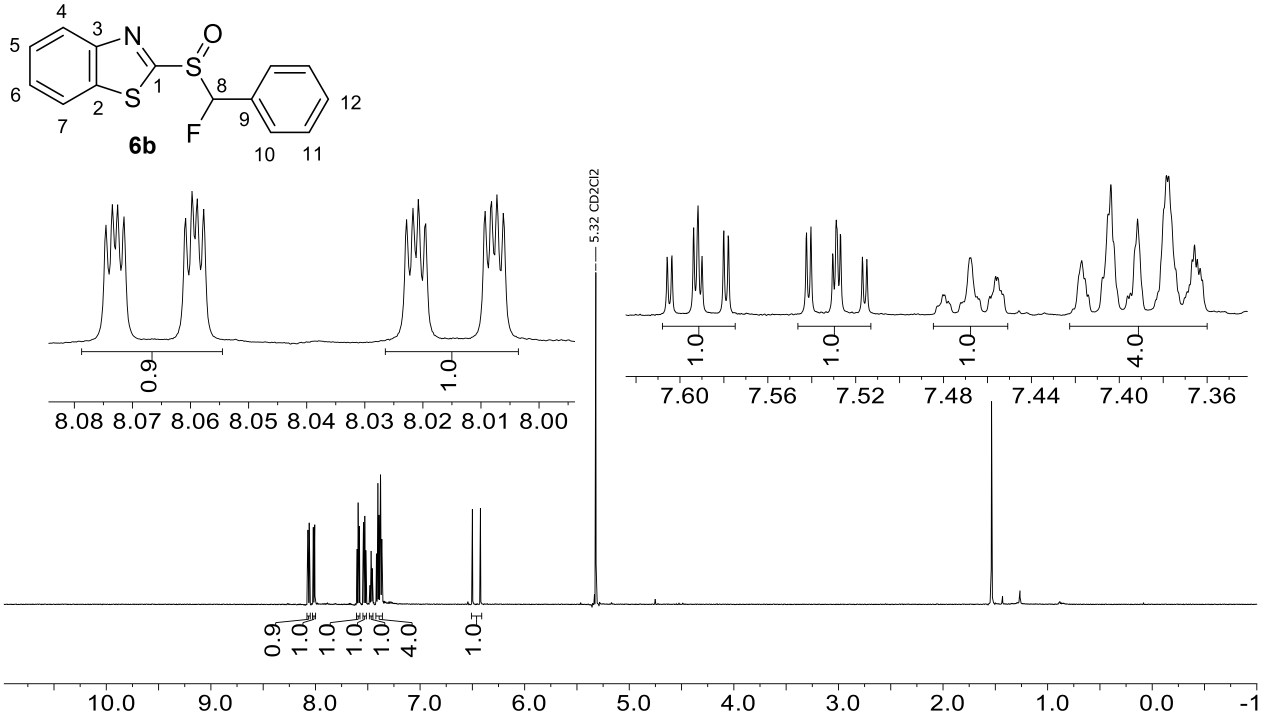

$^{13}C\{^{1}H\}$ NMR (151 MHz, Methylene chloride-d_2, 298 K)

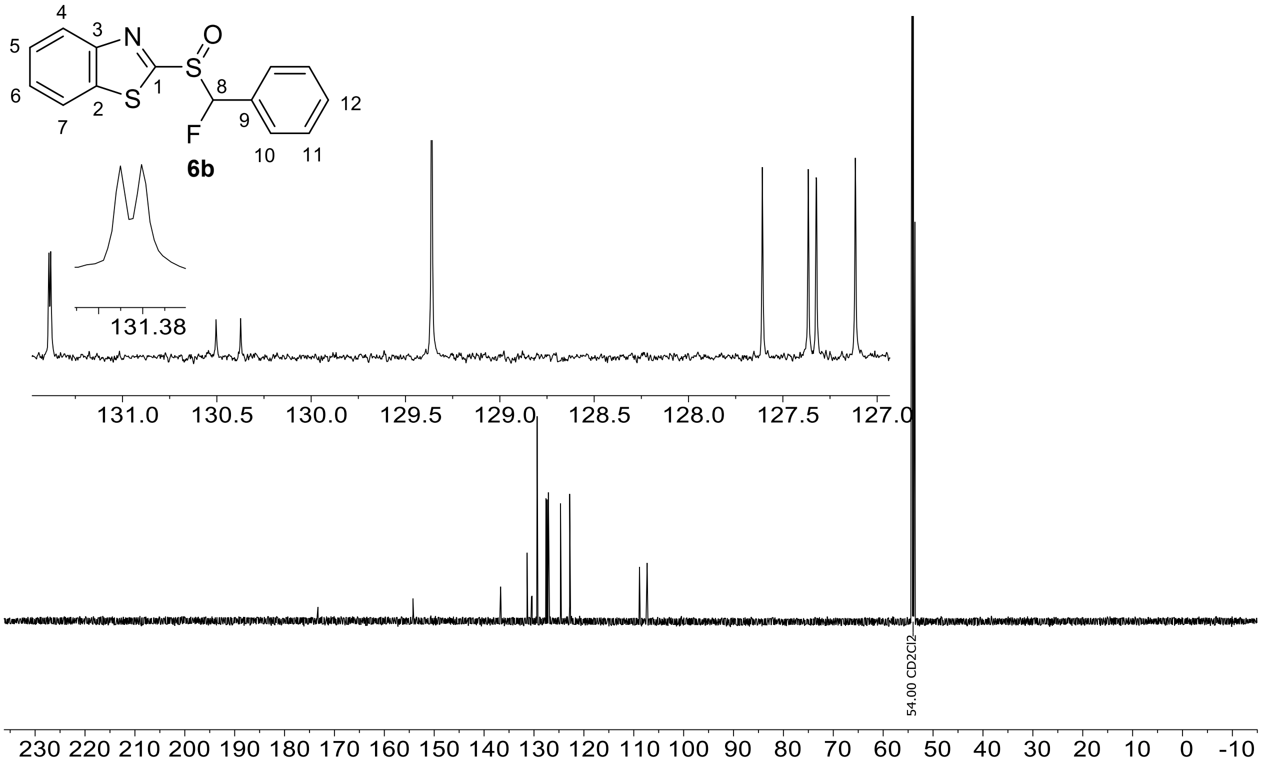

^{19}F NMR (564 MHz, Methylene chloride-d_2, 298 K)

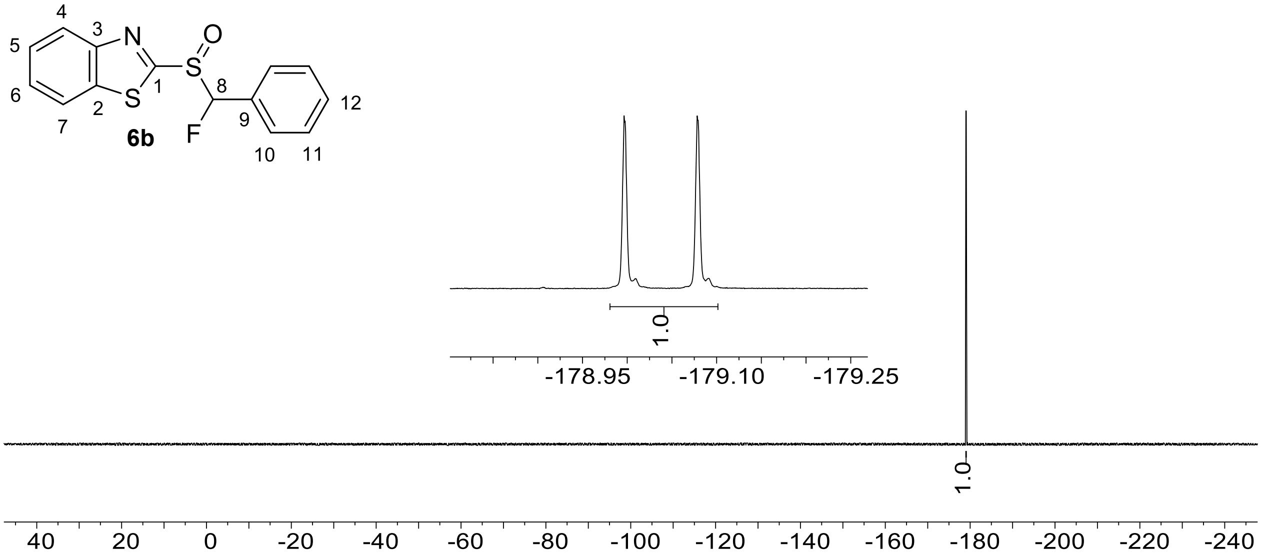

2-((1-Fluoro-2-phenylethyl)sulfinyl)benzo[d]thiazole (7a)

^{1}H NMR (600 MHz, Methylene chloride-d_2, 298 K)

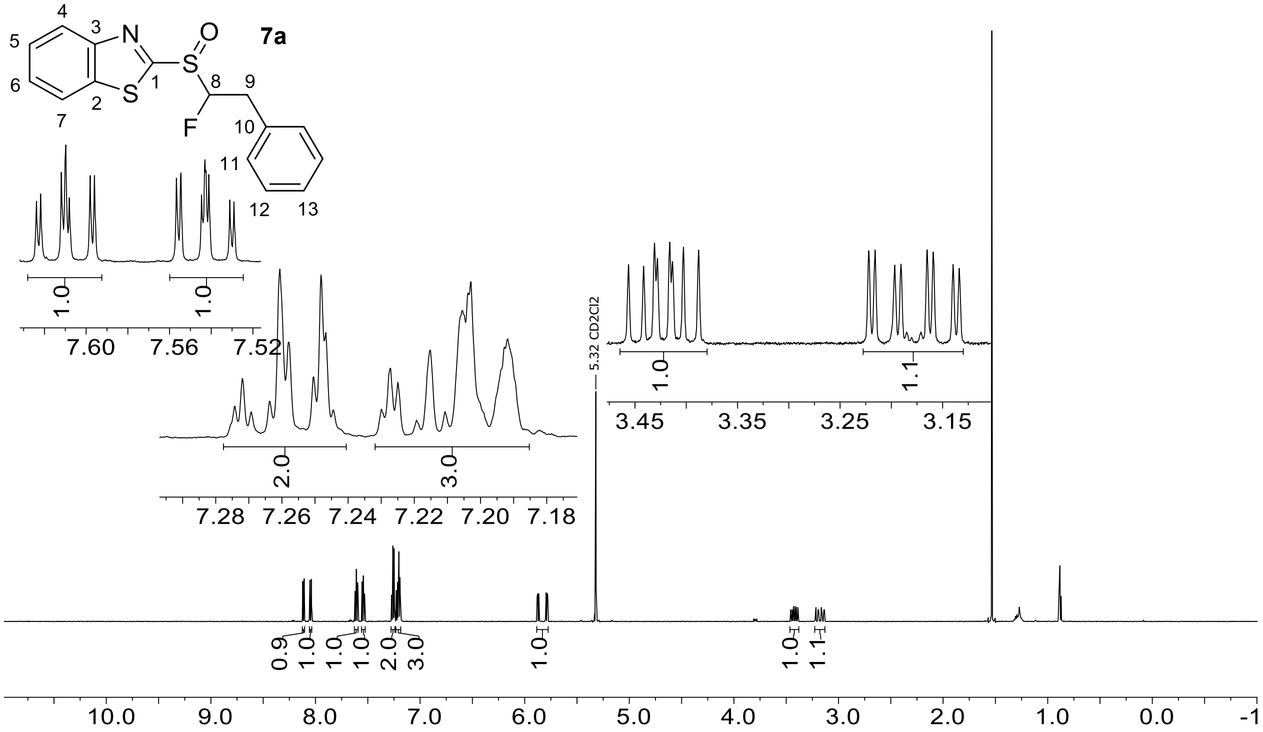

$^{13}C\{^{1}H\}$ NMR (151 MHz, Methylene chloride-d_2, 298 K)

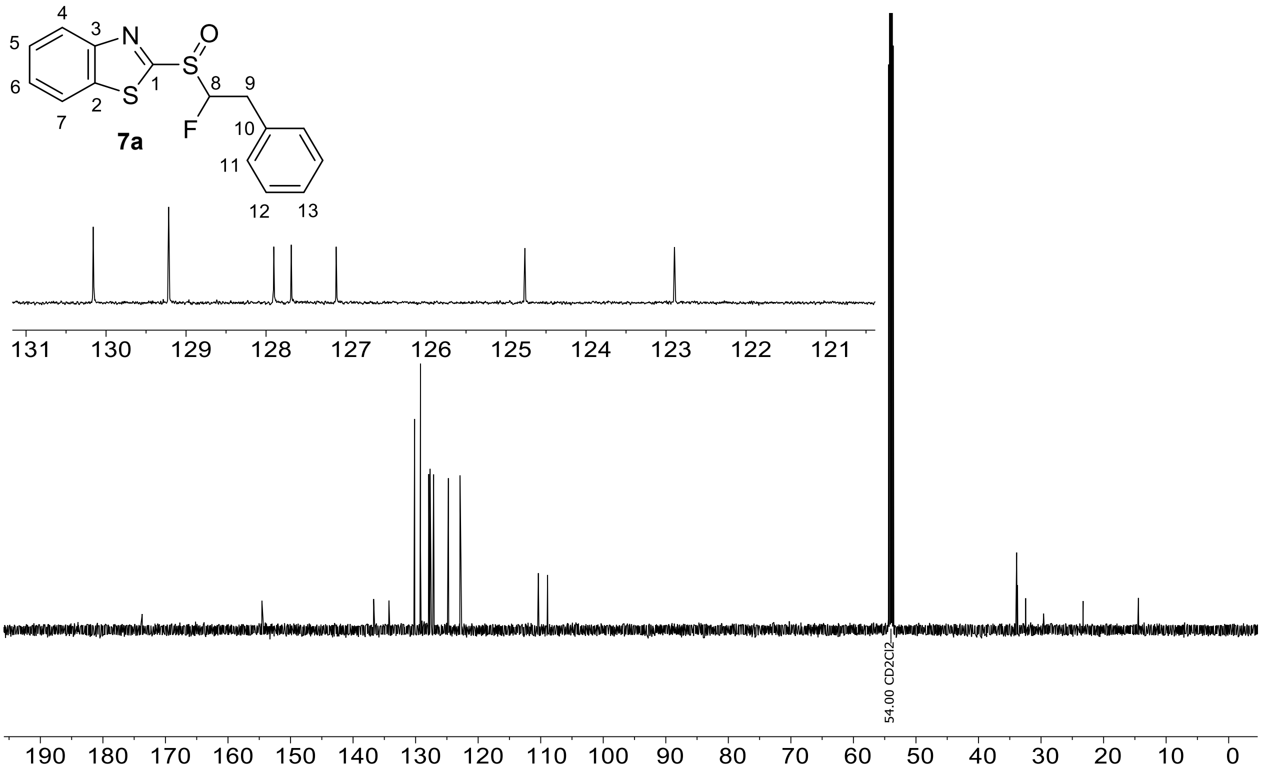

^{19}F NMR (564 MHz, Methylene chloride-d_2, 298 K)

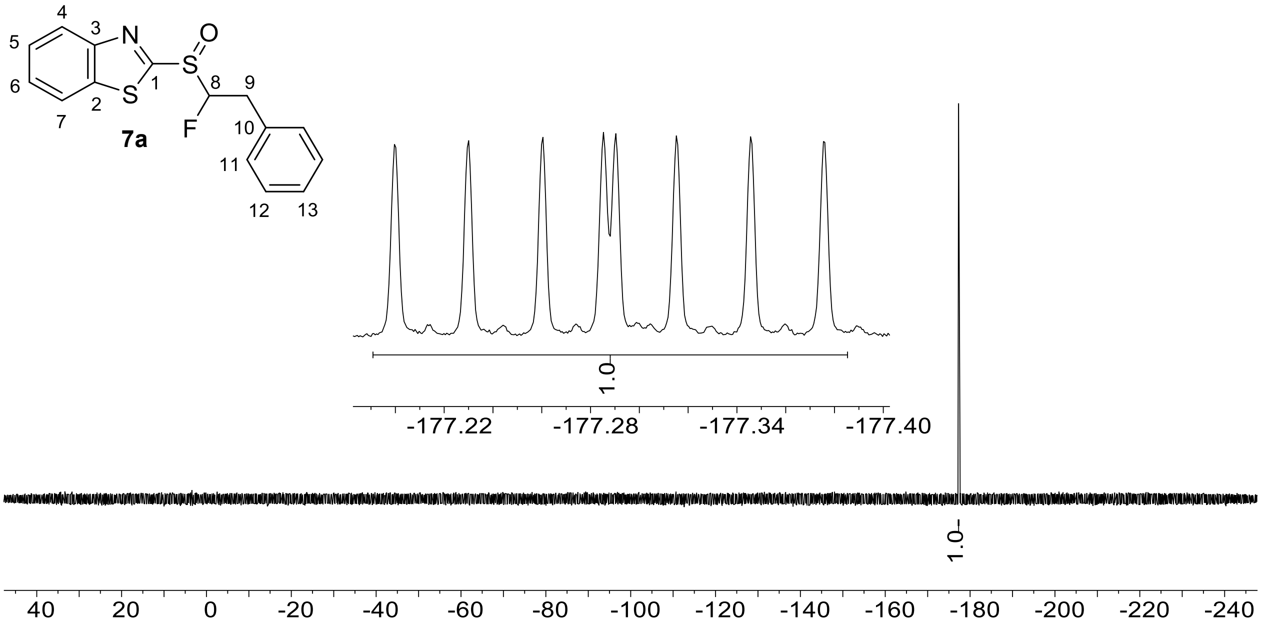

2-((1-Fluoro-2-phenylethyl)sulfinyl)benzo[d]thiazole (7b)

^{1}H NMR (600 MHz, Methylene chloride-d_2, 298 K)

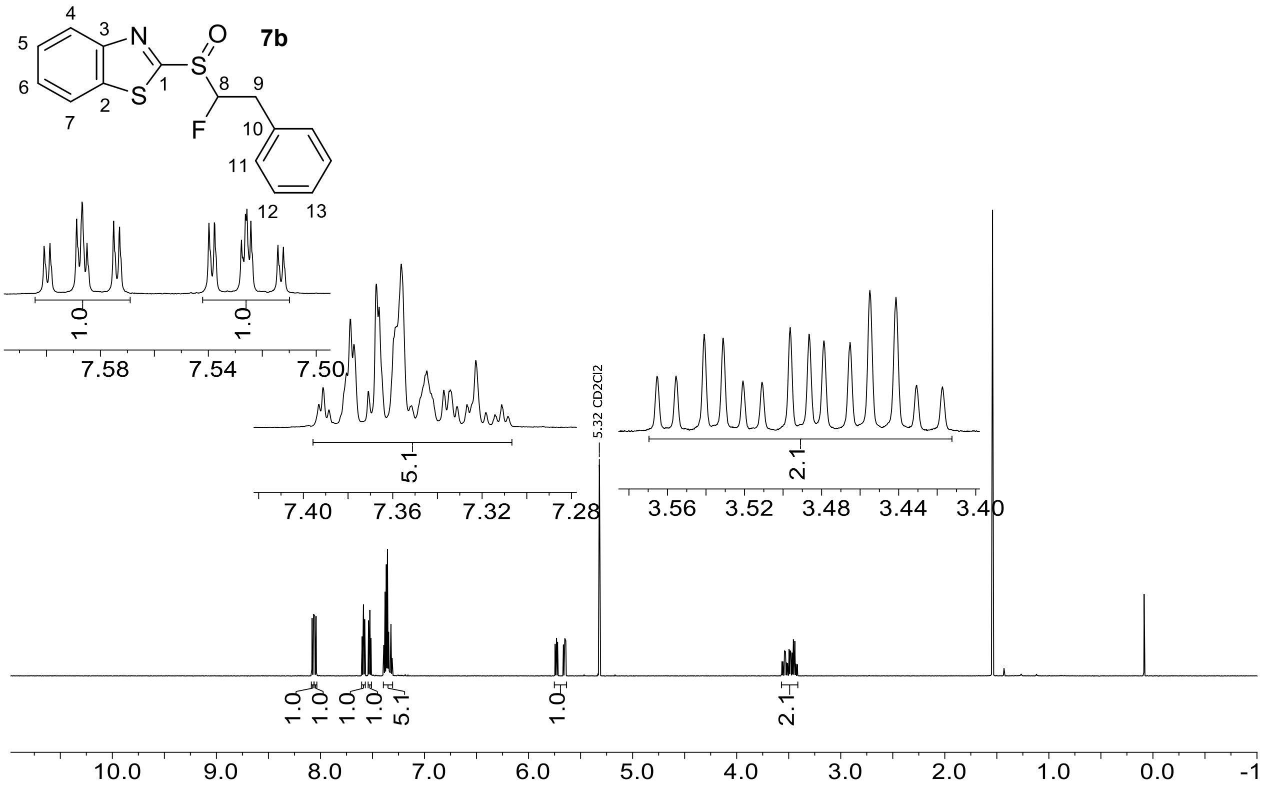

^{13}C{^{1}H} NMR (151 MHz, Methylene chloride-d_2, 298 K)

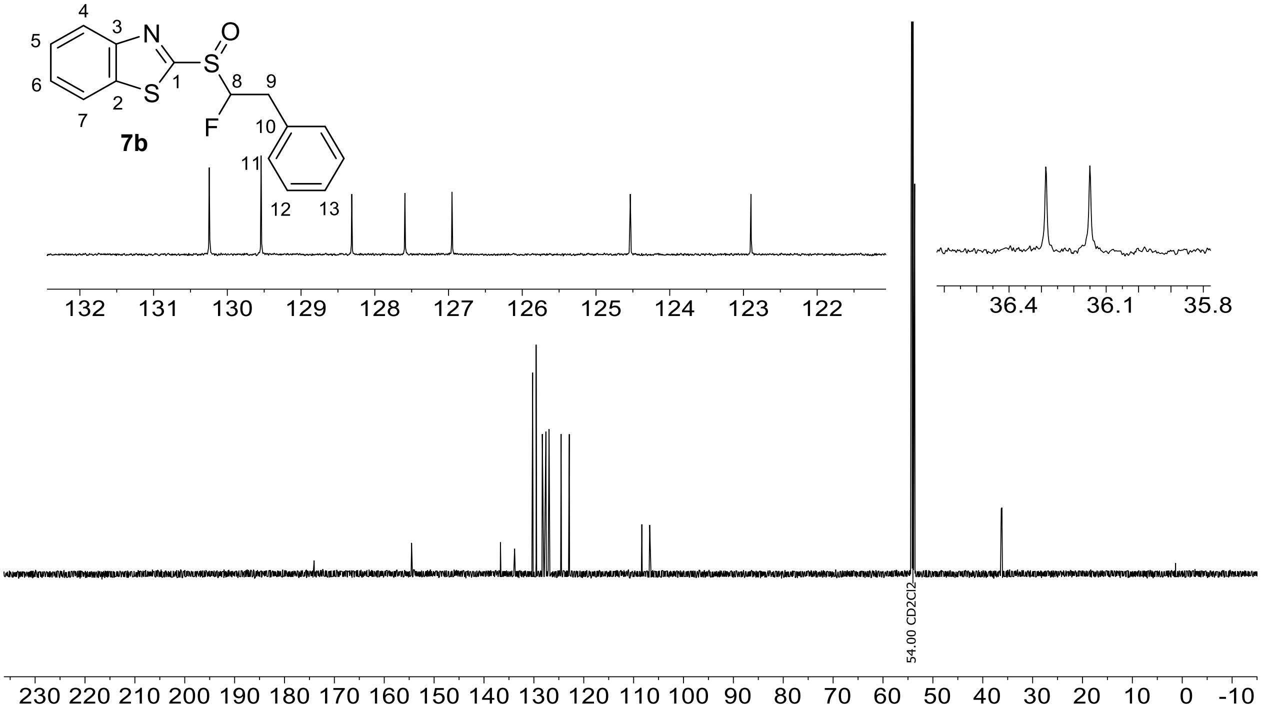

^{19}F NMR (564 MHz, Methylene chloride-d_2, 298 K)

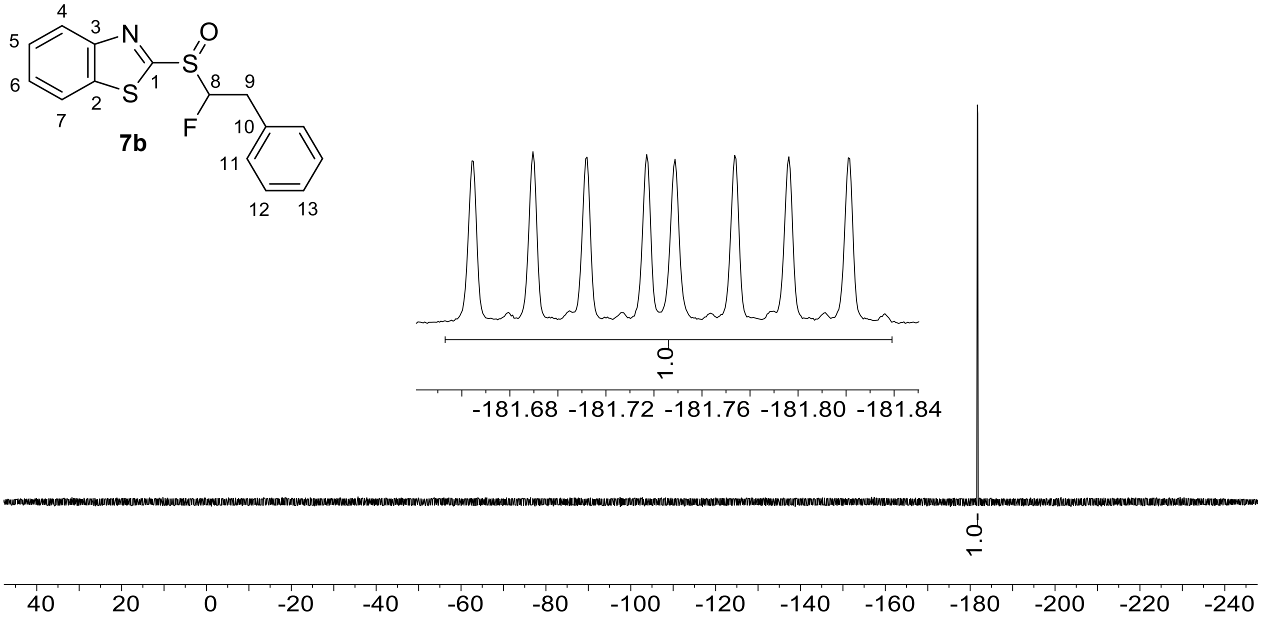

2-((Fluoro(naphthalen-2-yl)methyl)sulfinyl)benzo[d]thiazole (8a)

^{1}H NMR (600 MHz, Methylene chloride-d_2, 298 K)

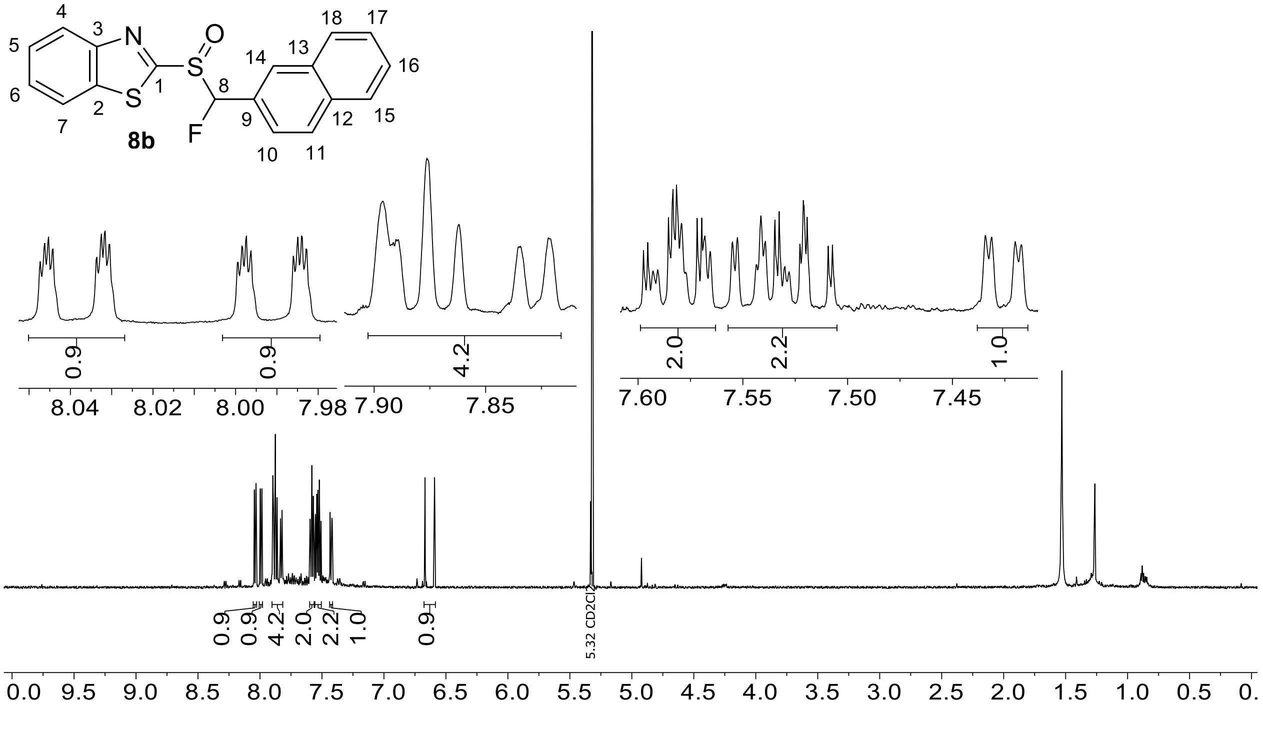

$^{13}C\{^{1}H\}$ NMR (151 MHz, Methylene chloride-d_2, 298 K)

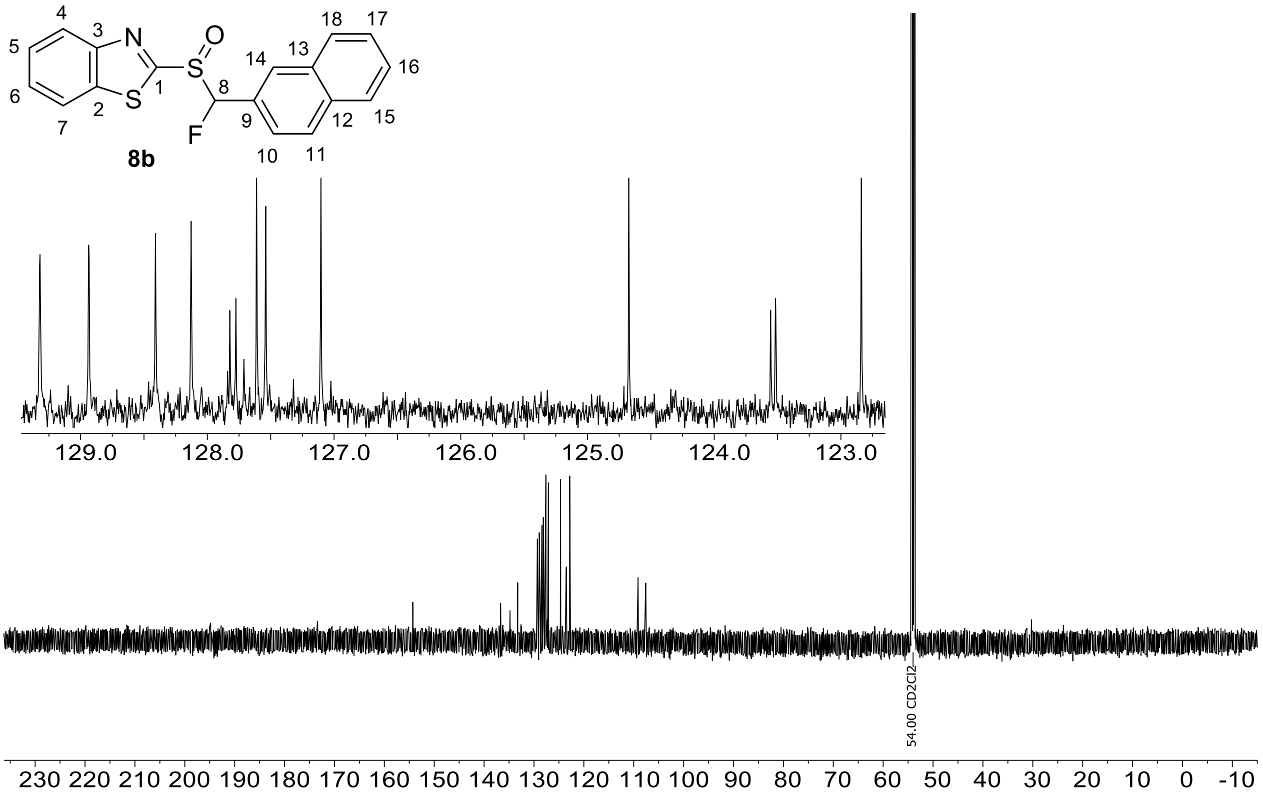

^{19}F NMR (564 MHz, Methylene chloride-d_2, 298 K)

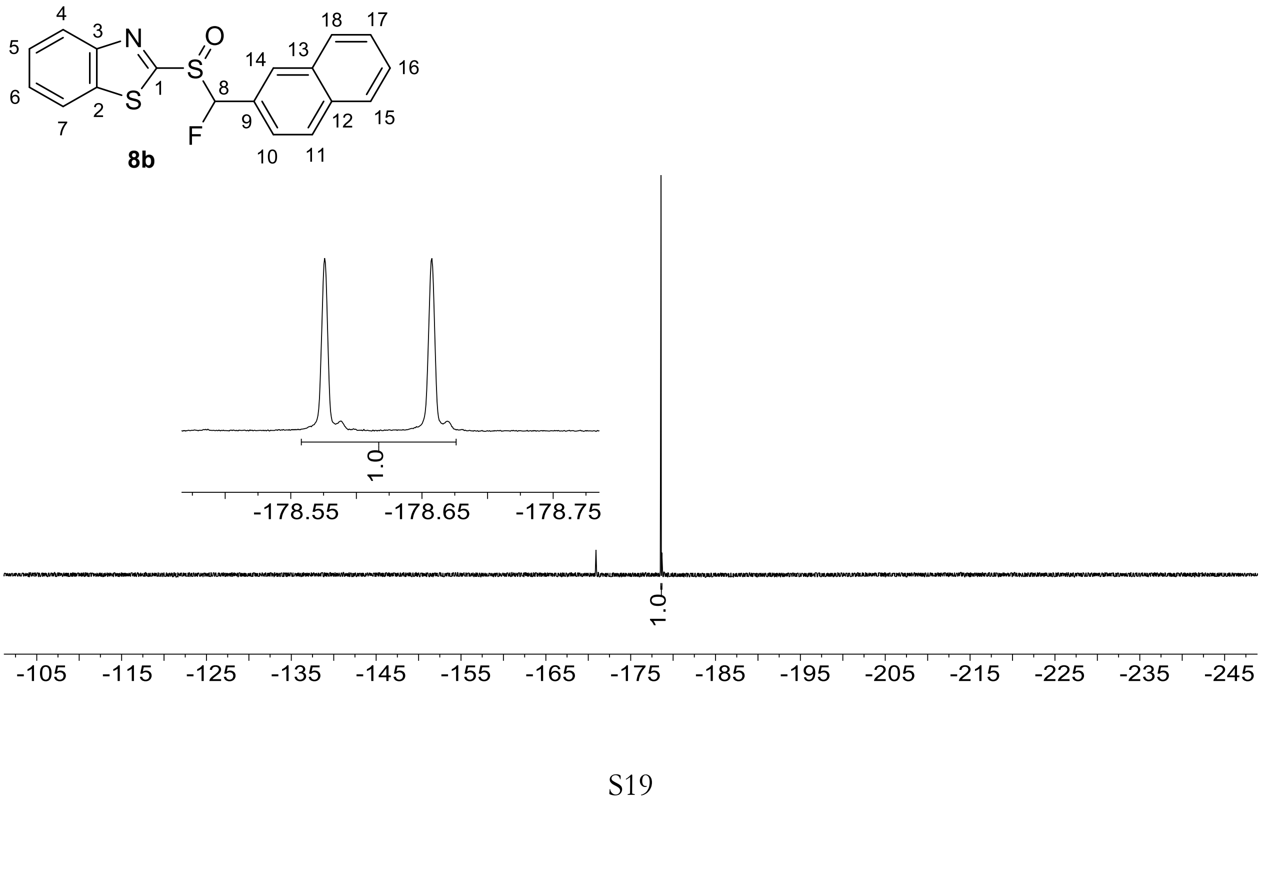

2-((Fluoro(phenyl)methyl)sulfonyl)benzo[d]thiazole (3a)

^{1}H NMR (600 MHz, Methylene chloride-d_2, 298 K)

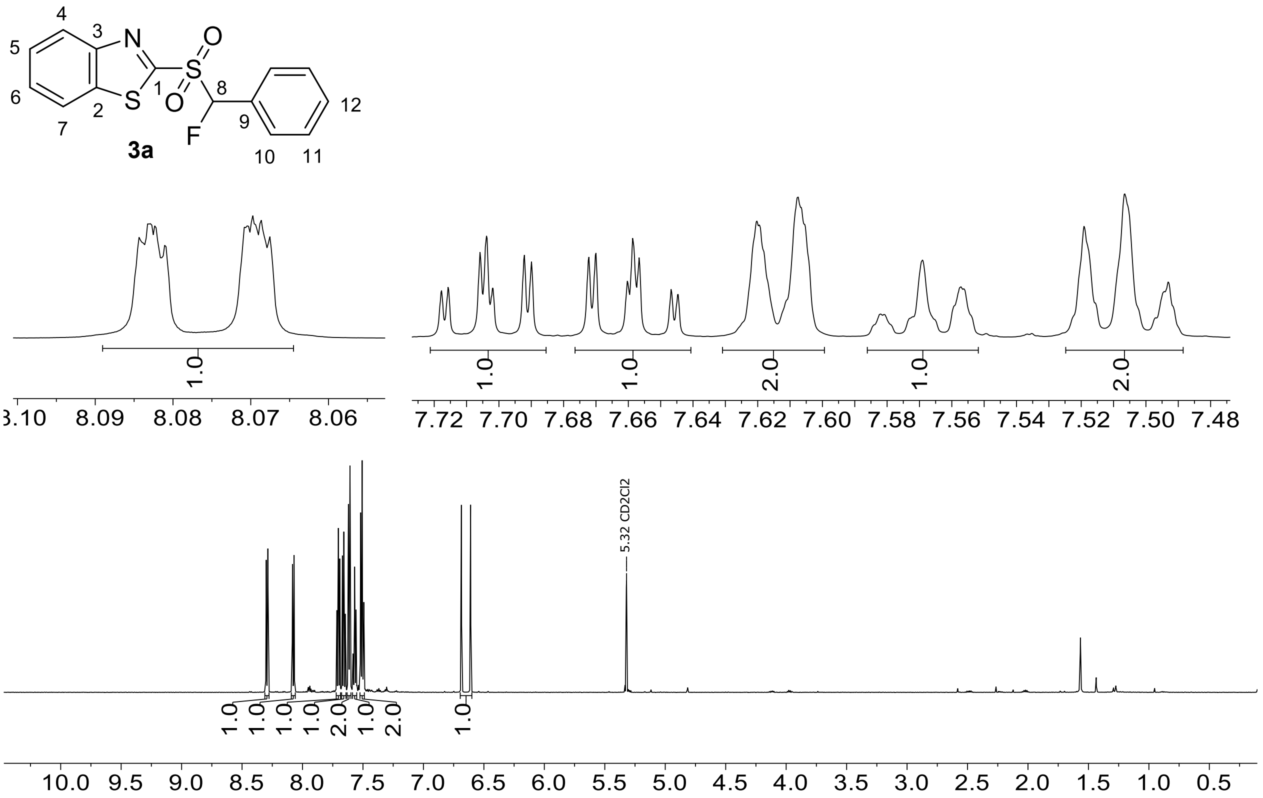

^{13}C{^{1}H} NMR (151 MHz, Methylene chloride-d_2, 298 K)

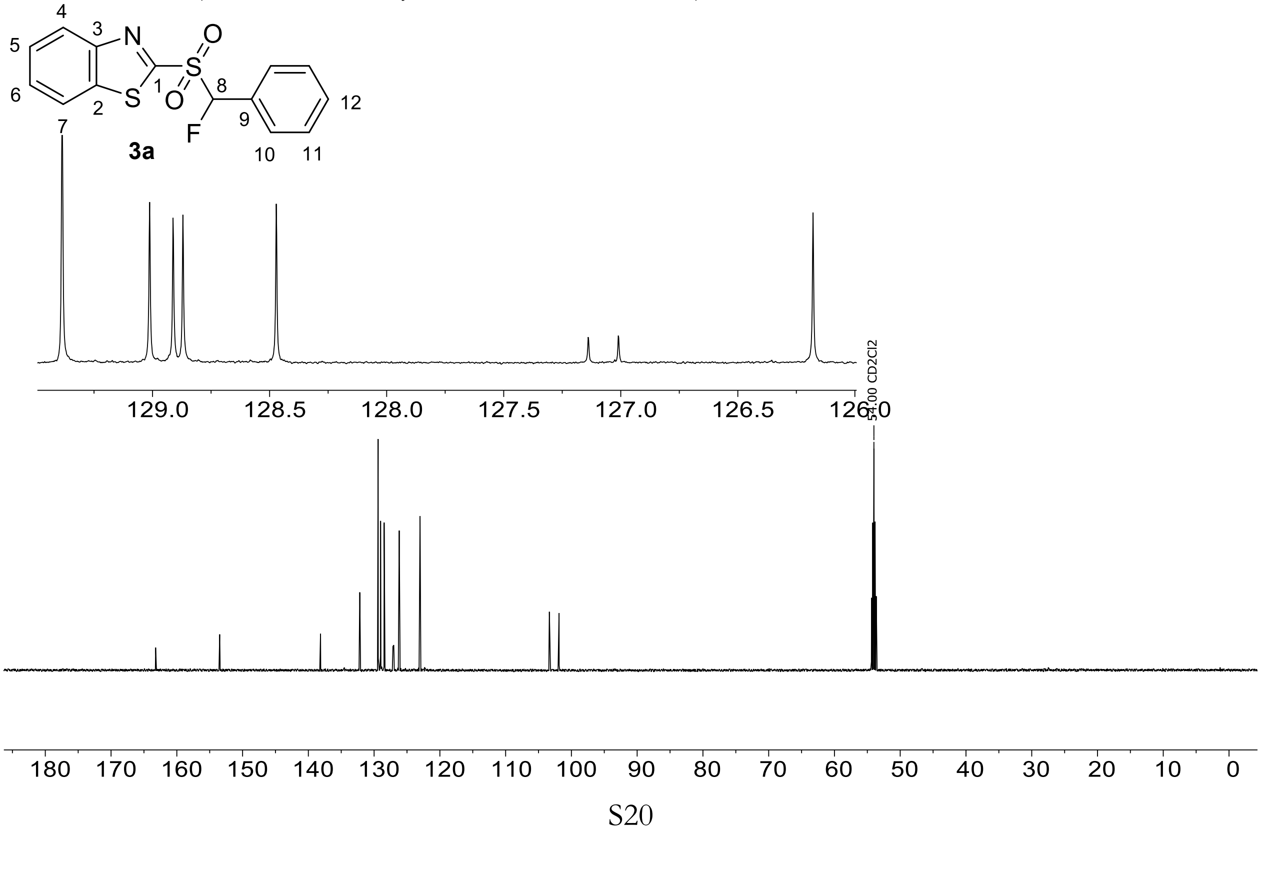

^{19}F NMR (564 MHz, Methylene chloride-d_2, 298 K)

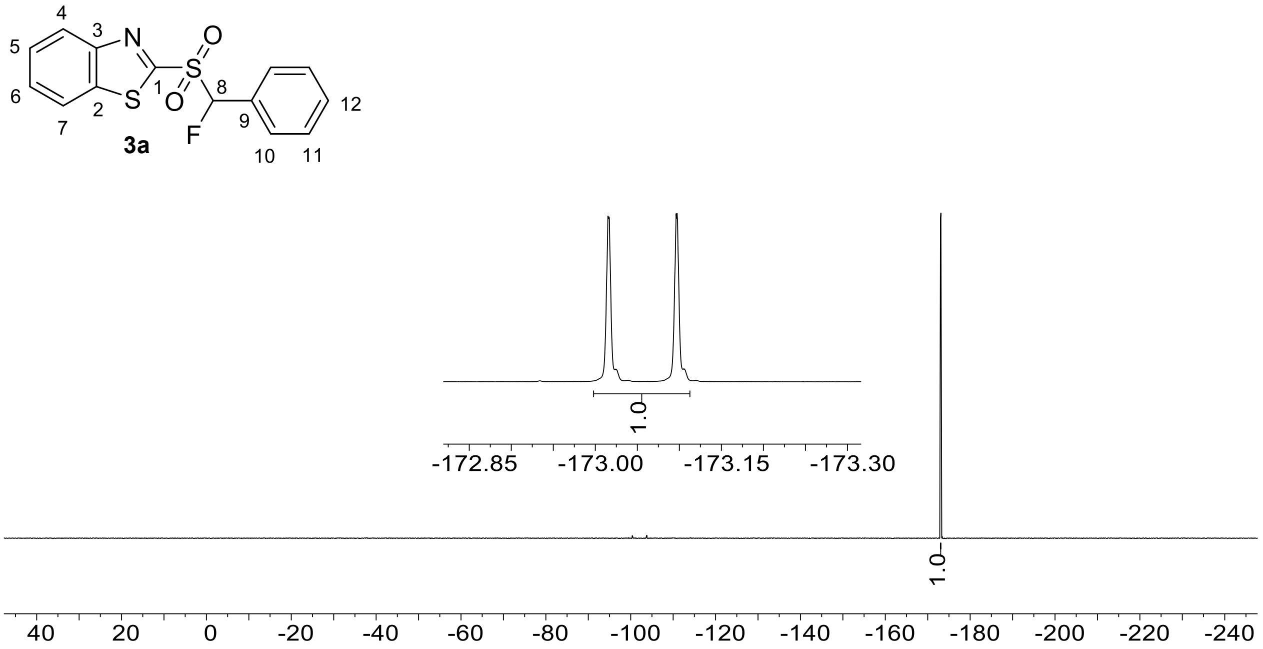

2-((1-Fluoro-2-phenylethyl)sulfonyl)benzo[d]thiazole (3b)

^{1}H NMR (600 MHz, Methylene chloride-d_2, 298 K)

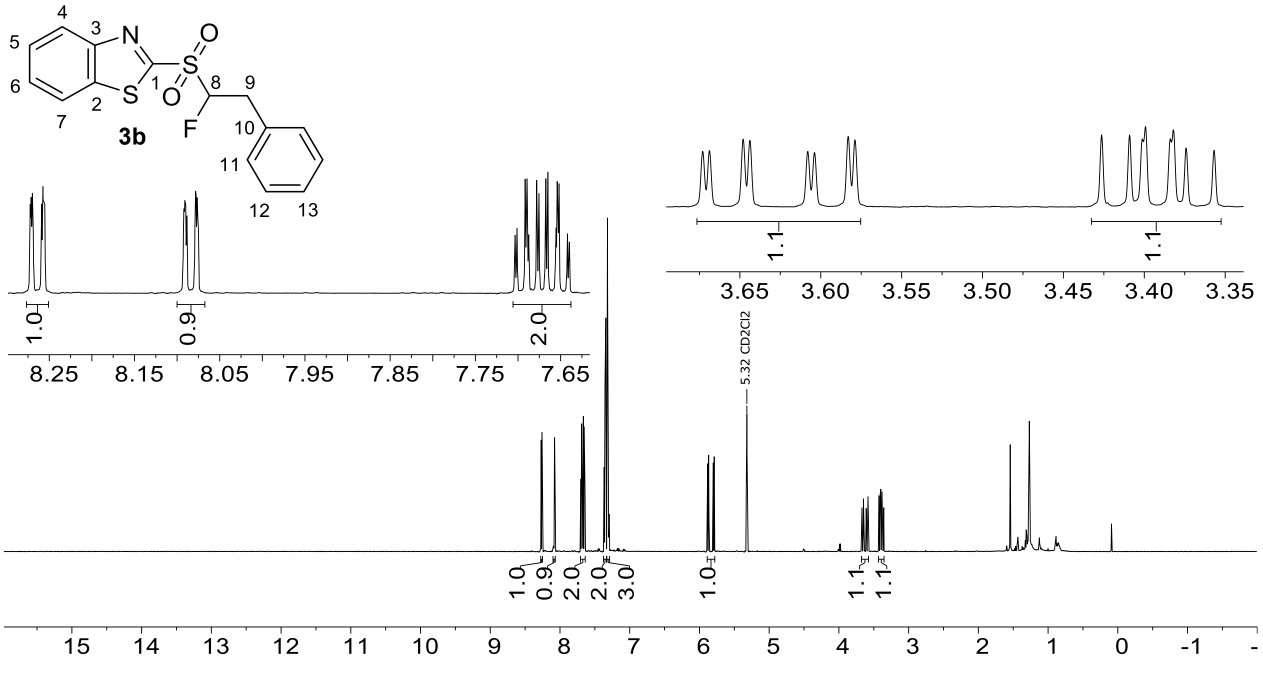

$^{13}C\{^{1}H\}$ NMR (151 MHz, Methylene chloride-d_2, 298 K)

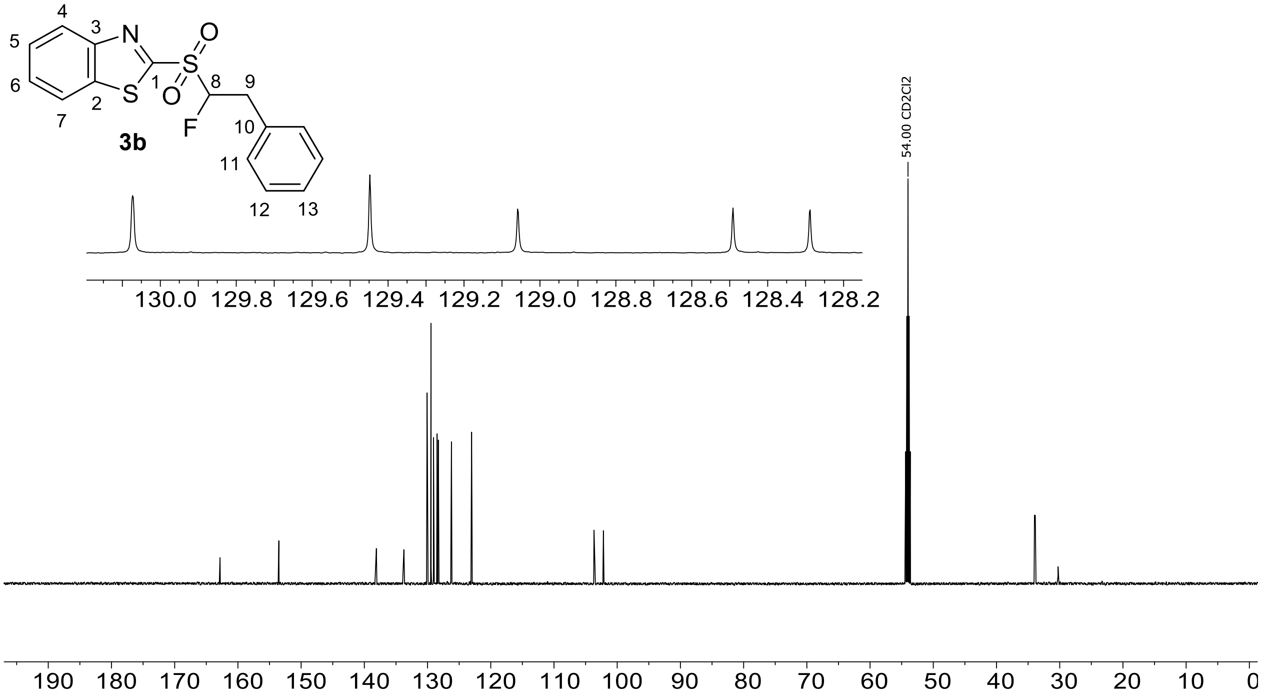

^{19}F NMR (564 MHz, Methylene chloride-d_2, 298 K)

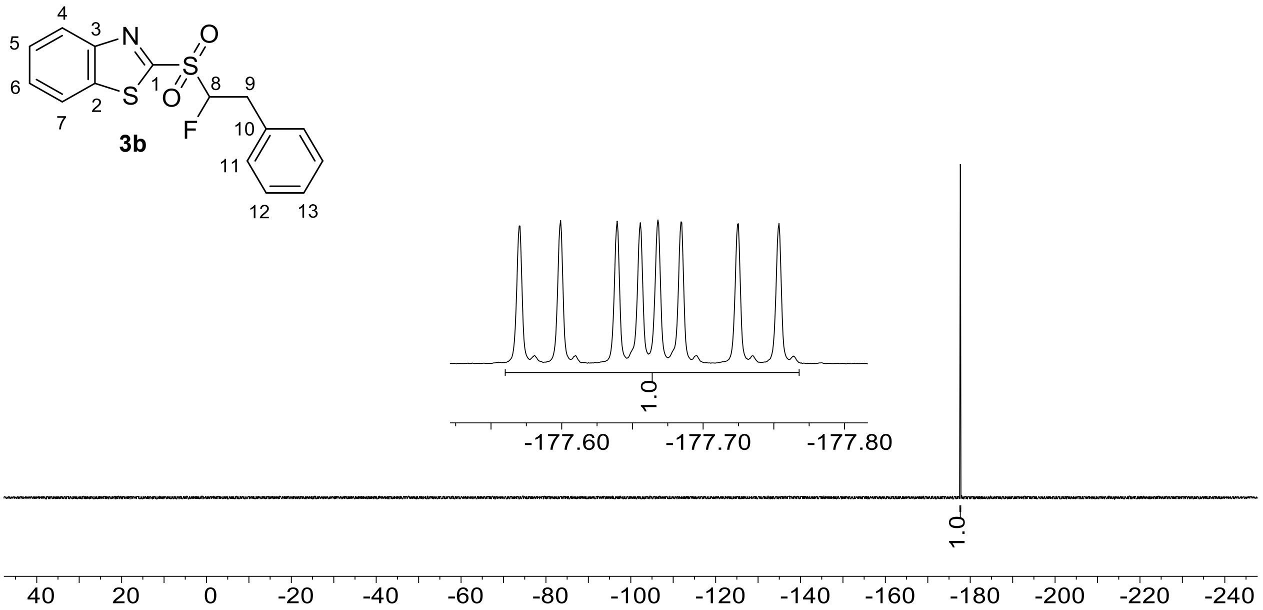

2-((Fluoro(naphthalen-2-yl)methyl)sulfonyl)benzo[d]thiazole (3c)

^{1}H NMR (600 MHz, Methylene chloride-d_2, 298 K)

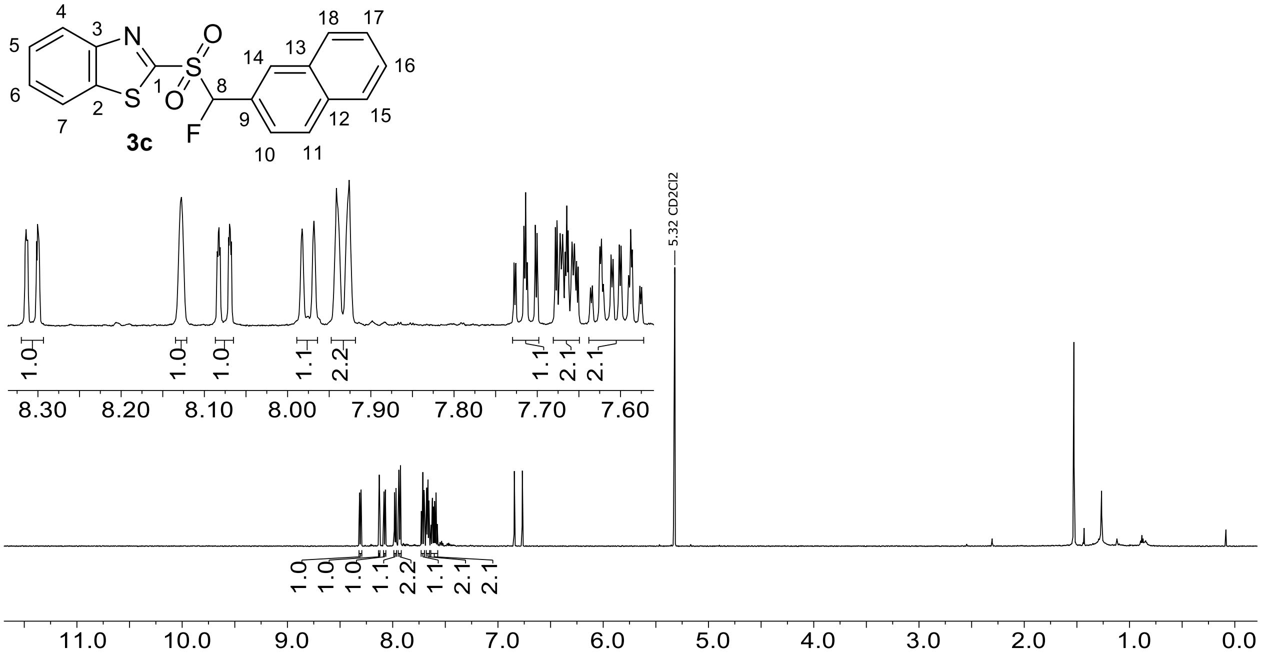

^{13}C{^{1}H} NMR (151 MHz, Methylene chloride-d_2, 298 K)

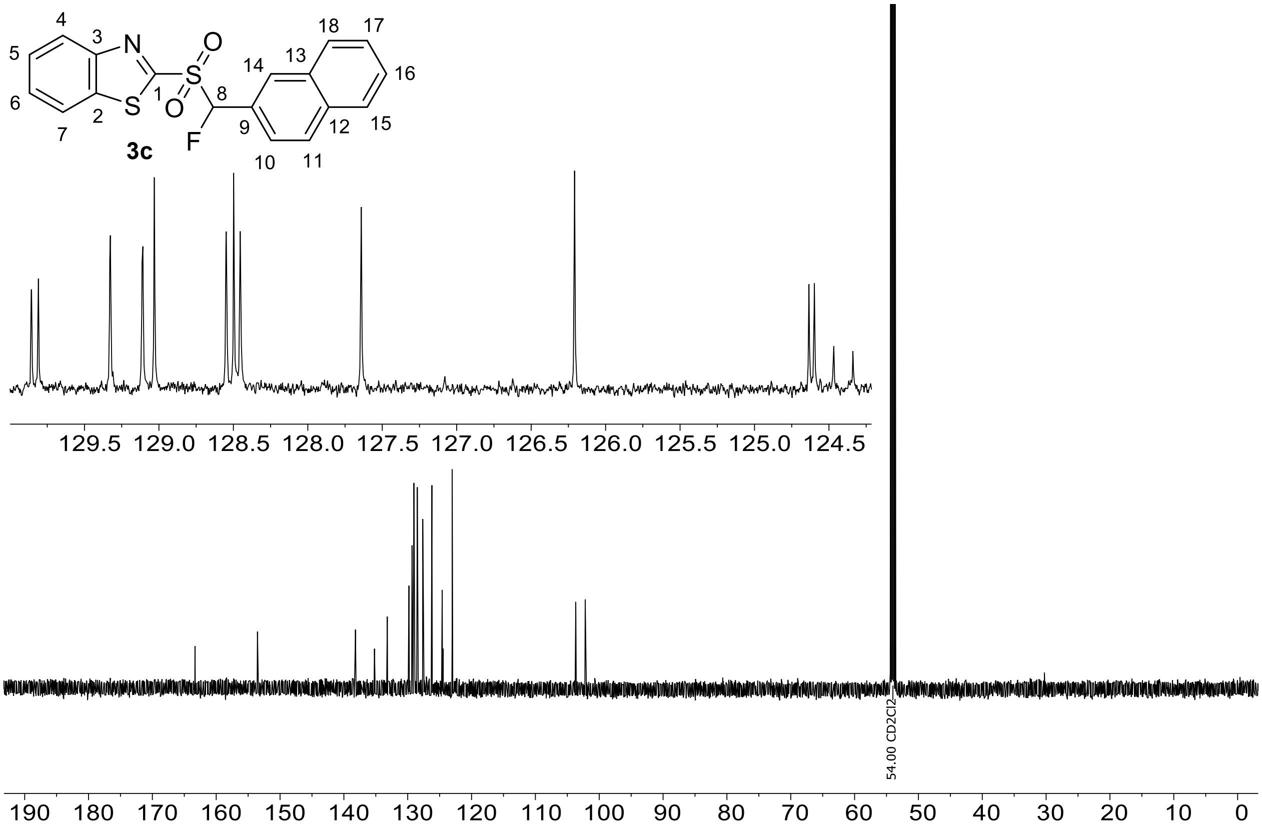

^{19}F NMR (564 MHz, Methylene chloride-d_2, 298 K)

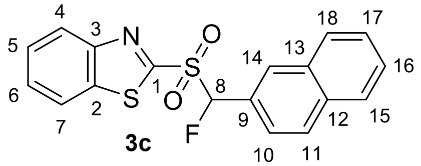

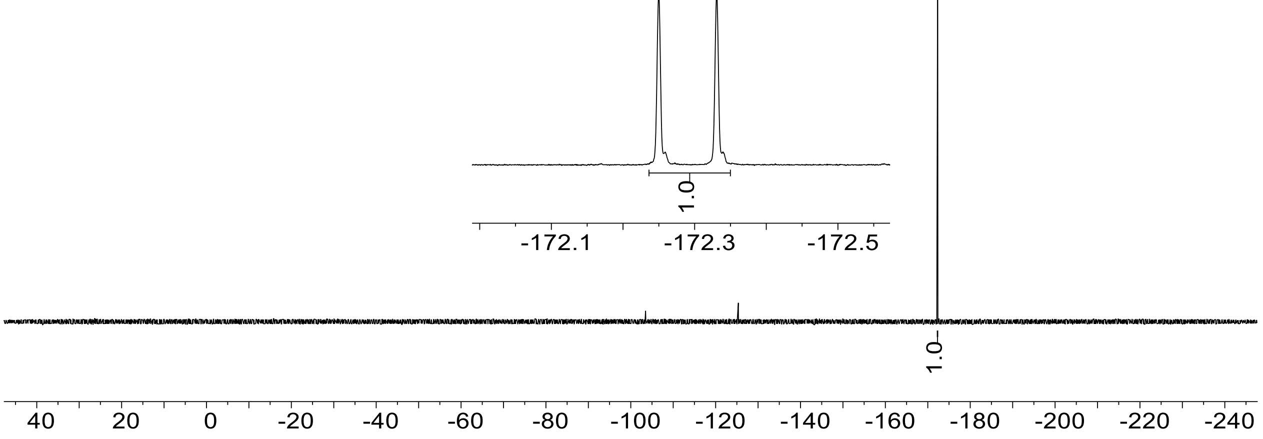

X-Ray Crystallographic Data

X-Ray diffraction: Data sets for compounds **2b**, **3b**, **4b** and **6a** were collected with a D8 Venture Dual Source 100 CMOS diffractometer. For compounds **2c**, **3a**, **3c**, **7a**, **7b** and **8a** data sets were collected with a Bruker APEX II CCD diffractometer. Programs used: data collection: APEX3 V2016.1-0 (Bruker AXS Inc., **2016**); cell refinement: SAINT V8.37A (Bruker AXS Inc., **2015**); data reduction: SAINT V8.37A (Bruker AXS Inc., **2015**); absorption correction, SADABS V2014/7 (Bruker AXS Inc., **2014**); structure solution *SHELXT-2015* (Sheldrick, G. M. *Acta Cryst.,* **2015**, *A71*, 3-8); structure refinement *SHELXL-2015* (Sheldrick, G. M. *Acta Cryst.,* **2015**, *C71* (1), 3-8) and graphics, *XP* (Version 5.1, Bruker AXS Inc., Madison, Wisconsin, USA, **1998**). For compounds **1b**, **1c**, **2a**, **4a**, **5b** and **6b** data sets were collected with a Nonius Kappa CCD diffractometer. Programs used: data collection, COLLECT (R. W. W. Hooft, Bruker AXS, **2008**, Delft, The Netherlands); data reduction Denzo-SMN (Z. Otwinowski, W. Minor, *Methods Enzymol.* **1997**, *276*, 307-326); absorption correction, Denzo (Z. Otwinowski, D. Borek, W. Majewski, W. Minor, *Acta Crystallogr.* **2003**, *A59*, 228-234); structure solution SHELXT-2015; structure refinement SHELXL-2015. *R*-values are given for observed reflections, and wR^2 values are given for all reflections.

Exceptions and special features: Compounds **1c** and **3a** were refined as a 2-component inversion twin. For compound **1c** the 'HKLF 5' option was used, whereby the BASF factor was refined to 0.24. For compound **3a** the options TWIN and BASF (0.38) were used. Additionally, the entire structure of compound **1c** was found disordered over two positions, while for compounds **3a** and **3b** the C(H)-F unit was found disordered over two positions. Several restraints (SADI, SAME, ISOR, FLAT and SIMU) were used in order to improve refinement stability.[1]

X-ray crystal structure analysis of 1b: A colorless plate-like specimen of $C_{15}H_{13}NS_2$, approximate dimensions 0.020 mm x 0.050 mm x 0.150 mm, was used for the X-ray crystallographic analysis. The X-ray intensity data were measured. The integration of the data using a triclinic unit cell yielded a total of 7673 reflections to a maximum θ angle of 25.00° (0.84 Å resolution), of which 4577 were independent (average redundancy 1.676, completeness = 99.0%, R_{int} = 3.96%, R_{sig} = 4.28%) and 4046 (88.40%) were greater than 2σ(F^2). The final cell constants of a = 5.2841(2) Å, b = 10.7718(4) Å, c = 23.4372(9) Å, α = 83.764(3)°, β = 88.159(4)°, γ = 81.5260(10)°, volume = 1311.48(9) $Å^3$, are based upon the refinement of the XYZ-centroids of reflections above 20 σ (I). Data were corrected for absorption effects using the multi-scan method (SADABS). The calculated minimum and maximum transmission coefficients (based on crystal size) are 0.9440 and 0.9920. The structure was solved and refined using the Bruker SHELXTL Software Package, using the space group *P*-1, with Z = 4 for the formula unit, $C_{15}H_{13}NS_2$. The final anisotropic full-matrix least-squares refinement on F^2 with 325 variables converged at R1 = 4.40%, for the observed data and wR2 = 11.93% for all data. The goodness-of-fit was 1.038. The largest peak in the final difference electron density synthesis was 0.295 $e^-/Å^3$ and the largest hole was -0.357 $e^-/Å^3$ with an RMS deviation of 0.055 $e^-/Å^3$. On the basis of the final model, the calculated density was 1.374 g/cm^3 and F(000), 568 e^-. CCDC number: 1969648.

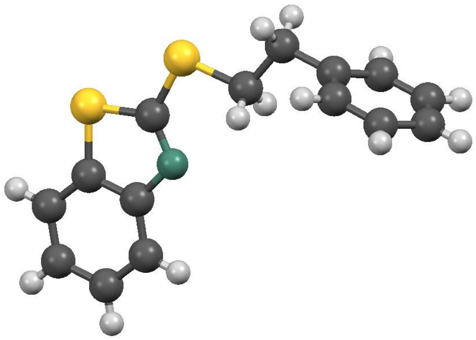

X-ray crystal structure analysis of 1c: A colorless plate-like specimen of $C_{18}H_{13}NS_2$, approximate dimensions 0.050 mm x 0.100 mm x 0.230 mm, was used for the X-ray crystallographic analysis. The X-ray intensity data were measured. The integration of the data using a triclinic unit cell yielded a total of 2482 reflections to a maximum θ angle of 25.00° (0.84 Å resolution), of which 2482 were independent (average redundancy 1.000, completeness = 97.3%, R_{sig} = 4.25%) and 2198 (88.56%) were greater than 2σ(F^2). The final cell constants of a = 5.9108(2) Å, b = 7.6651(3) Å, c = 16.5659(7) Å, α = 78.414(3)°, β = 83.039(3)°, γ = 89.177(2)°, volume = 729.80(5) $Å^3$, are based upon the refinement of the XYZ-centroids of reflections above 20 σ (I). Data were corrected for absorption effects using the multi-scan method (SADABS). The calculated minimum and maximum transmission coefficients (based on crystal size) are 0.9230 and 0.9820. The structure was solved and refined using the Bruker SHELXTL Software Package, using the space group *P*-1, with Z = 2 for the formula unit, $C_{18}H_{13}NS_2$. The final anisotropic full-matrix least-squares refinement on F^2 with 376 variables converged at R1 = 6.57%, for the observed data and wR2 = 17.53% for all data. The goodness-of-fit was 1.169. The largest peak in the final difference electron density synthesis was 0.193 $e^-/Å^3$ and the largest hole was -0.228 $e^-/Å^3$ with an RMS deviation of 0.057 $e^-/Å^3$. On the basis of the final model, the calculated density was 1.399 g/cm^3 and F(000), 320 e^-. CCDC number: 1969649.

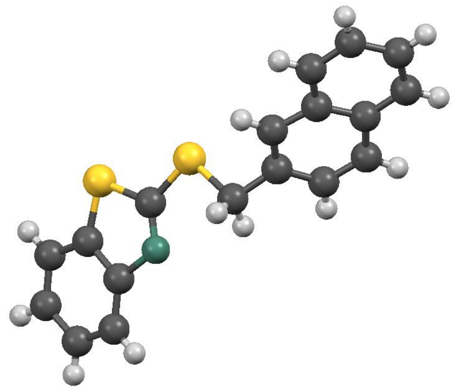

X-ray crystal structure analysis of 2a: A colorless plate-like specimen of $C_{14}H_{11}NO_2S_2$, approximate dimensions 0.040 mm x 0.080 mm x 0.140 mm, was used for the X-ray crystallographic analysis. The X-ray intensity data were measured. The integration of the data using a monoclinic unit cell yielded a total of 2301 reflections to a maximum θ angle of 25.00° (0.84 Å resolution), of which 2301 were independent (average redundancy 1.000, completeness = 98.2%, R_{sig} = 2.96%) and 2044 (88.83%) were greater than $2\sigma(F^2)$. The final cell constants of a = 13.9979(7) Å, b = 11.9471(6) Å, c = 8.1283(4) Å, β = 102.383(2)°, volume = 1327.71(11) $Å^3$, are based upon the refinement of the XYZ-centroids of reflections above $20\sigma(I)$. Data were corrected for absorption effects using the multi-scan method (SADABS). The calculated minimum and maximum transmission coefficients (based on crystal size) are 0.9470 and 0.9840. The structure was solved and refined using the Bruker SHELXTL Software Package, using the space group $P2_1/c$, with Z = 4 for the formula unit, $C_{14}H_{11}NO_2S_2$. The final anisotropic full-matrix least-squares refinement on F^2 with 172 variables converged at R1 = 4.45%, for the observed data and wR2 = 10.56% for all data. The goodness-of-fit was 1.075. The largest peak in the final difference electron density synthesis was 0.203 $e^-/Å^3$ and the largest hole was -0.341 $e^-/Å^3$ with an RMS deviation of 0.048 $e^-/Å^3$. On the basis of the final model, the calculated density was 1.448 g/cm^3 and F(000), 600 e^-. CCDC number: 1969650.

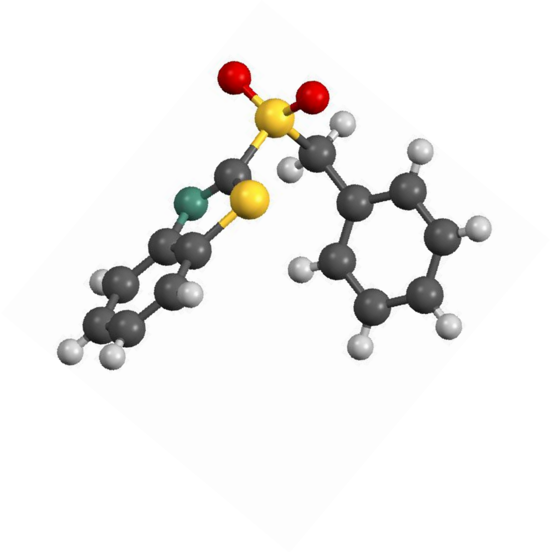

X-ray crystal structure analysis of 2b: A colorless prism-like specimen of $C_{15}H_{13}NO_2S_2$, approximate dimensions 0.132 mm x 0.179 mm x 0.328 mm, was used for the X-ray crystallographic analysis. The X-ray intensity data were measured. A total of 1718 frames were collected. The total exposure time was 16.94 hours. The frames were integrated with the Bruker SAINT software package using a wide-frame algorithm. The integration of the data using a monoclinic unit cell yielded a total of 39232 reflections to a maximum θ angle of 70.17° (0.82 Å resolution), of which 5274 were independent (average redundancy 7.439, completeness = 99.5%, R_{int} = 3.61%, R_{sig} = 2.28%) and 4916 (93.21%) were greater than $2\sigma(F^2)$. The final cell constants of a = 11.2140(3) Å, b = 8.2348(2) Å, c = 30.7187(8) Å, β = 100.4930(10)°, volume = 2789.28(12) Å^3, are based upon the refinement of the XYZ-centroids of 9942 reflections above $20\sigma(I)$ with 5.852° < 2 θ < 140.1°. Data were corrected for absorption effects using the multi-scan method (SADABS). The ratio of minimum to maximum apparent transmission was 0.851. The calculated minimum and maximum transmission coefficients (based on crystal size) are 0.3960 and 0.6580. The structure was solved and refined using the Bruker SHELXTL Software Package, using the space group $P2_1/c$, with Z = 8 for the formula unit, $C_{15}H_{13}NO_2S_2$. The final anisotropic full-matrix least-squares refinement on F^2 with 361 variables converged at R1 = 3.36%, for the observed data and wR2 = 8.44% for all data. The goodness-of-fit was 1.047. The largest peak in the final difference electron density synthesis was 0.586 $e^-/Å^3$ and the largest hole was -0.517 $e^-/Å^3$ with an RMS deviation of 0.054 $e^-/Å^3$. On the basis of the final model, the calculated density was 1.445 g/cm^3 and F(000), 1264 e^-. CCDC number: 1969651.

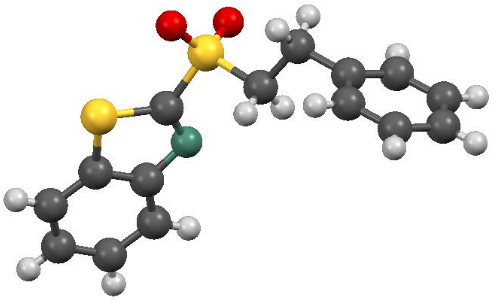

X-ray crystal structure analysis of 2c: A colorless needle-like specimen of $C_{18}H_{13}NO_2S_2$, approximate dimensions 0.030 mm x 0.080 mm x 0.140 mm, was used for the X-ray crystallographic analysis. The X-ray intensity data were measured. A total of 1610 frames were collected. The total exposure time was 20.01 hours. The frames were integrated with the Bruker SAINT software package using a wide-frame algorithm. The integration of the data using a monoclinic unit cell yielded a total of 22016 reflections to a maximum θ angle of 66.61° (0.84 Å resolution), of which 2642 were independent (average redundancy 8.333, completeness = 99.2%, R_{int} = 7.30%, R_{sig} = 3.62%) and 2190 (82.89%) were greater than 2σ(F^2). The final cell constants of a = 5.8006(2) Å, b = 17.2202(6) Å, c = 15.1139(5) Å, β = 90.488(2)°, volume = 1509.63(9) $Å^3$, are based upon the refinement of the XYZ-centroids of 6521 reflections above 20σ(I) with 7.783° < 2θ < 132.9°. Data were corrected for absorption effects using the multi-scan method (SADABS). The ratio of minimum to maximum apparent transmission was 0.802. The calculated minimum and maximum transmission coefficients (based on crystal size) are 0.6570 and 0.9080. The structure was solved and refined using the Bruker SHELXTL Software Package, using the space group $P2_1/c$, with Z = 4 for the formula unit, $C_{18}H_{13}NO_2S_2$. The final anisotropic full-matrix least-squares refinement on F^2 with 208 variables converged at R1 = 4.75%, for the observed data and wR2 = 12.77% for all data. The goodness-of-fit was 1.048. The largest peak in the final difference electron density synthesis was 1.251 $e^-/Å^3$ and the largest hole was -0.445 $e^-/Å^3$ with an RMS deviation of 0.077 $e^-/Å^3$. On the basis of the final model, the calculated density was 1.493 g/cm^3 and F(000), 704 e^-. CCDC number: 1969652.

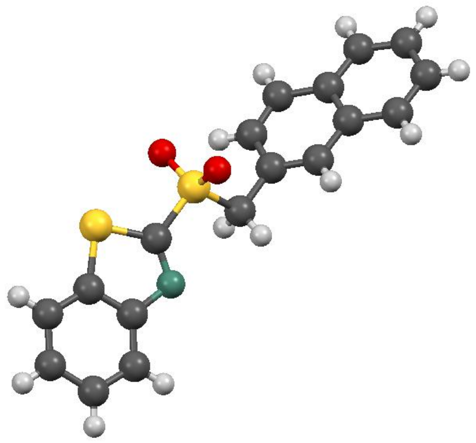

X-ray crystal structure analysis of 3a: A colorless plate-like specimen of $C_{14}H_{10}FNO_2S_2$, approximate dimensions 0.020 mm x 0.100 mm x 0.140 mm, was used for the X-ray crystallographic analysis. The X-ray intensity data were measured. A total of 1071 frames were collected. The total exposure time was 19.30 hours. The frames were integrated with the Bruker SAINT software package using a wide-frame algorithm. The integration of the data using an orthorhombic unit cell yielded a total of 13422 reflections to a maximum θ angle of 66.72° (0.84 Å resolution), of which 2348 were independent (average redundancy 5.716, completeness = 100.0%, R_{int} = 4.74%, R_{sig} = 3.18%) and 2230 (94.97%) were greater than 2σ(F^2). The final cell constants of a = 5.6585(2) Å, b = 13.5586(4) Å, c = 17.3178(5) Å, volume = 1328.64(7) $Å^3$, are based upon the refinement of the

XYZ-centroids of 7250 reflections above 20σ(I) with 8.282° < 2θ < 133.5°. Data were corrected for absorption effects using the multi-scan method (SADABS). The ratio of minimum to maximum apparent transmission was 0.851. The calculated minimum and maximum transmission coefficients (based on crystal size) are 0.6210 and 0.9290. The structure was solved and refined using the Bruker SHELXTL Software Package, using the space group $P2_12_12_1$, with Z = 4 for the formula unit, $C_{14}H_{10}FNO_2S_2$. The final anisotropic full-matrix least-squares refinement on F^2 with 187 variables converged at R1 = 3.03%, for the observed data and wR2 = 7.12% for all data. The goodness-of-fit was 1.115. The largest peak in the final difference electron density synthesis was 0.259 $e^-/Å^3$ and the largest hole was -0.207 $e^-/Å^3$ with an RMS deviation of 0.050 $e^-/Å^3$. On the basis of the final model, the calculated density was 1.537 g/cm^3 and F(000), 632 e^-. CCDC number: 1969653.

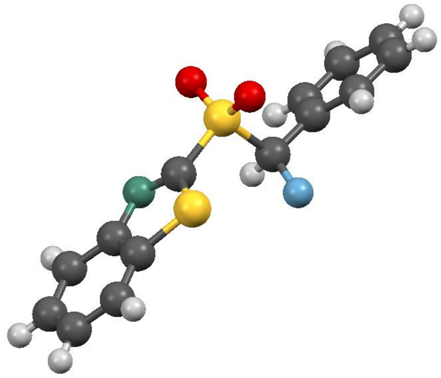

X-ray crystal structure analysis of 3b: A colorless plate-like specimen of $C_{15}H_{12}FNO_2S_2$, approximate dimensions 0.046 mm x 0.160 mm x 0.617 mm, was used for the X-ray crystallographic analysis. The X-ray intensity data were measured. A total of 1530 frames were collected. The total exposure time was 22.81 hours. The frames were integrated with the Bruker SAINT software package using a wide-frame algorithm. The integration of the data using a monoclinic unit cell yielded a total of 24052 reflections to a maximum θ angle of 70.23° (0.82 Å resolution), of which 2865 were independent (average redundancy 8.395, completeness = 99.7%, R_{int} = 20.05%, R_{sig} = 7.09%) and 2729 (95.25%) were greater than 2σ(F^2). The final cell constants of a = 15.3919(5) Å, b = 6.9375(2) Å, c = 15.7094(5) Å, β = 116.2910(10)°, volume = 1503.95(8) Å^3, are based upon the refinement of the XYZ-centroids of 9883 reflections above 20σ(I) with 10.78° < 2θ < 140.4°. Data were corrected for absorption effects using the multi-scan method (SADABS). The ratio of minimum to maximum apparent transmission was 0.608. The calculated minimum and maximum transmission coefficients (based on crystal size) are 0.2320 and 0.8610. The structure was solved and refined using the Bruker SHELXTL Software Package, using the space group $P2_1/n$, with Z = 4 for the formula unit, $C_{15}H_{12}FNO_2S_2$. The final anisotropic full-matrix least-squares refinement on F^2 with 200 variables converged at R1 = 5.38%, for the observed data and wR2 = 13.33% for all data. The goodness-of-fit was 1.084. The largest peak in the final difference electron density synthesis was 0.356 $e^-/Å^3$ and the largest hole was -0.571 $e^-/Å^3$ with an RMS deviation of 0.092 $e^-/Å^3$. On the basis of the final model, the calculated density was 1.419 g/cm^3 and F(000), 664 e^-. CCDC number: 1969654.

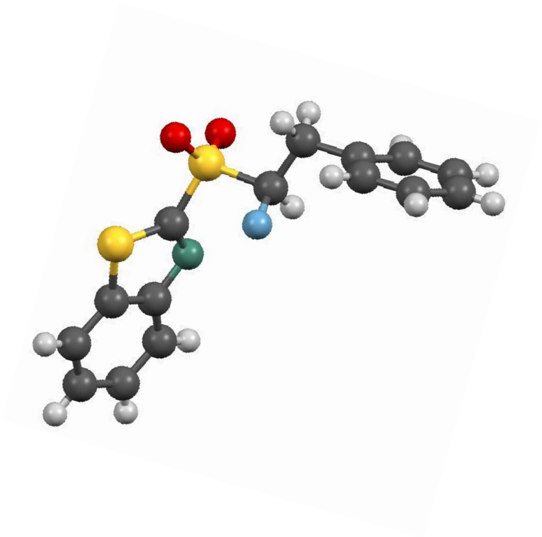

X-ray crystal structure analysis of 3c: A colorless needle-like specimen of $C_{18}H_{12}FNO_2S_2$, approximate dimensions 0.050 mm x 0.050 mm x 0.180 mm, was used for the X-ray crystallographic analysis. The X-ray intensity data were measured. A total of 1391 frames were collected. The total exposure time was 22.30 hours. The frames were integrated with the Bruker SAINT software package using a wide-frame algorithm. The integration of the data using an orthorhombic unit cell yielded a total of 17886 reflections to a maximum θ angle of 66.61° (0.84 Å resolution), of which 2730 were independent (average redundancy 6.552, completeness = 99.9%, R_{int} = 8.23%, R_{sig} = 5.35%) and 2403 (88.02%) were greater than $2\sigma(F^2)$. The final cell constants of a = 5.8636(2) Å, b = 12.1210(3) Å, c = 21.7943(6) Å, volume = 1548.98(8) $Å^3$, are based upon the refinement of the XYZ-centroids of 5053 reflections above $20\sigma(I)$ with $8.113° < 2\theta < 132.5°$. Data were corrected for absorption effects using the multi-scan method (SADABS). The ratio of minimum to maximum apparent transmission was 0.740. The calculated minimum and maximum transmission coefficients (based on crystal size) are 0.5870 and 0.8520. The structure was solved and refined using the Bruker SHELXTL Software Package, using the space group $P2_12_12_1$, with Z = 4 for the formula unit, $C_{18}H_{12}FNO_2S_2$. The final anisotropic full-matrix least-squares refinement on F^2 with 217 variables converged at R1 = 5.07%, for the observed data and wR2 = 12.17% for all data. The goodness-of-fit was 1.049. The largest peak in the final difference electron density synthesis was 0.541 $e^-/Å^3$ and the largest hole was -0.260 $e^-/Å^3$ with an RMS deviation of 0.065 $e^-/Å^3$. On the basis of the final model, the calculated density was 1.533 g/cm^3 and F(000), 736 e^-. Flack parameter was refined to 0.04(2). CCDC number: 1969655.

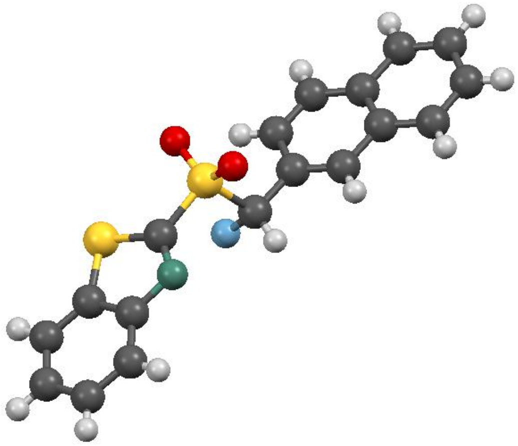

X-ray crystal structure analysis of 4a: A colorless needle-like specimen of $C_{14}H_{11}NOS_2$, approximate dimensions 0.080 mm x 0.100 mm x 0.550 mm, was used for the X-ray crystallographic analysis. The X-ray intensity data were measured. The integration of the data using an orthorhombic unit cell yielded a total of 2191 reflections to a maximum θ angle of 24.98° (0.84 Å resolution), of which 2191 were independent (average redundancy 1.000, completeness = 98.7%, R_{sig} = 1.83%) and 2142 (97.76%) were greater than 2σ(F^2). The final cell constants of a = 5.73670(10) Å, b = 13.2513(3) Å, c = 16.5649(6) Å, volume = 1259.24(6) Å^3, are based upon the refinement of the XYZ-centroids of reflections above 20σ(I). Data were corrected for absorption effects using the multi-scan method (SADABS). The calculated minimum and maximum transmission coefficients (based on crystal size) are 0.8070 and 0.9680. The structure was solved and refined using the Bruker SHELXTL Software Package, using the space group $P2_12_12_1$, with Z = 4 for the formula unit, $C_{14}H_{11}NOS_2$. The final anisotropic full-matrix least-squares refinement on F^2 with 163 variables converged at R1 = 2.54%, for the observed data and wR2 = 6.61% for all data. The goodness-of-fit was 1.077. The largest peak in the final difference electron density synthesis was 0.157 e$^-$/Å^3 and the largest hole was -0.163 e$^-$/Å^3 with an RMS deviation of 0.034 e$^-$/Å^3. On the basis of the final model, the calculated density was 1.442 g/cm^3 and F(000), 568 e$^-$. Flack parameter was refined to 0.02(5). CCDC number: 1969656.

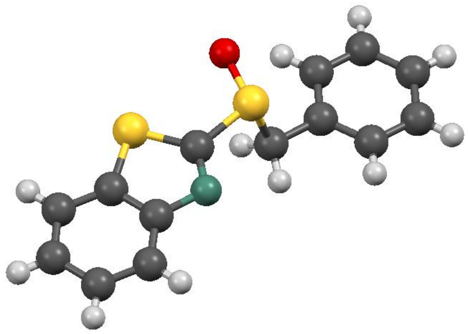

X-ray crystal structure analysis of 4b: A colorless prism-like specimen of $C_{15}H_{13}NOS_2$, approximate dimensions 0.175 mm x 0.181 mm x 0.237 mm, was used for the X-ray crystallographic analysis. The X-ray intensity data were measured. A total of 1890 frames were collected. The total exposure time was 18.23 hours. The frames were integrated with the Bruker SAINT software package using a wide-frame algorithm. The integration of the data using a monoclinic unit cell yielded a total of 8251 reflections to a maximum θ angle of 69.80° (0.82 Å resolution), of which 2397 were independent (average redundancy 3.442, completeness = 99.0%, R_{int} = 2.51%, R_{sig} = 3.24%) and 2385 (99.50%) were greater than 2σ(F^2). The final cell constants of a = 14.4369(5) Å, b = 10.4206(4) Å, c = 10.6549(6) Å, β = 122.5020(10)°, volume = 1351.87(10) $Å^3$, are based upon the refinement of the XYZ-centroids of 8523 reflections above 20 σ(I) with 11.17° < 2θ < 139.6°. Data were corrected for absorption effects using the multi-scan method (SADABS). The ratio of minimum to maximum apparent transmission was 0.866. The calculated minimum and maximum transmission coefficients (based on crystal size) are 0.4920 and 0.5810. The final anisotropic full-matrix least-squares refinement on F^2 with 172 variables converged at R1 = 2.21%, for the observed data and wR2 = 5.71% for all data. The goodness-of-fit was 1.105. The largest peak in the final difference electron density synthesis was 0.157 $e^-/Å^3$ and the largest hole was -0.248 $e^-/Å^3$ with an RMS deviation of 0.038 $e^-/Å^3$. On the basis of the final model, the calculated density was 1.412 g/cm^3 and F(000), 600 e^-. CCDC number: 1969657.

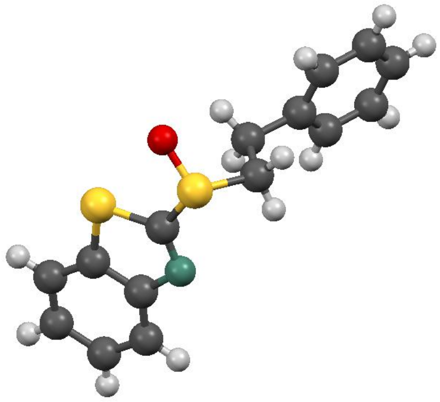

X-ray crystal structure analysis of 5b: A colorless needle-like specimen of $C_{15}H_{12}FNS_2$, approximate dimensions 0.020 mm x 0.040 mm x 0.120 mm, was used for the X-ray crystallographic analysis. The X-ray intensity data were measured. The integration of the data using a monoclinic unit cell yielded a total of 2284 reflections to a maximum θ angle of 25.00° (0.84 Å resolution), of which 2284 were independent (average redundancy 1.000, completeness = 96.8%, R_{sig} = 2.60%) and 2063 (90.32%) were greater than 2σ(F^2). The final cell constants of a = 9.3000(2) Å, b = 10.1527(2) Å, c = 14.3279(4) Å, β = 96.7830(10)°, volume = 1343.37(5) $Å^3$, are based upon the refinement of the XYZ-centroids of reflections above 20 σ(I). Data were corrected for absorption effects using the multi-scan method (SADABS). The calculated minimum and maximum transmission coefficients (based on crystal size) are 0.9550 and 0.9920. The structure was solved and refined using the Bruker SHELXTL Software Package, using the space group $P2_1/c$, with Z = 4 for the formula unit, $C_{15}H_{12}FNS_2$. The final anisotropic full-matrix least-squares refinement on F^2 with 172 variables converged at R1 = 5.40%, for the observed data and wR2 = 14.94% for all data. The goodness-of-fit was 1.062. The largest peak in the final difference electron density synthesis was 0.364 $e^-/Å^3$ and the

largest hole was -0.288 e$^-$/Å^3 with an RMS deviation of 0.077 e$^-$/Å^3. On the basis of the final model, the calculated density was 1.431 g/cm^3 and F(000), 600 e$^-$. CCDC number: 1969658.

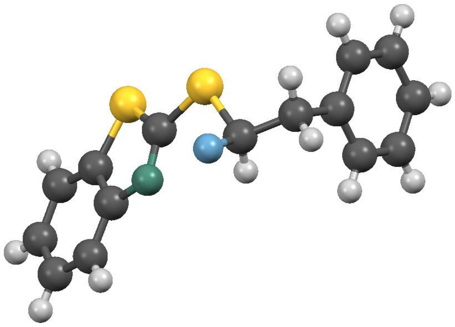

X-ray crystal structure analysis of 6a: A colorless needle-like specimen of $C_{14}H_{10}FNOS_2$, approximate dimensions 0.035 mm x 0.083 mm x 0.250 mm, was used for the X-ray crystallographic analysis. The X-ray intensity data were measured. A total of 1663 frames were collected. The total exposure time was 29.56 hours. The frames were integrated with the Bruker SAINT software package using a wide-frame algorithm. The integration of the data using a monoclinic unit cell yielded a total of 17943 reflections to a maximum θ angle of 66.76° (0.84 Å resolution), of which 2290 were independent (average redundancy 7.835, completeness = 99.3%, R_{int} = 8.93%, R_{sig} = 4.96%) and 1929 (84.24%) were greater than $2\sigma(F^2)$. The final cell constants of a = 19.8934(6) Å, b = 5.5617(2) Å, c = 23.5265(8) Å, β = 96.937(2)°, volume = 2583.94(15) Å^3, are based upon the refinement of the XYZ-centroids of 8802 reflections above 20 σ(I) with 7.570° < 2θ < 133.3°. Data were corrected for absorption effects using the multi-scan method (SADABS). The ratio of minimum to maximum apparent transmission was 0.785. The calculated minimum and maximum transmission coefficients (based on crystal size) are 0.4520 and 0.8790. The structure was solved and refined using the Bruker SHELXTL Software Package, using the space group *C*2/*c*, with Z = 8 for the formula unit, $C_{14}H_{10}FNOS_2$. The final anisotropic full-matrix least-squares refinement on F^2 with 172 variables converged at R1 = 4.98%, for the observed data and wR2 = 9.76% for all data. The goodness-of-fit was 1.118. The largest peak in the final difference electron density synthesis was 0.257 e$^-$/Å^3 and the largest hole was -0.359 e$^-$/Å^3 with an RMS deviation of 0.069 e$^-$/Å^3. On the basis of the final model, the calculated density was 1.498 g/cm^3 and F(000), 1200 e$^-$. CCDC number: 1969659.

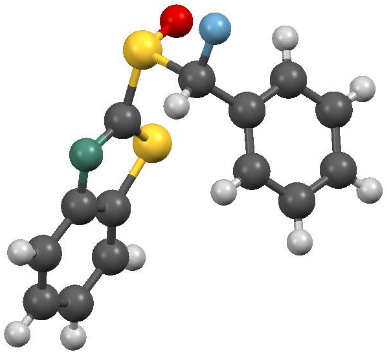

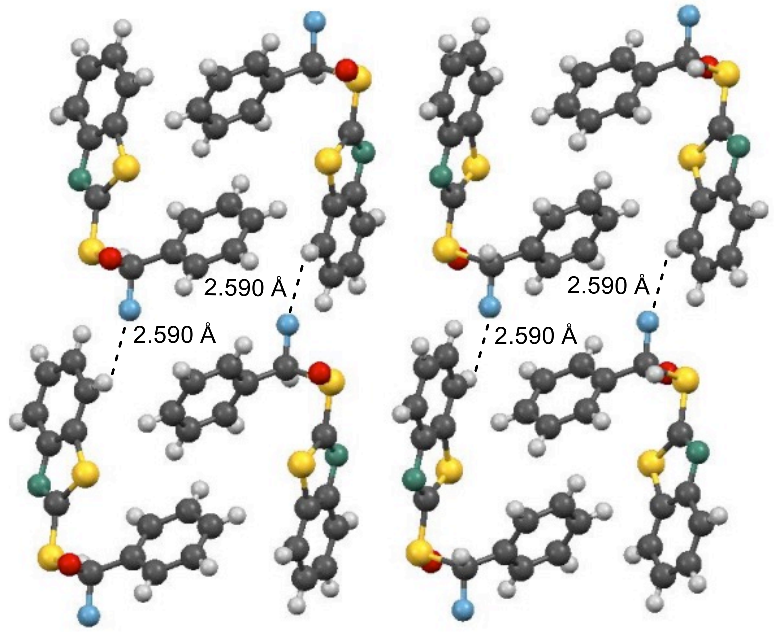

Figure S1. F-H hydrogen bonding interactions in the crystal of **6a**.

X-ray crystal structure analysis of 6b: A colorless needle-like specimen of $C_{14}H_{10}FNOS_2$, approximate dimensions 0.020 mm x 0.030 mm x 0.080 mm, was used for the X-ray crystallographic analysis. The X-ray intensity data were measured. The integration of the data using an orthorhombic unit cell yielded a total of 2131 reflections to a maximum θ angle of 25.00° (0.84 Å resolution), of which 2131 were independent (average redundancy 1.000, completeness = 97.7%, R_{sig} = 3.07%) and 2063 (96.81%) were greater than 2σ(F^2). The final cell constants of a = 5.6348(2) Å, b = 13.4906(4) Å, c = 16.7334(5) Å, volume = 1272.02(7) $Å^3$, are based upon the refinement of the XYZ-centroids of reflections above 20 σ(I). The calculated minimum and maximum transmission coefficients (based on crystal size) are 0.9670 and 0.9920. The structure was solved and refined using the Bruker SHELXTL Software Package, using the space group $P2_12_12_1$, with Z = 4 for the formula unit, $C_{14}H_{10}FNOS_2$. The final anisotropic full-matrix least-squares refinement on F^2 with 172 variables converged at R1 = 5.21%, for the observed data and wR2 = 13.94% for all data. The goodness-of-fit was 1.059. The largest peak in the final difference electron density synthesis was 0.281 $e^-/Å^3$ and the largest hole was -0.255 $e^-/Å^3$ with an RMS deviation of 0.067 $e^-/Å^3$. On the basis of the final model, the calculated density was 1.521 g/cm^3 and F(000), 600 e^-. Flack parameter was refined to 0.17(15). CCDC number: 1969660.

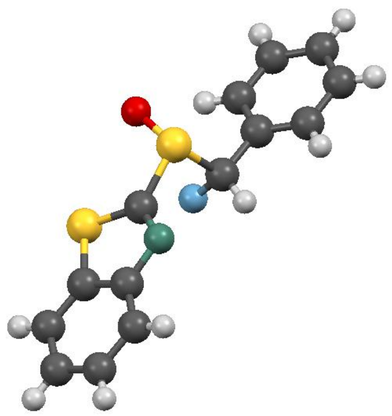

X-ray crystal structure analysis of 7a: A colorless needle-like specimen of $C_{15}H_{12}FNOS_2$, approximate dimensions 0.030 mm x 0.050 mm x 0.140 mm, was used for the X-ray crystallographic analysis. The X-ray intensity data were measured. A total of 1023 frames were collected. The total exposure time was 19.34 hours. The frames were integrated with the Bruker SAINT software package using a wide-frame algorithm. The integration of the data using an orthorhombic unit cell yielded a total of 11004 reflections to a maximum θ angle of 66.72° (0.84 Å resolution), of which 2327 were independent (average redundancy 4.729, completeness = 99.2%, R_{int} = 4.43%, R_{sig} = 3.74%) and 2243 (96.39%) were greater than 2σ(F^2). The final cell constants of a = 21.0286(9) Å, b = 11.0349(5) Å, c = 5.8074(3) Å, volume = 1347.60(11) $Å^3$, are based upon the refinement of the XYZ-centroids of 5219 reflections above 20 σ(I) with 8.409° < 2θ < 133.2°. Data were corrected for absorption effects using the multi-scan method (SADABS). The ratio of minimum to maximum apparent transmission was 0.795. The calculated minimum and maximum transmission coefficients (based on crystal size) are 0.6300 and 0.8990. The structure was solved and refined using the Bruker SHELXTL Software Package, using the space group $Pna2_1$, with Z = 4 for the formula unit, $C_{15}H_{12}FNOS_2$. The final anisotropic full-matrix least-squares refinement on F^2 with 181 variables converged at R1 = 2.49%, for the observed data and wR2 = 5.83% for all data. The goodness-of-fit was 1.082. The largest peak in the final difference electron density synthesis was 0.210 $e^-/Å^3$ and the largest hole was -0.193 $e^-/Å^3$ with an RMS deviation of 0.043 $e^-/Å^3$. On the basis of the final model, the calculated density was 1.505 g/cm^3 and F(000), 632 e^-. CCDC number: 1969661.

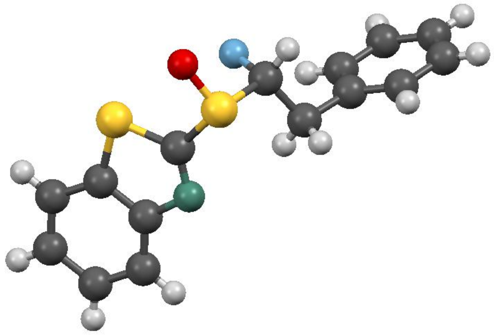

X-ray crystal structure analysis of 7b: A colorless needle-like specimen of $C_{15}H_{12}FNOS_2$, approximate dimensions 0.020 mm x 0.060 mm x 0.280 mm, was used for the X-ray crystallographic

analysis. The X-ray intensity data were measured. A total of 1318 frames were collected. The total exposure time was 20.41 hours. The frames were integrated with the Bruker SAINT software package using a wide-frame algorithm. The integration of the data using a triclinic unit cell yielded a total of 9428 reflections to a maximum θ angle of 66.84° (0.84 Å resolution), of which 2412 were independent (average redundancy 3.909, completeness = 98.8%, R_{int} = 3.70%, R_{sig} = 3.22%) and 2234 (92.62%) were greater than 2σ(F^2). The final cell constants of a = 5.9200(2) Å, b = 8.2828(3) Å, c = 14.8444(5) Å, α = 79.548(2)°, β = 85.207(2)°, γ = 74.436(2)°, volume = 689.09(4) $Å^3$, are based upon the refinement of the XYZ-centroids of 5646 reflections above 20 σ(I) with 6.058° < 2θ < 133.6°. Data were corrected for absorption effects using the multi-scan method (SADABS). The ratio of minimum to maximum apparent transmission was 0.739. The calculated minimum and maximum transmission coefficients (based on crystal size) are 0.4350 and 0.9320. The structure was solved and refined using the Bruker SHELXTL Software Package, using the space group *P*-1, with Z = 2 for the formula unit, $C_{15}H_{12}FNOS_2$. The final anisotropic full-matrix least-squares refinement on F^2 with 181 variables converged at R1 = 3.03%, for the observed data and wR2 = 8.08% for all data. The goodness-of-fit was 1.064. The largest peak in the final difference electron density synthesis was 0.294 $e^-/Å^3$ and the largest hole was -0.305 $e^-/Å^3$ with an RMS deviation of 0.057 $e^-/Å^3$. On the basis of the final model, the calculated density was 1.472 g/cm^3 and F(000), 316 e^-. CCDC number: 1969662.

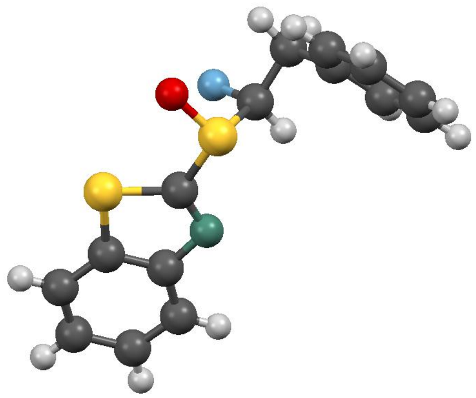

X-ray crystal structure analysis of 8b: A colorless needle-like specimen of $C_{18}H_{12}FNOS_2$, approximate dimensions 0.060 mm x 0.060 mm x 0.220 mm, was used for the X-ray crystallographic analysis. The X-ray intensity data were measured. A total of 1502 frames were collected. The total exposure time was 21.47 hours. The frames were integrated with the Bruker SAINT software package using a wide-frame algorithm. The integration of the data using a monoclinic unit cell yielded a total of 23494 reflections to a maximum θ angle of 66.63° (0.84 Å resolution), of which 2626 were independent (average redundancy 8.947, completeness = 99.9%, R_{int} = 5.24%, R_{sig} = 2.60%) and 2402 (91.47%) were greater than 2σ(F^2). The final cell constants of a = 6.3878(3) Å, b = 16.3971(7) Å, c = 14.1976(6) Å, β = 92.907(2)°, volume = 1485.16(11) $Å^3$, are based upon the refinement of the XYZ-centroids of 7460 reflections above 20 σ(I) with 10.79° < 2θ < 133.1°. Data were corrected for absorption effects using the multi-scan method (SADABS). The ratio of minimum to maximum apparent transmission was 0.792. The calculated minimum and maximum transmission coefficients (based on crystal size) are 0.5240 and 0.8230. The structure was solved and refined using the Bruker SHELXTL Software Package, using the space group $P2_1/c$, with Z = 4 for the formula unit, $C_{18}H_{12}FNOS_2$. The final anisotropic full-matrix least-squares refinement on F^2 with

208 variables converged at R1 = 3.87%, for the observed data and wR2 = 10.17% for all data. The goodness-of-fit was 1.096. The largest peak in the final difference electron density synthesis was 0.751 $e^-/Å^3$ and the largest hole was -0.289 $e^-/Å^3$ with an RMS deviation of 0.062 $e^-/Å^3$. On the basis of the final model, the calculated density was 1.527 g/cm^3 and F(000), 704 e^-. CCDC number: 1969663.

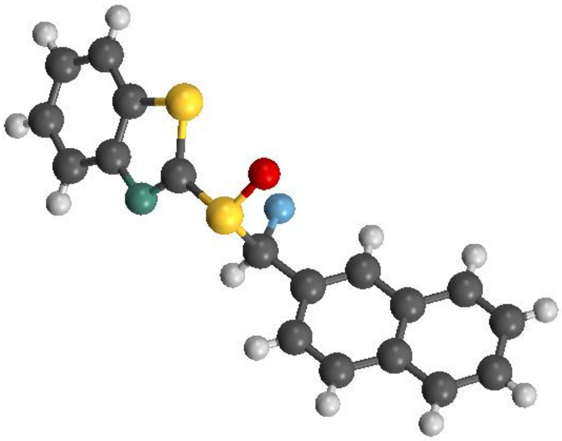

Computational Study of Conformers in Solution

We have performed a rigorous computational search for the prevailing conformations of sulfoxides **6a/b**, **7a/b**, **8a**, sulfones **3a-c**, and sulfide **5b**. Utilizing a procedure originally developed for the generation of conformer-rotamer ensembles for the prediciton of spin-spin-coupled NMR spectra[2], the conformer structures were generated with the semiempirical GFN2-xTB method.[3] Further optimization of relevant conformations were run with the PBEh-3c small basis set functional[4] and addition of rigid-rotor-harmonic-oscillator free energy contributions (G^{RRHO}) as calculated with GFN2-xTB. Electronic single point energies were obtained with the PW6B95-D3,[5] dispersion corrected[6] hybrid functional using a flexible def2-TZVPP basis set[7] and including implicit solvation effects with the COSMO model[8] for $CHCl_3$.

Relative free energies ($\Delta G(conf)$) of conformers were calculated according to equation (1) with respect to the most stable conformer of each of the two compounds.

$$\Delta G_{298}(total) = \Delta E(PW6B95\text{-}D3/def2\text{-}TZVPP+COSMO(CHCl_3)) + \Delta G^{RRHO}(GFN2\text{-}xTB) \quad (1)$$

The Boltzmann weight was determined according to the relative free energies:

$$p_{(Conf)} = [\exp(-G_{(conf)}/(RT)) \;/\; (\textstyle\sum_{(all\ Conf)} \exp(-G_{(conf)}/(RT)))] \cdot 100 \quad (2)$$

In general, this procedure revealed several conformations within a very narrow range of free energy (<1 kcal/mol), except for **6a**, for which the second best conformer is 1.9 kcal/mol less stable. The presence of several conformers would aggravate the structural analysis in solution by NMR or other experimental techniques. We have not filtered conformers which converged to similar structures during any stage of this procedure, therefore some conformations appear twice in the final list of conformers. This does not change the qualitative outcome of the conformational search.

Table S1: Relative free energies ΔG_{298}(total) (equation (2)) of conformers of **3a**.

Conformer[a]	G(total) [E_h]	ΔG_{298} (total)[kcal/mol]	p[b] [%]
CONF1	-1642.85468	1.06	4.75
CONF2	-1642.85593	0.27	17.86
CONF3	-1642.85626	0.07	25.11
**CONF4**[c]	**-1642.85637**	**0.00**	**28.22**
CONF5	-1642.85492	0.91	6.08
CONF7	-1642.85494	0.90	6.22
CONF9	-1642.85464	1.09	4.51
CONF10	-1642.85495	0.89	6.28

[a] only conformers within an energy window of ΔE=0-4 kcal/mol after the geometry optimization were considered in the final evaluation of ΔG. Conformers with a final Boltzmann weight below 1% are not listed.

[b] Boltzmann weight according to equation (2)

[c] this conformation is found in the solid state structure

Figure S1: Conformations of **3a** as listed in Table S1, optimized with PBEh-3c after pre-optimization with GFN2-xTB. Conformers converging to similar structures during the two-step procedure have not been eliminated.

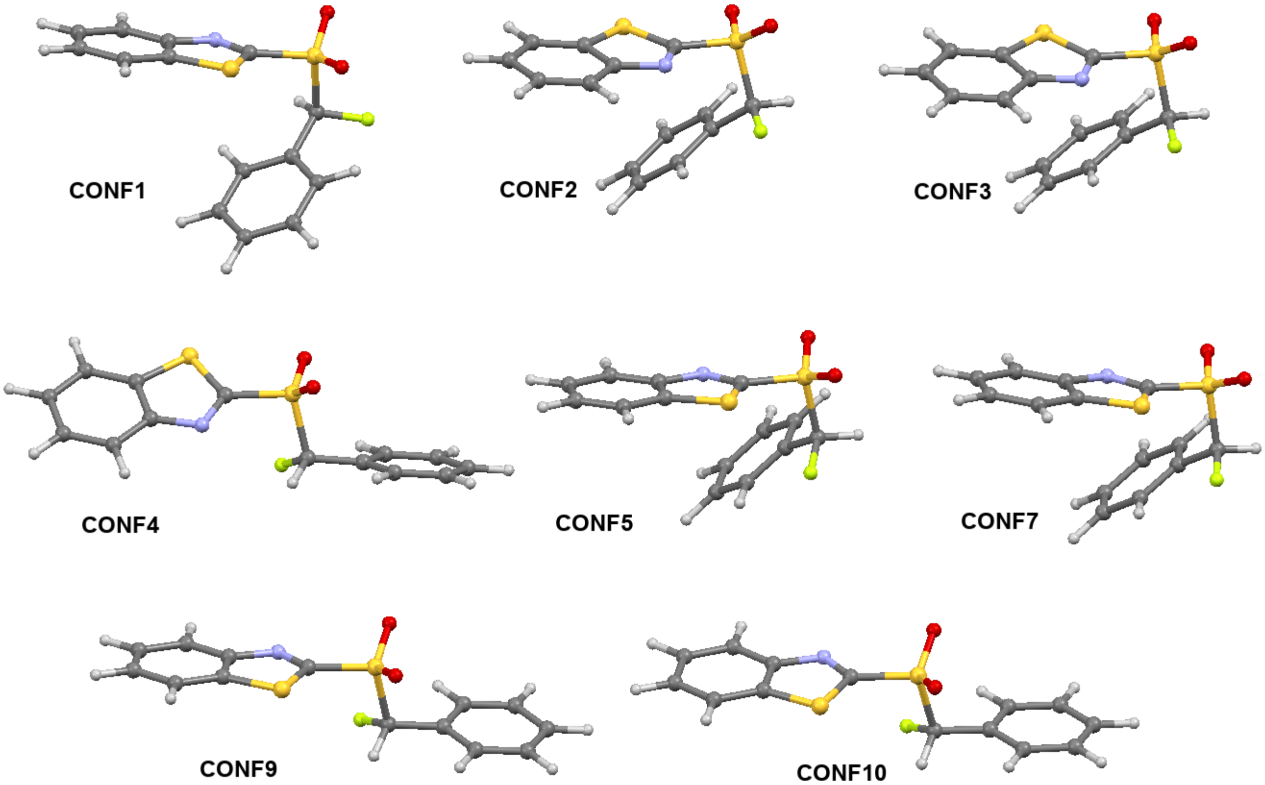

Table S2: Relative free energies ΔG_{298}(total) (equation (2)) of conformers of **3b**.

Conformer[a]	G(total) [E_h]	ΔG_{298} (total)[kcal/mol]	p[b] [%]
CONF1	-1682.20989	1.50	3.29
CONF4	-1682.21013	1.35	4.22
CONF5	-1682.21005	1.40	3.88
CONF11	-1682.21020	1.31	4.54
CONF12	-1682.20948	1.76	2.13
CONF13	-1682.21079	0.93	8.54
**CONF14**[c]	-1682.21228	0.00	41.13
CONF20	-1682.20994	1.47	3.45
CONF23	-1682.21118	0.69	12.84
CONF24	-1682.21098	0.81	10.43
CONF25	-1682.20994	1.47	3.47

[a] only conformers within an energy window of ΔE=0-4 kcal/mol after the geometry optimization were considered in the final evaluation of ΔG. Conformers with a final Boltzmann weight below 1% are not listed.

[b] Boltzmann weight according to equation (2)

[c] this conformation is found in the solid state structure

Figure S2: Conformations of **3b** as listed in Table S2, optimized with PBEh-3c after pre-optimization with GFN2-xTB. Conformers converging to similar structures during the two-step procedure have not been eliminated.

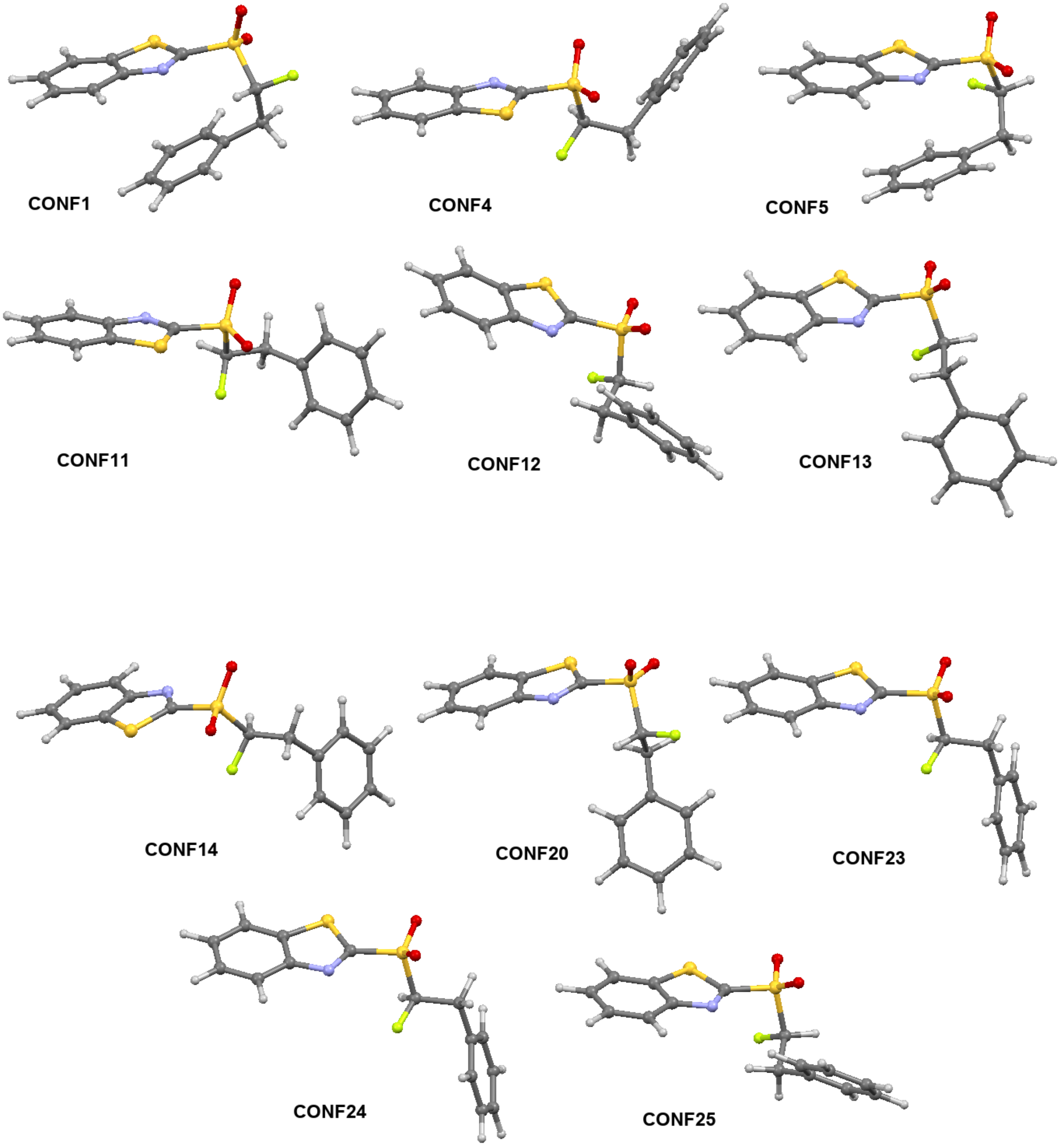

Table S3: Relative free energies ΔG_{298}(total) (equation (2)) of conformers of **3c**.

Conformer[a]	G(total) [E_h]	ΔG_{298} (total)[kcal/mol]	p[b] [%]
CONF1	-1796.72630	0.95	7.18
CONF2	-1796.72565	1.36	3.60
CONF3	-1796.72541	1.51	2.81
CONF5	-1796.72543	1.50	2.86
CONF6	-1796.72649	0.83	8.76
CONF7	-1796.72525	1.61	2.37
CONF8	-1796.72604	1.11	5.47
CONF9	-1796.72635	0.92	7.58
CONF10	-1796.72495	1.80	1.72
CONF11	-1796.72463	1.99	1.23
CONF13	-1796.72645	0.86	8.41
CONF14	-1796.72499	1.77	1.80
**CONF15[c]**	**-1796.72781**	**0.00**	**35.70**
CONF16	-1796.72520	1.64	2.24
CONF17	-1796.72478	1.90	1.43
CONF18	-1796.72564	1.36	3.60
CONF20	-1796.72475	1.92	1.39

[a] only conformers within an energy window of ΔE=0-4 kcal/mol after the geometry optimization were considered in the final evaluation of ΔG. Conformers with a final Boltzmann weight below 1% are not listed.

[b] Boltzmann weight according to equation (2)

[c] this conformation is found in the solid state structure

Figure S3: Conformations of **3c** as listed in Table S3, optimized with PBEh-3c after pre-optimization with GFN2-xTB. Conformers converging to similar structures during the two-step procedure have not been eliminated.

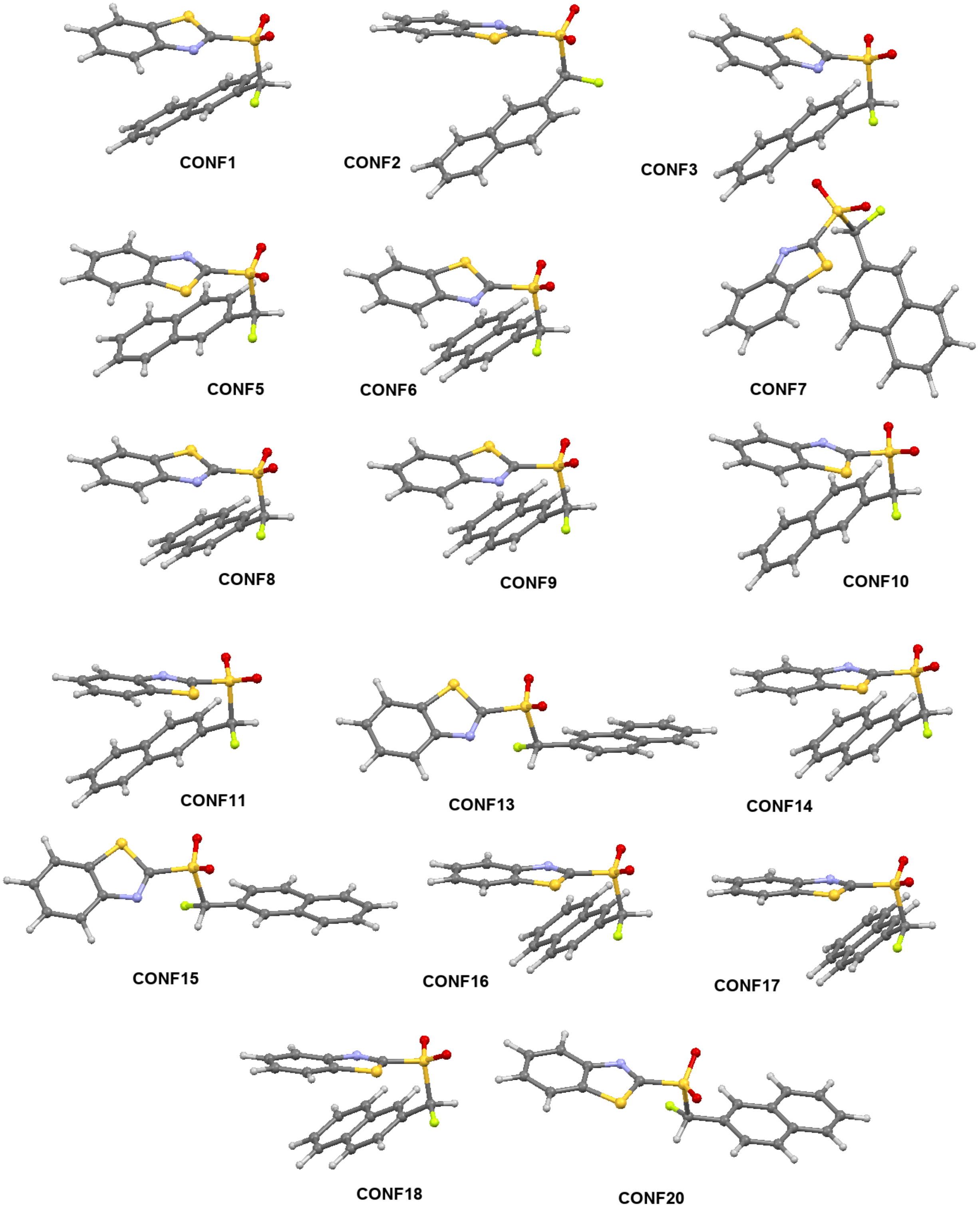

Table S4: Relative free energies ΔG_{298}(total) (equation (2)) of conformers of **6a**.

Conformer[a]	G(total) [E_h]	ΔG_{298} (total)[kcal/mol]	p[b] [%]
**CONF1**[c]	**-1567.51619**	**0.00**	**93.00**
CONF2	-1567.51320	1.88	3.92
CONF4	-1567.51288	2.07	2.81

[a] only conformers within an energy window of ΔE=0-4 kcal/mol after the geometry optimization were considered in the final evaluation of ΔG. Conformers with a final Boltzmann weight below 1% are not listed.

[b] Boltzmann weight according to equation (2)

[c] this conformation is found in the solid state structure

Figure S4: Conformations of **6a** as listed in Table S4, optimized with PBEh-3c after pre-optimization with GFN2-xTB.

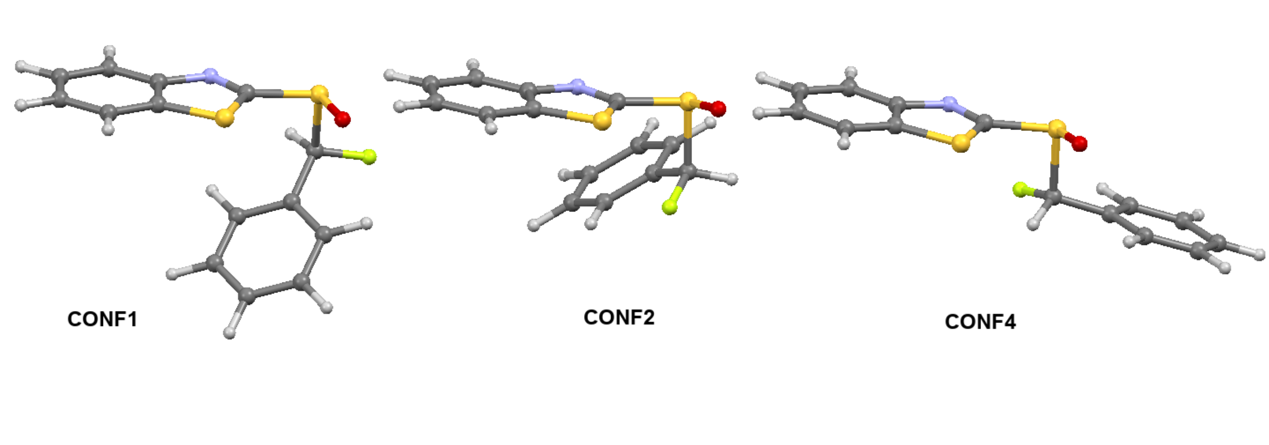

Table S5: Relative free energies ΔG_{298}(total) (equation (2)) of conformers of **6b**.

Conformer[a]	G(total) [E_h]	ΔG_{298} (total)[kcal/mol]	p[b] [%]
**CONF1**	**-1567.51522**	**0.00**	**46.04**
CONF2	-1567.51349	1.08	7.38
CONF3	-1567.51482	0.25	30.34
CONF4[c]	-1567.51410	0.70	14.06
CONF5	-1567.51213	1.94	1.75

[a] only conformers within an energy window of ΔE=0-4 kcal/mol after the geometry optimization were considered in the final evaluation of ΔG. Conformers with a final Boltzmann weight below 1% are not listed.

[b] Boltzmann weight according to equation (2)

[c] this conformation is found in the solid state structure

Figure S5: Conformations of **6b** as listed in Table S5, optimized with PBEh-3c after pre-optimization with GFN2-xTB. Conformers converging to similar structures during the two-step procedure have not been eliminated.

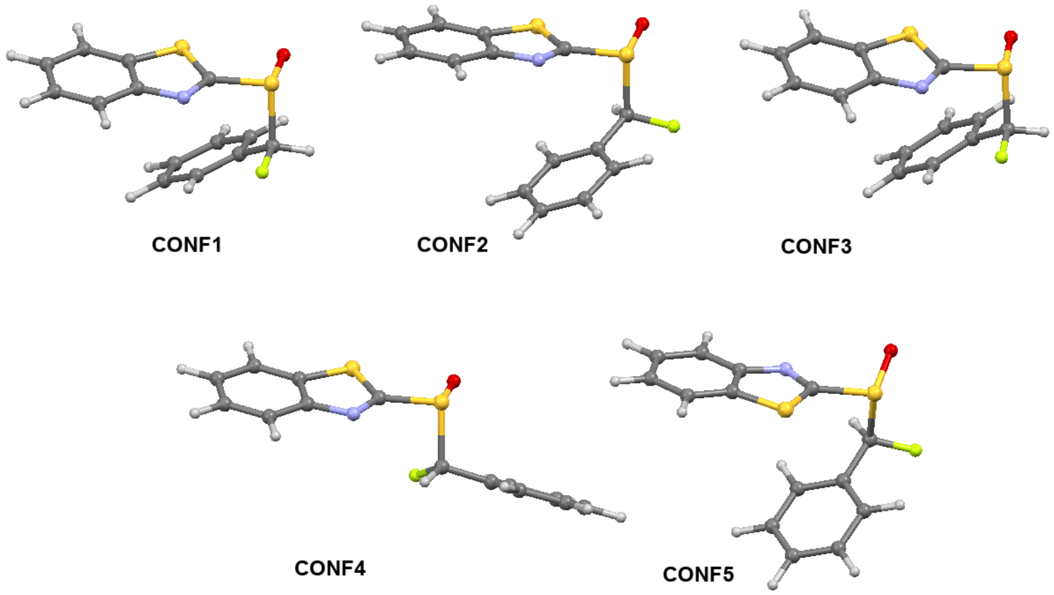

Table S6: Relative free energies ΔG_{298}(total) (equation (2)) of conformers of **7a**.

Conformer[a]	G(total) [E_h]	ΔG_{298} (total)[kcal/mol]	p[b] [%]
CONF1	-1606.86982	0.73	11.51
CONF2	-1606.86898	1.26	4.70
CONF4[c]	-1606.86851	1.55	2.85
**CONF8**	**-1606.87098**	**0.00**	**39.18**
CONF9	-1606.86915	1.15	5.65
CONF10	-1606.87065	0.21	27.66
CONF12	-1606.86892	1.29	4.41
CONF23	-1606.86846	1.58	2.71

[a] only conformers within an energy window of ΔE=0-4 kcal/mol after the geometry optimization were considered in the final evaluation of ΔG. Conformers with a final Boltzmann weight below 1% are not listed.

[b] Boltzmann weight according to equation (2)

[c] this conformation is found in the solid state structure

Figure S6: Conformations of **7a** as listed in Table S6, optimized with PBEh-3c after pre-optimization with GFN2-xTB. Conformers converging to similar structures during the two-step procedure have not been eliminated.

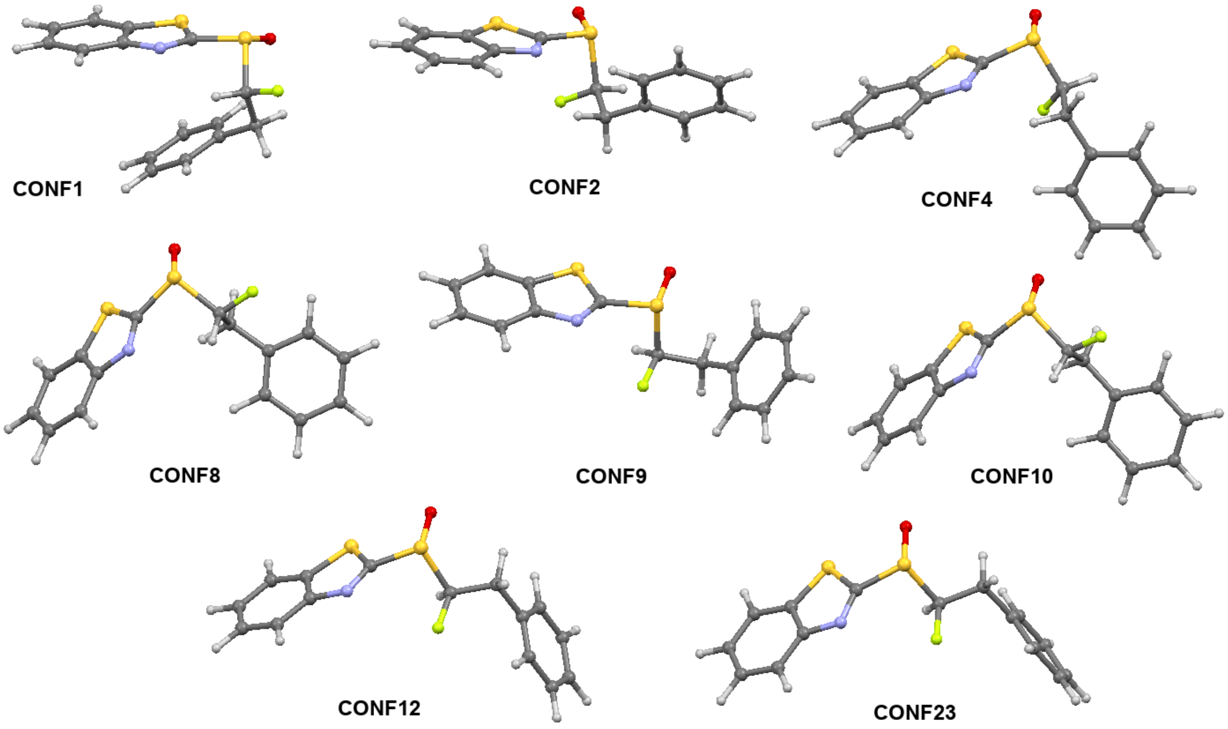

Table S7: Relative free energies ΔG_{298}(total) (equation (2)) of conformers of **7b**.

Conformer[a]	G(total) [E_h]	ΔG_{298} (total)[kcal/mol]	p[b] [%]
**CONF3**[c]	**-1606.86996**	**0.00**	**34.63**
CONF4	-1606.86773	1.40	3.28
CONF6	-1606.86789	1.30	3.85
CONF7	-1606.86995	0.01	34.21
CONF8	-1606.86693	1.90	1.40
CONF11	-1606.86780	1.36	3.51
CONF12	-1606.86736	1.63	2.22
CONF13	-1606.86746	1.57	2.46
CONF14	-1606.86771	1.41	3.19
CONF15	-1606.86809	1.17	4.78
CONF17	-1606.86817	1.13	5.18

[a] only conformers within an energy window of ΔE=0-4 kcal/mol after the geometry optimization were considered in the final evaluation of ΔG. Conformers with a final Boltzmann weight below 1% are not listed.

[b] Boltzmann weight according to equation (2)

[c] this conformation is found in the solid state structure

Figure S7: Conformations of **7b** as listed in Table S7, optimized with PBEh-3c after pre-optimization with GFN2-xTB. Conformers converging to similar structures during the two-step procedure have not been eliminated.

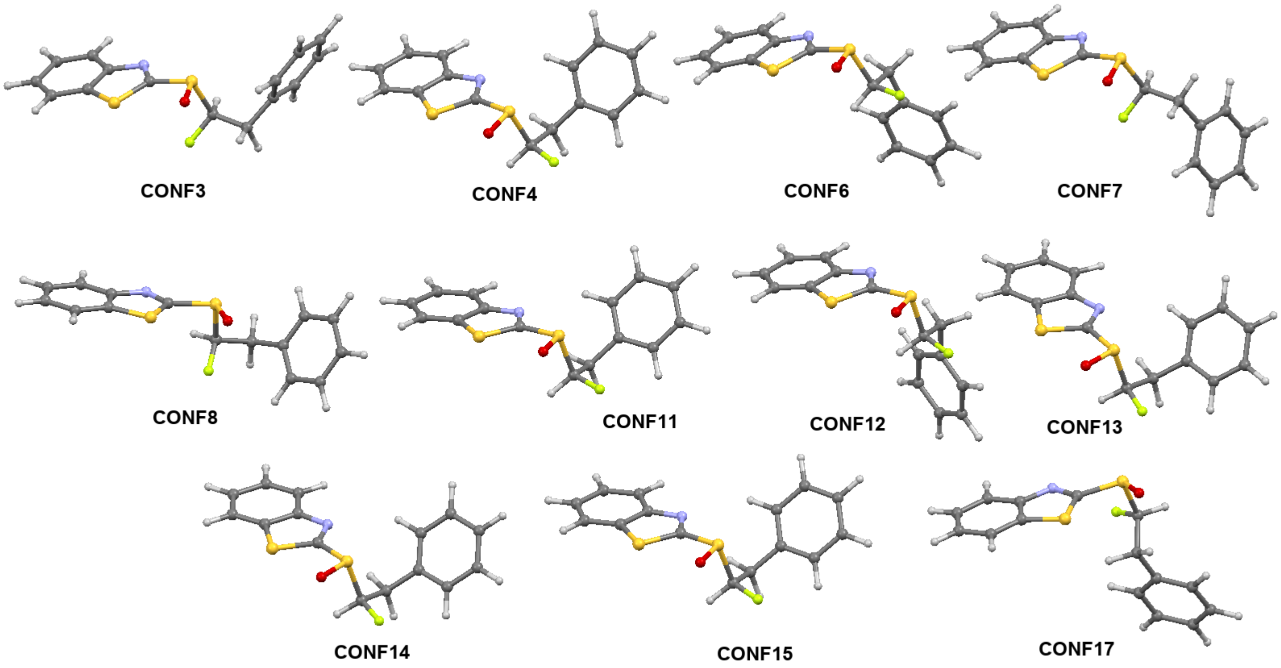

Table S8: Relative free energies ΔG_{298}(total) (equation (2)) of conformers of **8a**.

Conformer[a]	G(total) [E_h]	ΔG_{298} (total)[kcal/mol]	p[b] [%]
CONF1	-1721.38550	0.49	13.79
CONF2	-1721.38486	0.89	7.04
CONF3	-1721.38482	0.92	6.76
CONF4	-1721.38539	0.56	12.28
CONF5	-1721.38582	0.29	19.42
CONF6	-1721.38441	1.17	4.39
**CONF7**	**-1721.38628**	**0.00**	**31.77**
CONF11[c]	-1721.38367	1.64	2.00
CONF14	-1721.38388	1.51	2.49

[a] only conformers within an energy window of ΔE=0-4 kcal/mol after the geometry optimization were considered in the final evaluation of ΔG. Conformers with a final Boltzmann weight below 1% are not listed.

[b] Boltzmann weight according to equation (2)

[c] this conformation is found in the solid state structure

Figure S8: Conformations of **8a** as listed in Table S8, optimized with PBEh-3c after pre-optimization with GFN2-xTB. Conformers converging to similar structures during the two-step procedure have not been eliminated.

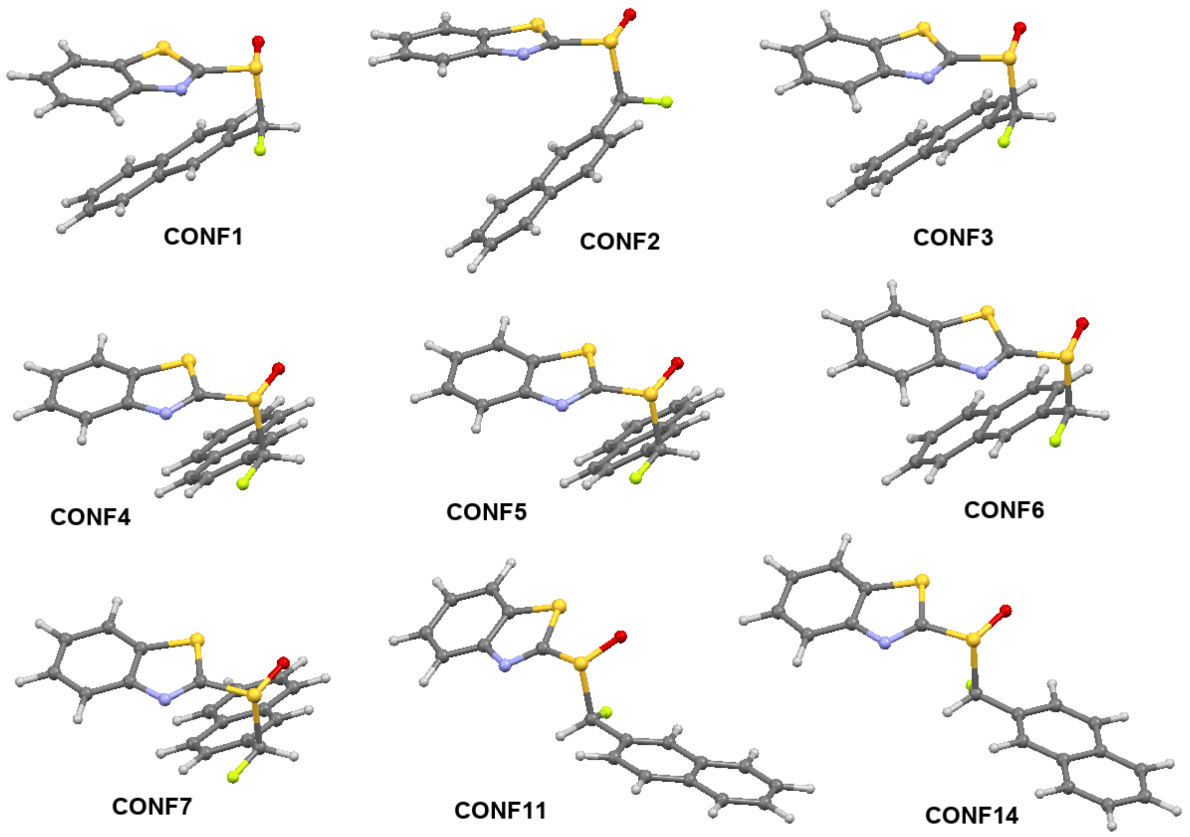

Table S9: Relative free energies ΔG_{298}(total) (equation (2)) of conformers of **5b**.

Conformer[a]	G(total) [E_h]	ΔG_{298} (total)[kcal/mol]	p[b] [%]
CONF2[c]	-1531.56631	0.72	7.16
CONF4	-1531.56732	0.08	20.89
CONF7	-1531.56704	0.25	15.59
CONF8	-1531.56484	1.64	1.50
CONF15	-1531.56675	0.44	11.46
CONF16	-1531.56682	0.40	12.27
CONF17	-1531.56512	1.46	2.04
**CONF18**	**-1531.56745**	**0.00**	**23.94**
CONF19	-1531.56454	1.82	1.10

[a] only conformers within an energy window of ΔE=0-4 kcal/mol after the geometry optimization were considered in the final evaluation of ΔG. Conformers with a final Boltzmann weight below 1% are not listed.

[b] Boltzmann weight according to equation (2)

[c] this conformation is found in the solid state structure

Figure S9: Conformations of **5b** as listed in Table S9, optimized with PBEh-3c after pre-optimization with GFN2-xTB. Conformers converging to similar structures during the two-step procedure have not been eliminated.

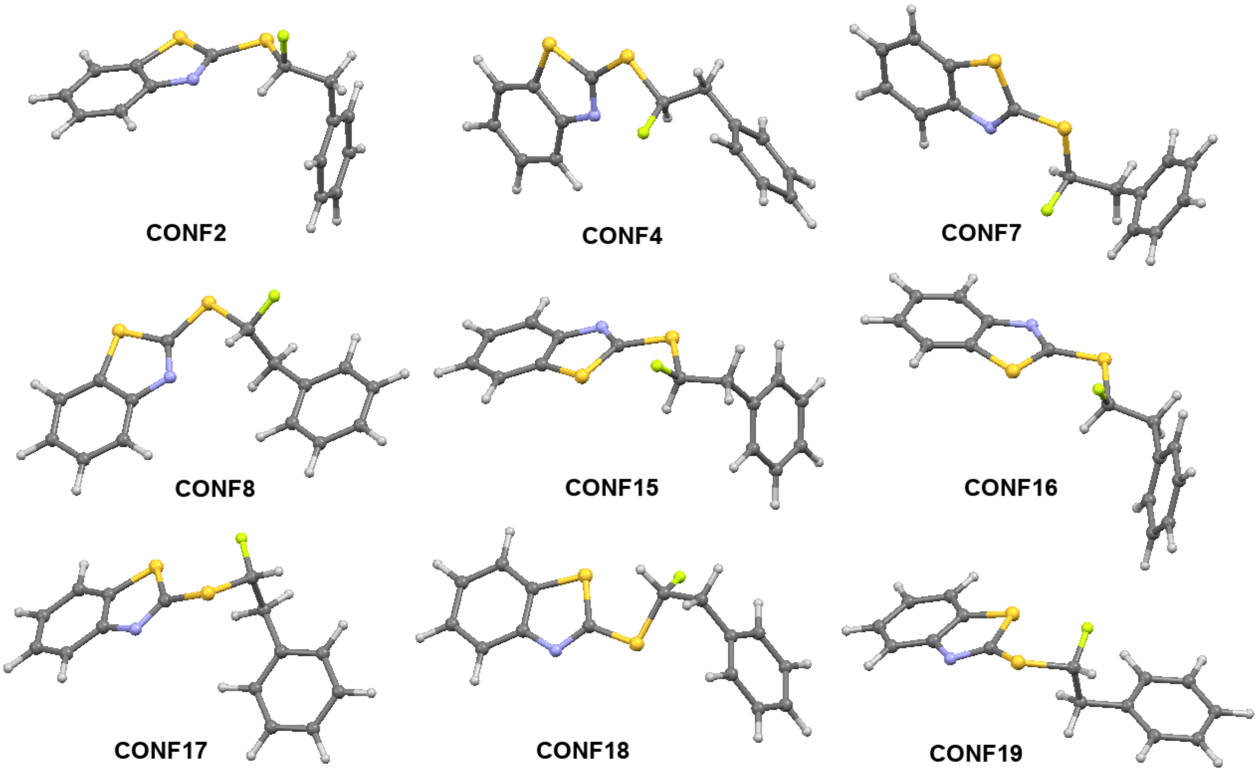

Model compounds

The energy profile of the S-C(F) bond rotation in model compounds **A** and **B** (Figure 12) was performed with the ORCA program[9] using the meta-GGA functional TPSS[10], the D3 dispersion correction[6] and the def2-TZVP[7] basis set. All degrees of freedom were optimized while keeping the C-S(O)-C(F)-C dihedral angle fixed in steps of 10.0 degrees between 0 to 360 degrees.

The conformers of **A** and **B** and the model compound **C** were optimized with the Turbomole[11] program the same functional and basis set. NBO analysis[12] of these conformers was conducted with NBO 6.0[13] in conjunction with ORCA. Second order perturbation analysis of donor-acceptor interactions of the sulfur lone pair with vicinal σ* bonds of the fluorinated α carbon atom are listed in Table S11.

Optimized cartesian coordinates and energies (TPSS-D3/def2-TZVP) of the conforrmers of **A** and **B**

A-1 E=-691.9675345013 E_h

S	0.1997373	-0.8706393	-0.6081120
O	0.6872525	-0.1600441	-1.8294096
C	-1.6077539	-0.4284397	-0.4106447
H	-2.0529306	-0.5035908	-1.4099261
C	-0.1200061	-2.6062516	-1.1047368
H	-0.8548436	-2.6307605	-1.9142451
H	-0.4483140	-3.1931877	-0.2435976
H	0.8408825	-2.9790747	-1.4652197
C	-2.3290180	-1.2216703	0.6576059
H	-2.5146075	-2.2468046	0.3264666
H	-3.2938123	-0.7459544	0.8593261
H	-1.7514254	-1.2411372	1.5870796
F	-1.5995395	0.9230786	-0.0568089

A-2 E=-691.9705670885 E_h

S	0.1616707	-0.9506866	-0.4439865
O	0.7458878	-0.7514936	0.9210100

C	-1.6419994	-0.4362801	-0.3799957
H	-2.0622878	-0.6856599	-1.3623315
C	0.7074744	0.4444246	-1.4840683
H	0.4964995	1.3770143	-0.9590744
H	0.1881841	0.4038226	-2.4461062
H	1.7811664	0.3102232	-1.6276513
C	-2.3035178	-1.1542487	0.7719485
H	-2.2650199	-2.2365965	0.6110346
H	-3.3488460	-0.8429001	0.8503097
H	-1.7757973	-0.9184749	1.7002149
F	-1.7691394	0.9464626	-0.2056103

A-3 E=-691.9703199711 E_h

S	0.1495361	-1.0036050	-0.4556482
O	0.1136724	-2.5040834	-0.4039558
C	-1.6524176	-0.4654465	-0.3359949
H	-2.0540073	-0.6972834	-1.3280152
C	0.6642665	-0.4043903	1.1899596
H	0.1577751	-0.9884656	1.9611779
H	0.4315783	0.6605062	1.2703580
H	1.7429810	-0.5632591	1.2468084
C	-2.4093512	-1.1533775	0.7711017
H	-2.2030088	-2.2274719	0.7222153
H	-3.4843221	-0.9893277	0.6504625
H	-2.1031581	-0.7719296	1.7484601
F	-1.6676403	0.9275999	-0.1881040

B-1 E=-691.9696005066 E_h

S	0.2333772	-0.8824612	-0.7695690
O	0.6534809	-0.3647821	-2.1106359
C	-1.5832923	-0.4515946	-0.5357872
C	-0.0630588	-2.6817389	-0.9561969

H	-0.7511822	-2.8618802	-1.7853328
H	-0.4502074	-3.0896491	-0.0181609
H	0.9112947	-3.1204069	-1.1795320
F	-1.5878586	0.7943951	0.1019701
C	-2.3066333	-0.3516087	-1.8548974
H	-1.7335925	0.2747811	-2.5435678
H	-3.2966053	0.0839452	-1.6909944
H	-2.4300605	-1.3422202	-2.3024967
H	-2.0083927	-1.1794260	0.1645561

B-2 E=-691.9685754136 E_h

S	0.2120443	-0.9288086	-0.4779845
O	0.7408838	-0.7371650	0.9086477
C	-1.6079079	-0.4761834	-0.4548222
C	0.7564440	0.5104453	-1.4612344
H	0.4588448	1.4219158	-0.9407549
H	0.3260700	0.4618792	-2.4650658
H	1.8437239	0.4313619	-1.5158467
F	-1.7815193	0.8774397	-0.1542283
C	-2.3059958	-0.8176682	-1.7536577
H	-2.1699131	-1.8784094	-1.9853765
H	-1.9119834	-0.2211816	-2.5813826
H	-3.3767469	-0.6137302	-1.6596008
H	-1.9865892	-1.0460384	0.4008853

B-3 E=-691.9713251409 E_h

S	0.2328774	-0.9710598	-0.4796812
O	0.1622396	-2.4708697	-0.4754075
C	-1.5528408	-0.4028069	-0.4324085
C	0.6127210	-0.4341818	1.2224041
H	-0.0243511	-0.9934637	1.9129767
H	0.4428206	0.6425226	1.3013006

```
H     1.6638406   -0.6695985    1.4003815
F    -1.5522531    0.9912031   -0.2863482
C    -2.2522585   -0.8113825   -1.7041836
H    -1.7921233   -0.3208954   -2.5671830
H    -3.3100109   -0.5362839   -1.6554484
H    -2.1706143   -1.8958517   -1.8238163
H    -1.9988187   -0.8398572    0.4686096
```

Table S11: NBO analysis of the three conformers of **A** and **B**. Second order perturbation theory energies for donor-acceptor interactions of the sulfur lone pair with vicinal σ* orbital of the fluorinated α carbon atom and the oxygen and fluorine lone pairs with the antibonding σ* orbital of the S-C(F) bond.

Conformer	E_2^{PT} [kcal/mol]				
	S:→σ*(C-F)	S:→σ*(C-H)	S:→σ*(C-C)	O:→σ*(S-C(F))	F:→σ*(S-C(F))
**A-1**	0.83	0.55	---	10.81	4.35
**A-2**	3.45	1.23	---	11.09	4.03
**A-3**	---	---	1.57	10.33	3.90
**B-1**	1.02	---	1.26	11.00	3.92
**B-2**	3.33			11.07	4.36
**B-3**	---	0.88	---	9.92	4.00

Model compound C for $n_N \rightarrow \sigma^*_{C-F}$ interaction

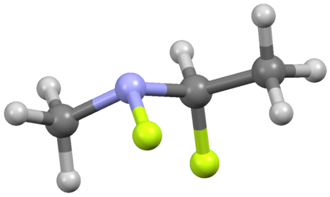

C

Optimized Cartesian Coordinates (TPSS-D3/def2-TZVP) of the model compound **C**

N	0.7321049	-0.2844695	-0.2364030
C	-0.6634194	-0.0201646	-0.0326486
H	-1.1161092	-0.2090096	-1.0143336
C	1.4199303	0.6802269	-1.0942926
H	1.3662233	1.7006303	-0.7015098
H	0.9278046	0.6342443	-2.0712564
H	2.4571210	0.3601607	-1.2009679
C	-1.2815152	-0.8639650	1.0605058
H	-1.1108238	-1.9232343	0.8506031
H	-2.3558804	-0.6706166	1.0980965
H	-0.8338931	-0.6124066	2.0230603
F	-0.9295081	1.3497907	0.2549705
F	1.3879652	-0.1411867	1.0641757

NBO: second order perturbation theory energy for donor-acceptor interaction in **C**

$$N{:}\rightarrow\sigma^*(C\text{-}F) \quad E_2^{PT} = 11.2 \text{ kcal/mol}$$

References

[1] (a) *APEX3* (**2016**), *SAINT* (**2015**) and *SADABS* (**2015**), Bruker AXS Inc., Madison, Wisconsin, USA. (b) Sheldrick, G. M. SHELXT – Integrated space-group and crystal-structure determination, *Acta Cryst.* **2015**, *A71*, 3-8. (c) Sheldrick, G. M. Crystal structure refinement with SHELXL, *Acta Cryst.*, **2015**, *C71 (1)*, 3-8. (d) *XP – Interactive molecular graphics, Version 5.1,* Bruker AXS Inc., Madison, Wisconsin, USA, **1998**. (e) Hooft, R. W. W.; Nonius, B. V. *COLLECT, Program for Collecting Data on CCD Area Detectors*, **1998**; Delft, The Netherlands. (f) Otwinowski Z.; Minor, W. Processing of X-ray diffraction data collected in oscillation mode, *Methods Enzymol.* **1997**, *276*, 307 – 326. (g) Otwinowski Z.; Borek D; Majewski, W.; Minor, W. Multiparametric scaling of diffraction intensities. *Acta Crystallogr. Sect. A* **2003**, *59*, 228 – 234.

[2] Grimme, S.; Bannwarth, C.; Dohm, S.; Hansen, A.; Pisarek, J.; Pracht, P.; Seibert, J.; Neese, F. Fully Automated Quantum-Chemistry-Based Computation of Spin-Spin-Coupled Nuclear Magnetic Resonance Spectra. *Angew. Chem. Int. Ed.* **2017**, *56*, 14763-14769.

[3] Bannwarth, C.; Ehlert, S.; Grimme, S. GFN2-xTB-An Accurate and Broadly Parametrized Self-Consistent Tight-Binding Quantum Chemical Method with Multipole Electrostatics and Density-Dependent Dispersion Contributions. *J. Chem. Theory. Comput.* **2019**, *15*, 1652-1671.

[4] Grimme, S.; Brandenburg, J. G.; Bannwarth, C.; Hansen, A. Consistent structures and interactions by density functional theory with small atomic orbital basis sets. *J. Chem. Phys.* **2015**, *143*, 054107

[5] Zhao, Y.; Truhlar, D. G. Design of Density Functionals That Are Broadly Accurate for Thermochemistry, Thermochemical Kinetics, and Nonbonded Interactions. *J. Phys. Chem. A* **2005**, *109*, 5656-5667.

[6] a) Grimme, S.; Antony, J.; Ehrlich, S.; Krieg, H. A Consistent and Accurate Ab Initio Parametrization of Density Functional Dispersion Correction (DFT-D) for the 94 Elements H-Pu. *J. Chem. Phys.* **2010**, *132***,** 154104. b) Grimme, S.; Ehrlich, S.; Goerigk, L. Effect of the Damping Function in Dispersion Corrected Density Functional Theory. *J. Comput. Chem.* **2011**, *32*, 1456–1465.

[7] Weigend F.; Ahlrichs R. Balanced basis sets of split valence, triple zeta valence and quadruple zeta valence quality for H to Rn: Design and assessment of accuracy. *Phys. Chem. Chem. Phys.* **2005**, *7*, 3297–3305.

[8] Klamt, A.; Schüürmann, G. COSMO: a new approach to dielectric screening in solvents with explicit expressions for the screening energy and its gradient. *J. Chem. Soc. Perkin Trans. 2*, **1993**, 799-805.

[9] Neese, F. The ORCA program system, *Wiley Interdisciplinary Reviews: Computational Molecular Science,* **2012**, *2*, 73-78.

[10] Tao, J.; Perdew, J. P.; Staroverov V. N.; Scuseria, G. E. Climbing the Density Functional Ladder: Nonempirical Meta-Generalized Gradient Approximation Designed for Molecules and Solids. *Phys. Rev. Lett.* **2003**, *91*, 146401.

[11] TURBOMOLE V7.4 2019, a development of University of Karlsruhe and Forschungszentrum Karlsruhe GmbH, 1989-2007, TURBOMOLE GmbH, since 2007; available from http://www.turbomole.com.

[12] Reed A. E.; Weinstock R. B.; Weinhold F. Natural population analysis. *J. Chem. Phys.* **1985**, *83*, 735–746.

[13] NBO 6.0. Glendening, E. D.; Badenhoop, J. K.; Reed, A. E.; Carpenter, J. E.; Bohmann, J. A.; Morales, C. M.; Landis, C. R.; Weinhold F. (Theoretical Chemistry Institute, University of Wisconsin, Madison, WI, 2013); http://nbo6.chem.wisc.edu/
